# Greater than recommended stiffness and power setting of a stance-phase powered leg prosthesis can improve step-to-step transition work and effective foot length ratio during walking in people with transtibial amputation

**DOI:** 10.3389/fbioe.2024.1336520

**Published:** 2024-07-01

**Authors:** Joshua R. Tacca, Zane A. Colvin, Alena M. Grabowski

**Affiliations:** ^1^ Paul M. Rady Department of Mechanical Engineering, University of Colorado, Boulder, CO, United States; ^2^ Department of Integrative Physiology, University of Colorado, Boulder, CO, United States; ^3^ Department of Veterans Affairs, Eastern Colorado Healthcare System, Denver, CO, United States

**Keywords:** prostheses, amputee, bionic, biomechanics, individual limb work, roll-over shape

## Abstract

People with unilateral transtibial amputation (TTA) using a passive-elastic prosthesis exhibit lower positive affected leg trailing work (AL_trail_ W_pos_) and a greater magnitude of negative unaffected leg leading work (UL_lead_ W_neg_) during walking than non-amputees, which may increase joint pain and osteoarthritis risk in the unaffected leg. People with TTA using a stance-phase powered prosthesis (e.g., BiOM, Ottobock, Duderstadt, Germany) walk with increased AL_trail_ W_pos_ and potentially decreased magnitude of UL_lead_ W_neg_ compared to a passive-elastic prosthesis. The BiOM includes a passive-elastic prosthesis with a manufacturer-recommended stiffness category and can be tuned to different power settings, which may change AL_trail_ W_pos,_ UL_lead_ W_neg,_ and the prosthesis effective foot length ratio (EFLR). Thirteen people with TTA walked using 16 different prosthetic stiffness category and power settings on a level treadmill at 0.75–1.75 m/s. We constructed linear mixed effects models to determine the effects of stiffness category and power settings on AL_trail_ W_pos,_ UL_lead_ W_neg,_ and EFLR and hypothesized that decreased stiffness and increased power would increase AL_trail_ W_pos_, not change and decrease UL_lead_ W_neg_ magnitude, and decrease and not change prosthesis EFLR, respectively. We found there was no significant effect of stiffness category on AL_trail_ W_pos_ but increased stiffness reduced UL_lead_ W_neg_ magnitude, perhaps due to a 0.02 increase in prosthesis EFLR compared to the least stiff category. Furthermore, we found that use of the BiOM with 10% and 20% greater than recommended power increased AL_trail_ W_pos_ and decreased UL_lead_ W_neg_ magnitude at 0.75–1.00 m/s. However, prosthetic power setting depended on walking speed so that use of the BiOM increased UL_lead_ W_neg_ magnitude at 1.50–1.75 m/s compared to a passive-elastic prosthesis. Ultimately, our results suggest that at 0.75–1.00 m/s, prosthetists should utilize the BiOM attached to a passive-elastic prosthesis with an increased stiffness category and power settings up to 20% greater than recommended based on biological ankle values. This prosthetic configuration can allow people with unilateral transtibial amputation to increase AL_trail_ W_pos_ and minimize UL_lead_ W_neg_ magnitude, which could reduce joint pain and osteoarthritis risk in the unaffected leg and potentially lower the metabolic cost of walking.

## 1 Introduction

People with unilateral transtibial amputation typically walk using passive-elastic prosthetic feet that cannot fully replicate the mechanical function of biological lower limbs. Passive-elastic prostheses can store and return elastic energy during the stance phase of level-ground walking but cannot generate net positive mechanical work. Such prostheses return only one-half the positive mechanical work typically generated by the soleus and gastrocnemius muscles during level-ground walking at 1.5 m/s (Zmitrewicz et al., 2007), and thus people with unilateral transtibial amputation walk with altered mechanical work compared to non-amputees, particularly during the step-to-step transition phase of walking. During the step-to-step transition, people with unilateral transtibial amputation using passive-elastic prostheses exhibit lower positive affected trailing leg work and a greater magnitude of negative unaffected leading leg work compared to non-amputees at 0.7–1.75 m/s (Zelik et al., 2011; Herr and Grabowski, 2012; Adamczyk and Kuo, 2015). Ultimately, a greater magnitude of unaffected leading leg work may be related to the higher prevalence of joint pain and osteoarthritis in the unaffected leg and backs of people with unilateral transtibial amputation compared to non-amputees (Kulkarni et al., 2005; Norvell et al., 2005; Struyf et al., 2009; Morgenroth et al., 2011; Morgenroth et al., 2012). Prosthetic mechanical properties and designs that better replicate biological muscle function could therefore normalize step-to-step transition work and reduce the risk and burden of joint pain and osteoarthritis in people with transtibial amputation.

The stiffness of a prosthesis is a mechanical property that could affect step-to-step transition work. Passive-elastic prosthetic feet are characterized by stiffness categories that are recommended by each manufacturer based on a person’s bodyweight and impact or activity level (Össur, 2015; Ruxin et al., 2022), where a greater numerical stiffness category corresponds with a stiffer prosthesis. The use of a prosthetic foot with different stiffness can affect the biomechanics of people with unilateral transtibial amputation during level-ground walking (Klodd et al., 2010; Fey et al., 2011; Zelik et al., 2011; Major et al., 2014; Adamczyk et al., 2017; Halsne et al., 2020; Rogers-Bradley et al., 2024). For example, when people with unilateral transtibial amputation walk at a range of speeds (0.6–1.5 m/s) using a less stiff experimental prosthetic foot, they have greater prosthesis energy return and trailing affected leg mechanical work compared to when they use a stiffer prosthetic foot (Zelik et al., 2011; Adamczyk et al., 2017; Rogers-Bradley et al., 2024). Moreover, the increase in trailing affected leg work between the less stiff and stiffer prostheses is greater at faster walking speeds (Adamczyk et al., 2017). Since mechanical models of walking predict that increasing affected trailing leg work can reduce the magnitude of negative unaffected leading leg work for people with unilateral transtibial amputation (Adamczyk and Kuo, 2015), use of a less stiff prosthesis may allow people with unilateral transtibial amputation to walk with a higher magnitude of affected trailing leg work and a lower magnitude of unaffected leading leg work compared to when they use a stiffer prosthesis. Adamczyk et al. found that when people with unilateral amputation walked at a range of speeds (0.6–1.5 m/s), there was a weak correlation between a less stiff prosthesis and a reduced magnitude of unaffected leading leg work (Adamczyk et al., 2017). Ultimately, when people with unilateral transtibial amputation walk using a less stiff prosthetic foot they could exhibit step-to-step transition work similar to that of non-amputees at a range of speeds (Morgenroth et al., 2011).

Step-to-step transition work can also be influenced by the effective arc shape of the prosthetic foot, termed its roll-over shape (Adamczyk et al., 2006). During the stance phase of walking, roll-over shape is defined as the arc created by the location of the center of pressure relative to the shank of the leg (Hansen et al., 2004). The roll-over shape of the foot can affect the mechanical work performed during the step-to-step transition by influencing the required directional change of the center of mass velocity (Adamczyk et al., 2006). A previous study found that when non-amputees walked in boots attached on top of wooden arcs with different roll-over shape radii, increasing the radius decreased the magnitude of negative work done by the leading leg during the step-to-step transition (Adamczyk et al., 2006). One way to quantify roll-over shape is by calculating the effective foot length ratio, which is the ratio between the horizontal distance from the heel to the most anterior portion of the roll-over shape (effective foot length) and the horizontal distance from the heel to the end of the toe (foot length) (Hansen et al., 2004). The effective foot length ratio is greater for roll-over shapes with larger radii. However, effective foot length ratios of passive-elastic prostheses are typically lower than those of biological feet (Hansen et al., 2004). Prosthetic feet with greater effective foot length ratios may allow people with unilateral transtibial amputation to walk with a lower magnitude of leading unaffected leg negative work during the step-to-step transition. The stiffness of a passive-elastic prosthetic foot can also affect its effective foot length ratio. For example, less stiff compared to more stiff prosthetic feet have lower effective foot length ratios (Klodd et al., 2010). There may be a prosthetic foot stiffness category that matches the effective foot length ratio of biological feet and allows people with transtibial amputation to walk with unaffected leading leg step-to-step transition work similar to that of non-amputees.

**FIGURE 1:**
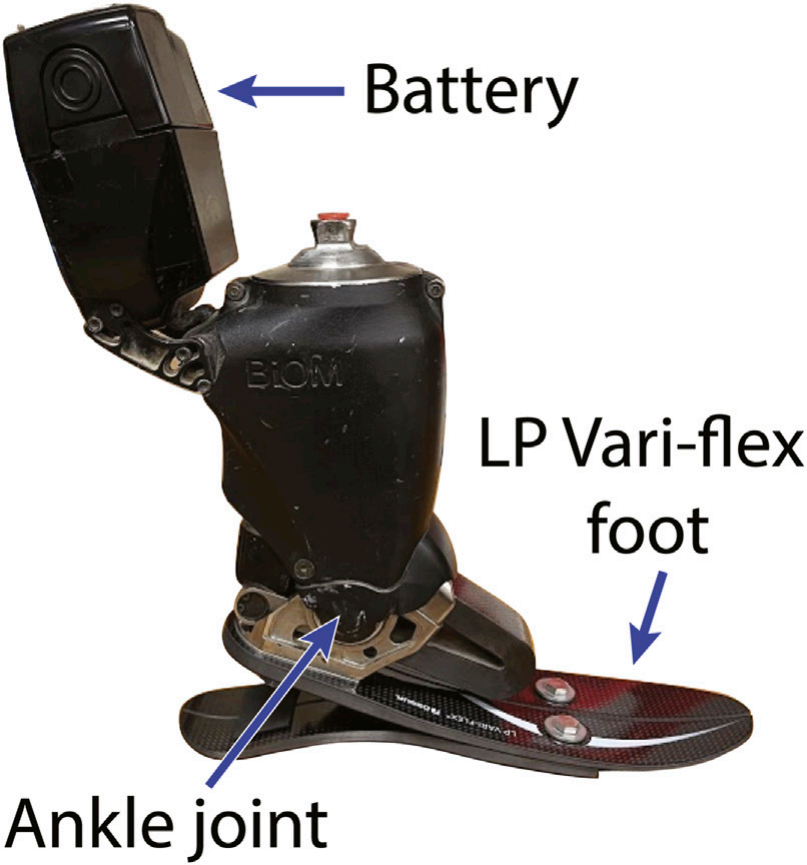
The BiOM powered ankle-foot prosthesis. The BiOM includes an Össur Low Profile (LP) Vari-flex passive-elastic prosthetic foot and uses battery-power to generate net positive mechanical work about the prosthetic ankle joint during the stance phase of walking. The BiOM includes a series elastic actuator that generates power about the ankle joint that is adjusted by a torque sensor and uses positive torque feedback so that an increase in the sensed torque about the prosthetic ankle joint increases the magnitude of power delivered. Thus, the power provided by the BiOM can adapt to different walking speeds.

Several lower limb powered prostheses have been designed to increase the mechanical work done by the prosthesis during the stance phase of walking compared to passive-elastic prostheses, including powered ankle-foot prostheses and prosthetic emulators (Hansen et al., 2004; Adamczyk et al., 2006; Au et al., 2009; Quesada et al., 2016). The BiOM (now Ottobock Empower, Duderstadt, Germany) (Figure 1) is the only commercially-available, battery-powered ankle-foot prosthesis that generates net positive work during the stance phase of walking (Au et al., 2009; Eilenberg et al., 2010). Herr and Grabowski found that when people with unilateral transtibial amputation walked at a range of speeds (0.75–1.75 m/s), use of the BiOM resulted in increased affected trailing leg positive work and a decreased magnitude of unaffected leading leg negative work compared to a passive-elastic prosthesis and the magnitude of positive and negative step to step transition work was comparable to that of non-amputees (Herr and Grabowski, 2012). However, Russell-Esposito et al. found that when people with unilateral transtibial amputation walked at 1.2 m/s use of the BiOM resulted in increased affected trailing leg positive work, but did not change the magnitude of unaffected leading leg negative work compared to a passive-elastic prosthesis (Esposito et al., 2016). Because the effective foot length ratio of the prosthesis can affect the magnitude of unaffected leading leg negative work (Adamczyk et al., 2006), use of the BiOM compared to a passive-elastic prosthesis may result in a different effective foot length ratio and step-to-step transition work during level ground walking at a range of speeds.

The power settings of the BiOM powered ankle-foot prosthesis presumably affect step-to-step transition work during level ground walking and determining these effects could provide objective metrics to guide prosthesis selection and reduce secondary injury risk. The settings of the BiOM powered ankle-foot prosthesis can be adjusted or tuned to each individual user to exhibit different prosthetic power outputs (Eilenberg et al., 2010; Ingraham et al., 2018; iWalk Inc, 2013). In particular, the power settings of the BiOM can be adjusted from 0% (no power) to 100% (maximum power setting) (Ingraham et al., 2018; iWalk Inc, 2013). The manufacturer recommends adjusting the power settings so that the net ankle work per step measured by onboard sensors in the BiOM is within a 95% confidence interval of non-amputee net ankle work for a given walking speed (Ingraham et al., 2018; iWalk Inc, 2013). The BiOM uses positive torque feedback control so that an increase in the sensed torque about the prosthetic ankle of the BiOM increases the magnitude of power delivered by the prosthesis, which allows the BiOM to adapt the power delivered at different walking speeds (Herr and Grabowski, 2012). In experimental studies, the BiOM settings have been tuned based on the prosthetist and manufacturer recommendations, user feedback, and/or to match non-amputee ankle biomechanics (Herr and Grabowski, 2012; Esposito et al., 2016; DAndrea et al., 2014; Ferris et al., 2012; Grabowski and DAndrea, 2013; Russell Esposito and Wilken, 2014; Gardinier et al., 2018; Kim et al., 2021). However, only one study has examined the effects of tuning the BiOM to several different power settings (Ingraham et al., 2018). Ingraham et al. examined people with a transtibial amputation walking on level ground at ∼1.2 m/s using the BiOM with different power settings and found that the power setting that minimized metabolic cost of transport resulted in an 8.8% reduction in the metabolic cost compared to the power setting chosen by the prosthetist (Ingraham et al., 2018). The best tested setting was on average 86% and the prosthetist chosen power setting was 42% on average (Ingraham et al., 2018). However, the effects of using different BiOM power settings on step-to-step transition work and effective foot length ratio are unknown.

Powered prosthetic emulators have been used to test the effects of different prosthetic ankle power settings on step-to-step transition work (Caputo and Collins, 2014a; Quesada et al., 2016). Powered prosthetic emulators have been designed to mimic powered ankle-foot prostheses with an off-board motor and allow for the power generated by the prosthesis to be adjusted (Caputo and Collins, 2014a; Quesada et al., 2016). When walking using a powered prosthetic emulator at 1.25 m/s with peak prosthetic ankle power settings that ranged from ½ to 2 times the values typically returned by a recommended stiffness category passive-elastic prosthesis, increasing prosthetic power resulted in increased affected trailing leg positive work but did not decrease the magnitude of unaffected leading leg negative work for people with unilateral transtibial amputation (Quesada et al., 2016). However, differences in prosthetic foot design such as its weight (1 kg Emulator foot vs 2 kg BiOM) (Caputo and Collins, 2014b) and tuning methods between the prosthetic emulator and the BiOM may result in different responses (Quesada et al., 2016). Since the BiOM is commercially available, understanding how different power settings of the BiOM affect walking biomechanics can inform prosthetic selection guidelines that are directly relevant to prosthetists and people with transtibial amputation. In addition, the BiOM includes a passive-elastic prosthetic foot with a manufacturer-recommended stiffness category based on a person’s bodyweight and activity level (Figure 1) (Össur, 2015). The effects of prosthetic stiffness categories and stance-phase power settings on affected to unaffected leg step-to-step transition work and effective foot length ratio may interact with one another. Ultimately, there may be a combination of prosthetic foot stiffness category and power setting that normalizes affected to unaffected leg step-to-step transition work and effective foot length ratio compared to non-amputees for a range of walking speeds.

We determined how prosthetic foot stiffness category and stance-phase power setting affect step-to-step transition work and effective foot length ratio of people with unilateral transtibial amputation walking on level ground at a range of speeds. We also determined if the effects of prosthetic foot stiffness category and stance-phase power setting interact with each other or with walking speed. We determined the effects of using a prosthesis with the manufacturer-recommended stiffness, one category less stiff than recommended (−1), and one category stiffer than recommended (+1) because they represent realistic stiffness categories that may be suggested for a given person. Furthermore, we determined the effects of using a prosthesis two categories less stiff than recommended (−2) because less stiff prostheses can store and return more energy than stiffer prostheses for the same compression, which may result in an increase in trailing affected leg work. We determined the effects of using the BiOM prosthesis with a recommended power setting based on a tuning session and two power settings that were greater than recommended (+10% and +20%) because a previous study suggested that power settings greater than typically-recommended may be beneficial (Ingraham et al., 2018). First, we hypothesized that a prosthetic foot stiffness category less stiff than recommended would increase affected trailing leg positive work, not change the magnitude of unaffected leading leg negative work, and decrease the prosthesis effective foot length ratio of people with unilateral transtibial amputation during walking at a range of speeds from 0.75–1.75 m/s. Second, we hypothesized that increasing the power setting in the BiOM would increase the affected trailing leg positive work, decrease the magnitude of unaffected leading leg negative work, and not affect the prosthesis effective foot length ratio of people with unilateral transtibial amputation during level ground walking at a range of speeds from 0.75–1.75 m/s. Third, we hypothesized the null hypothesis that the effects of prosthetic foot stiffness category and power setting would not interact with each other nor with walking speed at a range of speeds from 0.75–1.75 m/s. We also analyzed individual leg work during the unaffected to affected leg step-to-step transition to determine if people with unilateral transtibial amputation exhibit additional changes due to different prosthetic configurations.

## 2 Materials and methods

### 2.1 Participants

Thirteen people (10M, 3F, Table 1) with unilateral transtibial amputation (TTA) participated. All participants self-reported that they were at or above a K3 Medicare Functional Classification Level meaning that they were able to traverse most environmental barriers and could participate in activities beyond simple locomotion (Balk et al., 2018). Furthermore, all participants reported that they were free of neurological, cardiovascular, or musculoskeletal disease or injury other than that associated with amputation. All participants gave written informed consent prior to participating according to a protocol approved by the United States Department of Veteran Affairs’ Human Subjects Institutional Review Board (COMIRB #19–1052).

**TABLE 1:** Participant characteristics: sex, age, body mass including the passive-elastic prosthesis, recommended prosthetic stiffness category of the Low Profile (LP) Vari-flex prosthetic foot, prosthetic foot size, average axial stiffness of the heel of the recommended prosthesis without a shoe measured in Tacca et al. (Tacca et al., 2024), and average axial stiffness of the forefoot of the recommended prosthesis without a shoe measured in Tacca et al. (Tacca et al., 2024).

Participants	Sex	Age (years)	Mass (kg)	Recommended prosthetic stiffness category	Prosthetic size (cm)	Average Heel Stiffness (kN/m)	Average Forefoot Stiffness (kN/m)
1	M	32	59	3	25	45.02	37.24
2	F	35	60	3	26	43.35	35.61
3	F	49	64	3	25	45.02	37.24
4	F	24	67	3	25	45.02	37.24
5	M	38	67	4	25	49.66	41.08
6	M	50	73	4	28	44.65	36.19
7	M	46	74	4	26	47.99	39.45
8	M	34	79	5	28	49.29	40.03
9	M	47	81	5	25	54.30	44.92
10	M	50	86	5	29	47.62	38.40
11	M	38	88	6	27	55.60	45.50
12	M	49	98	6	27	55.60	45.50
13	M	47	110	7	28	58.57	47.71
Average		41.5	77.4				
S.D.		8.5	15.2				

### 2.2 Protocol

This study is a subset of a larger study that not only examined the effects of prosthetic foot stiffness category and power setting on step-to-step transition work and effective foot length ratio, but also examined the effects on biomechanical asymmetry, joint kinematics and kinetics, electromyography, metabolic power, and user satisfaction. Participants completed four laboratory sessions consisting of an acclimation and tuning session and three experimental sessions. Each of the three experimental sessions occurred on separate days that were at least 24 h apart. During the acclimation and tuning session, a certified prosthetist aligned participants with the BiOM powered prosthesis (BiOM T2, now Ottobock Empower, Duderstadt, Germany) that included a Low Profile (LP) Vari-Flex prosthetic foot (Össur, Reykjavik, Iceland) with a manufacturer recommended stiffness category and with the LP Vari-Flex prosthetic foot without the BiOM (Össur, 2015). Once the participant was aligned with the BiOM powered prosthesis, we placed reflective markers bilaterally on subjects’ legs and hips over joint centers and placed clusters of markers on the thigh and shank segments. For the BiOM, we placed reflective markers at the approximate locations of the first and fifth metatarsal heads, and posterior heel based on the locations for the unaffected leg. Malleoli markers were placed on the prosthetic ankle joint of the BiOM (Figure 1), which coincided with the center of rotation of the prosthesis in the sagittal plane. Participants then walked on a level dual-belt force measuring treadmill (Bertec, Columbus, OH, United States) at 1.25 m/s while we simultaneously measured marker trajectories at 200 Hz and ground reaction forces at 1000 Hz (Vicon Motion Systems, Centennial, CO, United States).

We iteratively tuned the BiOM using a tablet with the manufacturer-supplied application and 10 different tuning parameters (stiffness, power at fast cadence, power at slow cadence, power sensitivity, power timing–fast cadence, power timing–slow cadence, stiffness duration, stance damping, cadence range, and hardstop sensitivity) (iWalk Inc, 2013) until each participant’s prosthetic ankle range of motion, peak power, peak moment, and net mechanical work normalized to body mass including the prosthesis matched biological ankle values from non-amputees and the participant’s unaffected leg ankle values within two standard deviations of the mean (Jeffers et al., 2015; Montgomery and Grabowski, 2018). We digitized the reflective markers (Nexus, Vicon Motion Systems, Centennial, CO, United States) and calculated unaffected and affected leg stance-phase ankle range of motion, peak power, peak moment, and net mechanical work with a custom MATLAB (Mathworks Inc., Natick, MA, United States) script. We defined the foot using metatarsal markers and malleoli markers and the shank using malleoli and knee markers. We calculated the sagittal plane ankle range of motion as the angle between the foot and shank and calculated the sagittal plane peak ankle moment and power from inverse dynamics calculations. Our calculations only considered ankle angle, moment, and contributions to power in the sagittal plane, and these simplified calculations allowed us to iteratively tune the BiOM. We calculated net mechanical work as the integral of the ankle power with respect to time during a step. The resulting settings were used as the recommended BiOM settings in the subsequent sessions. The first session was approximately 3 h long.

During three experimental sessions, participants completed a series of trials for each of 16 different prosthetic configurations and used no more than six configurations per day. Participants walked using four different passive-elastic prosthetic foot stiffness categories including the manufacturer recommended stiffness category (Rec Cat), one category stiffer (+1 Cat), one category less stiff (−1 Cat), and two categories less stiff (−2 Cat) than recommended. Using each stiffness category of a passive-elastic prosthetic foot, subjects walked either without using the BiOM or using the BiOM powered ankle-foot prosthesis set at three different power settings that included the recommended power setting (Rec), and 10% greater (+10%), and 20% greater (+20%) than the recommended power setting. We chose to increase the power setting by +10% and +20% because pilot testing suggested that increasing the power setting by +10% and +20% was large enough to elicit a biomechanical response and the +20% power setting was close to the limits of the device capability when walking at 1.50 and 1.75 m/s. We randomized the order of configurations on each day by choosing a stiffness category (−2 Cat, −1 Cat, Rec Cat, +1 Cat) of the passive-elastic prosthetic foot. Then, we randomized the order of the four power conditions (passive/without the BiOM, Rec, +10%, +20%) for a given prosthetic foot stiffness category. Participants performed the same series of trials using each of the 16 prosthetic configurations. First, participants walked at 1.25 m/s for 5 min on a level dual-belt force measuring treadmill while we measured their metabolic rates. During minutes three and 4 of each trial, we simultaneously measured marker trajectories at 200 Hz and ground reaction forces at 1000 Hz for 30 s. Then, participants walked at 4 different speeds (0.75 m/s, 1.00 m/s, 1.50 m/s, and 1.75 m/s) on a level dual-belt force measuring treadmill for approximately 30 s per speed while we measured motion and ground reaction forces for 30 s.

### 2.3 Data collection

Prior to data collection, we placed reflective markers on the anterior superior iliac spines, posterior superior iliac spines, iliac crests, greater trochanters, lateral and medial femoral epicondyles, lateral and medial malleoli, first metatarsal heads, fifth metatarsal heads, and the posterior heels of each leg. For the BiOM and LP Vari-flex prostheses, we placed reflective markers at the approximate locations of the first and fifth metatarsal heads, and posterior heel based on the locations for the unaffected leg. For the BiOM, malleoli markers were placed on the prosthetic ankle joint center (Figure 1), which coincided with the center of rotation of the prosthesis in the sagittal plane. For the LP Vari-Flex prosthesis, malleoli markers were placed based on the approximate locations from the unaffected leg.

### 2.4 Data analysis

We digitized the reflective markers (Nexus, Vicon Motion Systems, Centennial, CO), filtered 3D marker positions with a fourth-order, low-pass Butterworth filter with a 7 Hz cut-off, (Visual3D, C-Motion, Boyds, MD, United States), and exported marker and force data. We used MATLAB (Mathworks Inc., Natick, MA, United States) to filter ground reaction forces with a fourth-order, low-pass Butterworth filter with a 30 Hz cut-off and then used a custom MATLAB script to determine ground contact using a 20 N vertical ground reaction force threshold for each leg. We calculated individual leg work during the affected to unaffected leg step-to-step transition and during the unaffected to affected leg step-to-step transition using the individual limbs method (Donelan et al., 2002). Step-to-step transitions were defined as periods of double support when both feet were in contact with the treadmill. We calculated center of mass velocity in the mediolateral (v_x, COM_), fore-aft (v_y,COM_), and vertical (v_z,COM_) directions during the step-to-step transitions by integrating acceleration with respect to time:

$$v_{x,COM}=\int \frac{F_{x,trail\;}+F_{x,lead}}{m}\;dt$$ (1)

$$v_{y,COM}=\int \frac{F_{y,trail\;}+F_{y,lead}}{m}\;dt$$ (2)

$$v_{z,COM}=\int \frac{\left(F_{z,trail\;}+F_{z,lead}\right)-mg}{m}\;dt$$ (3)

where *F*_*x,trail*_ and *F*_*x,lead*_ are the mediolateral, *F*_*y,trail*_ and *F*_*y,lead*_ are the fore-aft, and *F*_*z,trail*_ and *F*_*z,lead*_ are the vertical ground reaction forces for the trailing and leading legs, respectively, *m* is body mass including the prosthesis, *g* is the gravitational acceleration, and *dt* is the time differential (Eqs 1–3). To determine the integration constants when calculating center of mass velocity, we set the average mediolateral velocity at the beginning and end of a stride to be equal in magnitude and opposite in sign, the average fore-aft velocity over a stride to be equal to walking speed, and the average vertical velocity over a stride to be equal to zero. We calculated center of mass power from each leg (*P*_*trail*_ and *P*_*lead*_) as the dot product of the ground reaction force vector ( ${\vec{F}}_{trail}$ and ${\vec{F}}_{lead}$ ) and center of mass velocity vector ( ${\vec{v}}_{COM}$ ) (Eqs 4, 5):

$$P_{trail}={\vec{F}}_{trail}∙{\vec{v}}_{COM}$$ (4)

$$P_{lead}={\vec{F}}_{lead}∙{\vec{v}}_{COM}$$ (5)

Then, we used the trapezoidal method to integrate the center of mass power from each leg during the step-to-step transition to calculate individual leg work ( $W_{trail}$ and $W_{lead}$ ) (Eqs 6, 7):

$$W_{trail}=\int P_{trail}\;dt$$ (6)

$$W_{lead}=\int P_{lead}\;dt$$ (7)

We determined the center of pressure of each treadmill belt in Visual 3D (C-Motion, Boyds, MD, United States) using the treadmill ground reaction forces and moments. We filtered and exported marker trajectories to MATLAB where we used the lateral femoral epicondyles and malleoli to define the shank. We used a custom MATLAB script to determine the locations of the center of pressure of the foot with respect to the shank during the period from heel strike to the end of the single support phase. We calculated the effective foot length ratio (EFLR) as the ratio of the effective foot length and foot length (Eq. 8):

$$EFLR=\frac{effective\;foot\;length}{foot\;length}$$ (8)

where effective foot length is the horizontal distance from the heel to the most anterior portion of the roll-over shape and foot length is the horizontal distance from the heel to the end of the toe (length of the prosthesis or prosthesis size as in Hansen et al.) (Hansen et al., 2004). We estimated that the foot length of the biological foot was equal to the prosthesis size.

### 2.5 Statistical analysis

We constructed linear mixed effects models (Bates et al., 2015) to test for the effects of prosthetic foot stiffness category, stance-phase power setting, and walking speed on step-to-step transition work and effective foot length ratios. We constructed models with trailing affected leg work, leading unaffected leg work, trailing unaffected leg work, leading affected leg work and effective foot length ratio of both legs as the dependent variables. First, we constructed linear mixed effects models from trials where participants used the passive-elastic prosthetic feet to determine the effects of stiffness category. The fixed effects were stiffness category (categorical; −2, −1, Rec, +1) and speed (numerical; speed in m/s). We included the interaction between stiffness category and speed. For the models with effective foot length ratio as the dependent variable, there was an additional fixed effect of leg (categorical; AL, UL). We included the interactions between leg and stiffness category, and leg and speed. Second, we constructed linear mixed effects models from trials where participants used the recommended stiffness category prosthetic foot with and without the BiOM to determine the effects of power setting. The fixed effects were power setting (categorical; Passive, Rec, +10%, +20%) and speed (numerical; speed in m/s). We included the interaction between power setting and speed. For the models with effective foot length ratio as the dependent variable, there was an additional fixed effect of leg (categorical; AL, UL). We included the interactions between leg and power setting, and leg and speed. Third, we constructed linear mixed effects models from all trials to determine the interaction effect between stiffness category and power setting. The fixed effects were stiffness category (categorical; −2, −1, Rec, +1), power setting (categorical; Passive, Rec, +10%, +20%), and speed (numerical; speed in m/s). We included the interaction between stiffness category and power setting and set the participant as a random effect. For the models with effective foot length ratio as the dependent variable, there was an additional fixed effect of leg (categorical; AL, UL). We included the interactions between leg and stiffness category, and leg and power setting. For each model, we set the participant as a random effect to account for effects related to differences between participants such as body mass.

For each model, we used a significance level of *p* < 0.05 and removed all non-significant interactions. We report unstandardized model coefficients (B) for each significant association (dependent variable = B*independent variable + intercept). B represents the change in the dependent variable related to a unit change in the independent variable. All statistical tests were done in RStudio (Boston, MA, United States).

## 3 Results

### 3.1 Effect of prosthetic stiffness category on individual leg work

**FIGURE 2:**
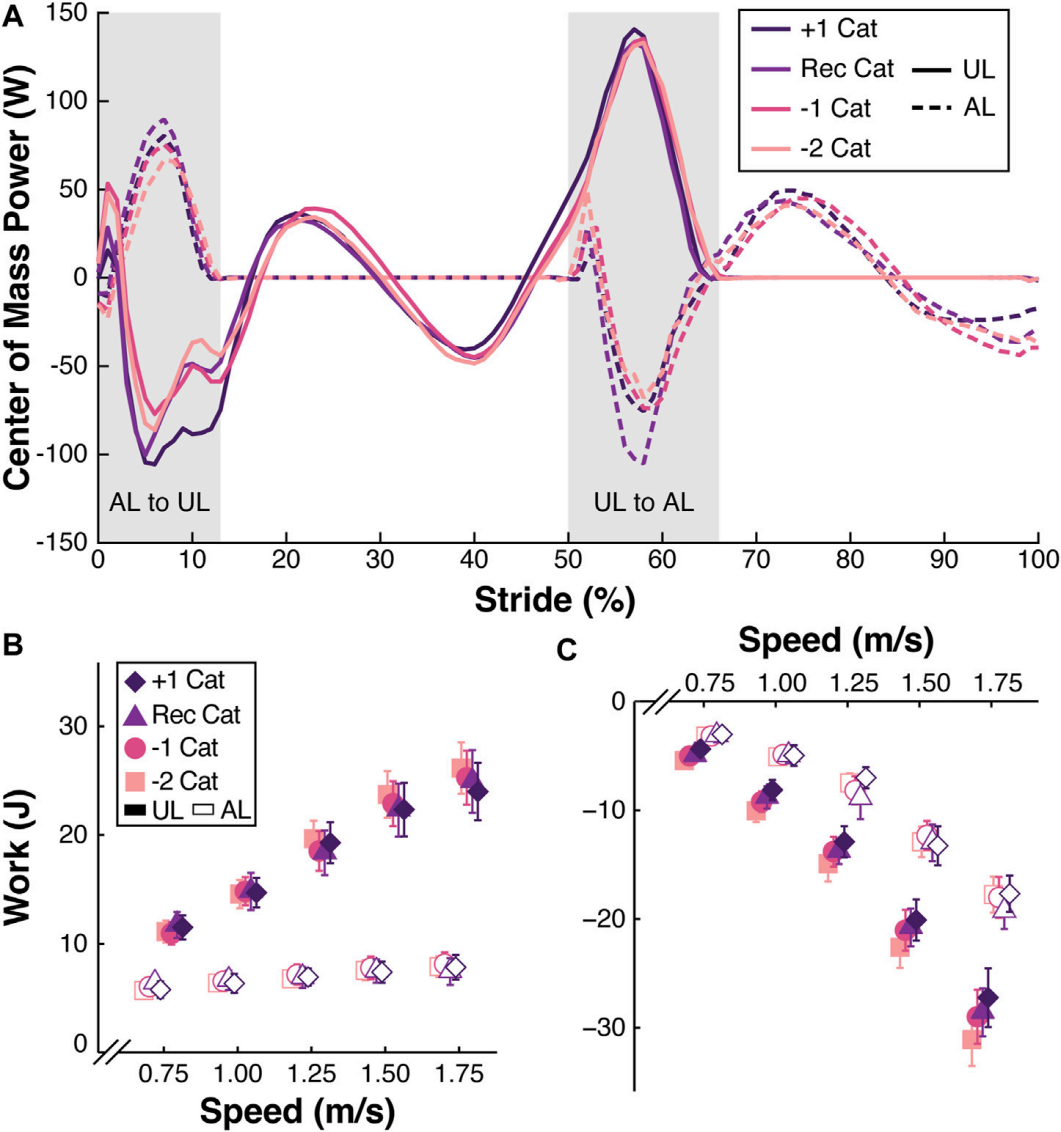
**(A)** Representative center of mass power (W) across a stride (%) from the unaffected leg (UL) heel strike to the subsequent UL heel strike for participant 1 walking at 1.25 m/s using a passive-elastic prosthesis with the +1, recommended (Rec), −1, and −2 passive-elastic prosthetic foot stiffness categories (Cat). Solid lines represent the UL and dashed lines represent the affected leg (AL). Gray shaded areas indicate the AL to UL and UL to AL step-to-step transitions. **(B)** Average positive work (J) done by the trailing AL and UL during the step-to-step transitions from all 13 participants walking at a range of speeds (m/s) using a passive-elastic prosthesis. **(C)** Average negative work (J) done by the leading UL and AL during the step-to-step transitions from all 13 participants walking at a range of speeds (m/s) using a passive-elastic prosthesis. Colors and symbols indicate different prosthetic foot stiffness categories (+1, Rec, −1, and −2). Filled symbols indicate the UL and open symbols indicate the AL. Error bars are standard error of the mean (SEM) and may be hidden behind the symbol. Symbols are offset for clarity.

When participants walked using a passive-elastic prosthesis, we did not detect a significant effect of stiffness category (*p* > 0.15; Table 2) or an interaction between stiffness category and walking speed on affected trailing leg positive work (AL_trail_ W_pos_) during the step-to-step transition (*p* > 0.05; Figure 2). However, when subjects walked using a passive-elastic prosthesis, AL_trail_ W_pos_ increased by 1.79 J for every 1 m/s faster walking speed (*p* < 0.0001; Figure 2; Table 2). When subjects walked using the recommended and +1 stiffness category prostheses without the BiOM, the magnitude of unaffected leading leg negative work (UL_lead_ W_neg_) decreased by 1.42 J and 2.25 J, respectively, compared to walking using the −2 stiffness category prosthesis (*p* < 0.03; Figure 2;, Table 2) during the step-to-step transition. We did not detect a difference in UL_lead_ W_neg_ between the −1 and −2 stiffness categories or an interaction between stiffness category and walking speed (*p* > 0.07; Figure 2;, Table 2). Furthermore, when subjects walked using a passive-elastic prosthesis, UL_lead_ W_neg_ increased in magnitude by 24.14 J for every 1 m/s faster walking speed (*p* < 0.0001; Figure 2; Table 2).

**TABLE 2:** Linear mixed model parameters for fixed effects of prosthetic stiffness categories and the interaction of prosthetic stiffness categories with speed on the positive work done by the affected leg (AL_trail_ W_pos_) and the negative work done by the unaffected leg (UL_lead_ W_neg_) during the step-to-step transition when participants used a passive-elastic prosthesis. Linear mixed models were simplified using backward elimination where non-significant (*p* > 0.05) interaction effects were removed. Coefficient estimates, 95% confidence intervals for coefficient estimates (CI), coefficient standard errors (SE), t values (t), and *p* values (p) are listed. For the prosthetic stiffness categories (categorical; −2, −1, Rec, +1), the model coefficients are in reference to the −2 category. The model coefficients for speed represent the change in the dependent variable for a 1 m/s increase in speed. Bold indicates a significant difference.

AL_trail_ W_pos_ (J)	Estimate (B)	*CI*	*SE*	*t*	*p*
Intercept	4.59	[2.80, 6.38]	0.89	5.16	**0.0001**
Stiffness Category [-1]	0.27	[−0.09, 0.64]	0.19	1.46	0.147
Stiffness Category [Rec]	0.11	[−0.25, 0.48]	0.19	0.59	0.553
Stiffness Category [+1]	−0.00	[−0.37, 0.37]	0.19	−0.01	0.991
Speed [m/s]	1.79	[1.42, 2.17]	0.19	9.42	**< 0.0001**

UL_lead_ W_neg_ (J)	Estimate (B)	*CI*	*SE*	*t*	*p*
Intercept	13.34	[10.36, 16.33]	1.52	8.79	**< 0.0001**
Stiffness Category [-1]	1.20	[−0.07, 2.46]	0.65	1.85	0.065
Stiffness Category [Rec]	1.42	[0.16, 2.68]	0.65	2.20	**0.029**
Stiffness Category [+1]	2.25	[0.99, 3.51]	0.65	3.48	**0.0006**
Speed [m/s]	−24.14	[−25.41, −22.86]	0.65	−36.94	**< 0.0001**

When participants walked using a passive-elastic prosthesis, we did not detect a significant effect of stiffness category (*p* > 0.17; Table 3) or an interaction between stiffness category and walking speed on unaffected trailing leg positive work (UL_trail_ W_pos_) during the step-to-step transition (*p* > 0.06; Figure 2). However, when subjects walked using a passive-elastic prosthesis, UL_trail_ W_pos_ increased by 13.94 J for every 1 m/s faster walking speed (*p* < 0.0001; Figure 2; Table 3). Similarly, when subjects walked using a passive-elastic prosthesis, we did not detect a significant effect of stiffness category (*p* > 0.29; Table 3) or an interaction between stiffness category and walking speed on the affected leading leg negative work (AL_lead_ W_neg_) during the step-to-step transition (*p* > 0.39; Figure 2). When subjects walked using a passive-elastic prosthesis, AL_lead_ W_neg_ increased in magnitude by 15.29 J for every 1 m/s faster walking speed (*p* < 0.0001; Figure 2; Table 3).

**TABLE 3:** Linear mixed model parameters for fixed effects of prosthetic stiffness categories and the interaction of prosthetic stiffness categories with speed on the positive work done by the unaffected leg (UL_trail_ W_pos_) and the negative work done by the affected leg (AL_lead_ W_neg_) during the step-to-step transition when participants used a passive-elastic prosthesis. Linear mixed models were simplified using backward elimination where non-significant (*p* > 0.05) interaction effects were removed. Coefficient estimates, 95% confidence intervals for coefficient estimates (CI), coefficient standard errors (SE), t values (t), and *p* values (p) are listed. For the prosthetic stiffness categories (categorical; −2, −1, Rec, +1), the model coefficients are in reference to the −2 category. The model coefficients for speed represent the change in the dependent variable for a 1 m/s increase in speed. Bold indicates a significant difference.

UL_trail_ W_pos_ (J)	Estimate (B)	*CI*	*SE*	*t*	*p*
Intercept	1.53	[−2.20, 5.27]	1.86	0.82	0.423
Stiffness Category [-1]	−0.55	[−1.49, 0.40]	0.48	−1.13	0.259
Stiffness Category [Rec]	−0.61	[−1.56, 0.33]	0.48	−1.27	0.207
Stiffness Category [+1]	−0.66	[−1.60, 0.28]	0.48	−1.36	0.174
Speed [m/s]	13.94	[12.99,14.90]	0.49	28.48	**< 0.0001**

AL_lead_ W_neg_ (J)	Estimate (B)	*CI*	*SE*	*t*	*p*
Intercept	9.80	[7.40, 12.20]	1.22	8.03	**< 0.0001**
Stiffness Category [-1]	−0.02	[−1.06, 1.02]	0.53	−0.05	0.964
Stiffness Category [Rec]	−0.57	[−1.61, 0.47]	0.53	−1.07	0.287
Stiffness Category [+1]	0.10	[−0.94, 1.14]	0.53	0.18	0.858
Speed [m/s]	−15.29	[−16.34, −14.24]	0.54	−28.36	**< 0.0001**

### 3.2 Effect of prosthetic power setting on individual leg work

**FIGURE 3:**
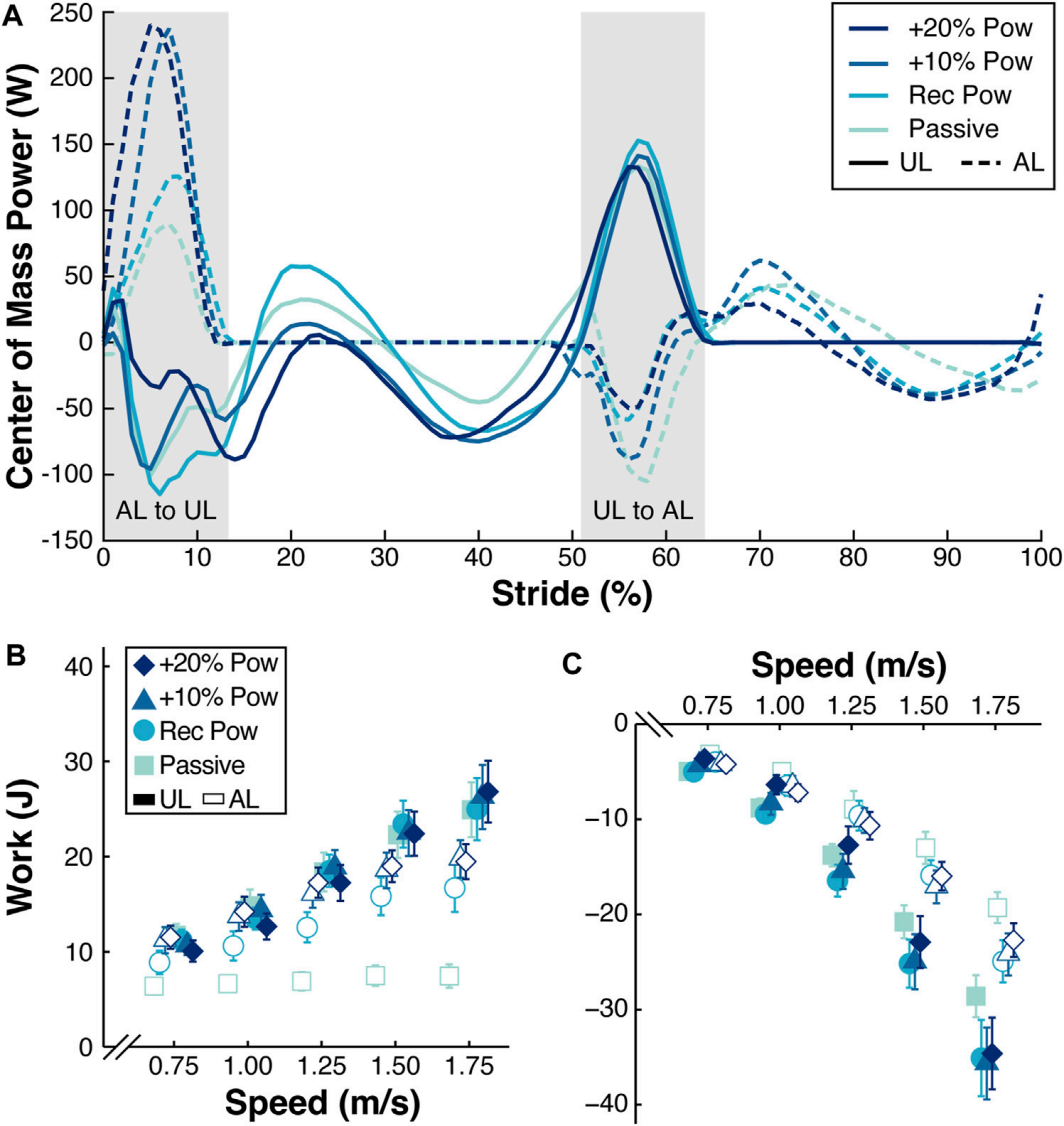
**(A)** Representative curves of the center of mass power (W) across a stride (%) from the unaffected leg (UL) heel strike to the subsequent UL heel strike for participant 1 walking at 1.25 m/s using the recommended (Rec) category (Cat) prosthesis without the BiOM (Passive) and attached to the BiOM at Rec, +10%, and +20% power settings. Solid lines represent the UL and dashed lines represent the affected leg (AL). Gray areas indicate the AL to UL and UL to AL step-to-step transitions. **(B)** Average work (J) done by the trailing AL and UL during the step-to-step transitions from all 13 participants walking at a range of speeds (m/s) using the Rec Cat without the BiOM (Passive) and attached to the BiOM at Rec, +10%, and +20% power settings (colors and symbols). **(C)** Average work (J) done by the leading UL and AL during the step-to-step transitions from all 13 participants walking at a range of speeds (m/s) with the Rec Cat without the BiOM (Passive) and attached to the BiOM at Rec, +10%, and +20% power settings (colors and symbols). Filled symbols indicate the UL and open symbols indicate the AL. Error bars are standard error of the mean (SEM) and may be hidden behind the symbol. Symbols are offset for clarity.

When participants walked using the recommended stiffness category prosthesis with and without the BiOM, there was a significant effect of power setting on AL_trail_ W_pos_ that depended on walking speed (*p* < 0.0001; Figure 3; Table 4) during the step-to-step transition. At 0.75 m/s, when participants walked using the BiOM at recommended, +10%, and +20% power settings, they increased AL_trail_ W_pos_ by 2.37 J, 5.09 J, and 5.77 J, respectively, compared to the passive-elastic prosthesis (Figure 3; Table 4). The differences in AL_trail_ W_pos_ between using the BiOM and the passive-elastic prosthesis increased with faster walking speeds so that at 1.75 m/s use of the BiOM at recommended, +10%, and +20% power settings increased AL_trail_ W_pos_ by 9.48 J, 12.63 J, and 12.87 J, respectively, compared to the passive-elastic prosthesis (Figure 3; Table 4). When participants walked using the recommended category passive-elastic prosthesis with and without the BiOM, there was a significant effect of power setting on UL_lead_ W_neg_ that depended on walking speed (*p* < 0.01; Figure 3; Table 4). At 0.75 m/s, when participants walked using the BiOM at recommended, +10%, and +20% power settings, they decreased the magnitude of UL_lead_ W_neg_ by 0.41 J, 1.53 J, and 3.09 J, respectively, compared to the passive-elastic prosthesis (Figure 3; Table 4). However, the effect of power setting on UL_lead_ W_neg_ changed with speed so that at 1.75 m/s use of the BiOM at recommended, +10%, and +20% power settings increased the magnitude of UL_lead_ W_neg_ by 5.92 J, 6.08 J, and 4.14 J, respectively, compared to the passive-elastic prosthesis (Figure 3; Table 4).

**TABLE 4:** Linear mixed model parameters for the interaction of the BiOM prosthetic power settings with speed on the positive work done by the affected leg (AL_trail_ W_pos_) and the negative work done by the unaffected leg (UL_lead_ W_neg_) during the step-to-step transition when participants used the recommended stiffness category prosthesis with and without the BiOM. Linear mixed models were simplified using backward elimination where non-significant (*p* > 0.05) interaction effects were removed. Coefficient estimates, 95% confidence intervals for coefficient estimates (CI), coefficient standard errors (SE), t values (t), and *p* values (p) are listed. For the power settings (categorical; Passive, Rec, +10%, +20%), the model coefficients are in reference to the passive-elastic prosthesis. The model coefficients for speed represent the change in the dependent variable for a 1 m/s increase in speed. Bold indicates a significant difference.

AL_trail_ W_pos_ (J)	Estimate (B)	*CI*	*SE*	*t*	*p*
Intercept	5.75	[2.36, 9.14]	1.73	3.32	**0.002**
Power Setting [Rec]	−2.96	[−6.00, 0.09]	1.57	−1.88	0.061
Power Setting [+10%]	−0.57	[−3.61, 2.47]	1.57	−0.36	0.717
Power Setting [+20%]	0.44	[−2.61, 3.48]	1.57	0.28	0.780
Speed [m/s]	0.92	[−0.74, 2.58]	0.86	1.08	0.282
Power Setting [Rec] * Speed [m/s]	7.11	[4.74, 9.47]	1.22	5.82	**< 0.0001**
Power Setting [+10%] * Speed [m/s]	7.54	[5.18, 9.91]	1.22	6.18	**< 0.0001**
Power Setting [+20%] * Speed [m/s]	7.10	[4.74, 9.47]	1.22	5.82	**< 0.0001**

UL_lead_ W_neg_ (J)	Estimate (B)	*CI*	*SE*	*t*	*p*
Intercept	14.46	[9.18, 19.74]	2.73	5.18	**< 0.0001**
Power Setting [Rec]	5.16	[−1.07, 11.40]	3.22	1.61	0.110
Power Setting [+10%]	7.24	[1.00, 13.47]	3.22	2.25	**0.025**
Power Setting [+20%]	8.51	[2.28, 14.75]	3.22	2.65	**0.009**
Speed [m/s]	−23.92	[−27.32, −20.52]	1.75	−13.65	**< 0.0001**
Power Setting [Rec] * Speed [m/s]	−6.33	[−11.18, −1.48]	2.50	−2.53	**0.012**
Power Setting [+10%] * Speed [m/s]	−7.61	[−12.46, −2.77]	2.50	−3.05	**0.003**
Power Setting [+20%] * Speed [m/s]	−7.23	[−12.08, −2.39]	2.50	−2.89	**0.004**

When participants walked using the recommended category passive-elastic prosthesis with and without the BiOM, we did not detect a significant difference in UL_trail_ W_pos_ when participants used the BiOM at recommended and +10% power settings compared to the passive-elastic prosthesis (*p* > 0.19; Figure 3;, Table 5) during the step-to-step transition. However, there was a significant difference between use of the BiOM at the +20% power setting compared to the passive-elastic prosthesis that depended on walking speed (*p* < 0.02; Figure 3;, Table 5). At 0.75 m/s, use of the BiOM at the +20% power setting decreased UL_trail_ W_pos_ by 2.43 J compared to the passive-elastic prosthesis (Figure 3; Table 5). The effect of the +20% power setting on UL_trail_ W_pos_ changed with speed so that at 1.75 m/s use of the BiOM at the +20% power setting increased UL_trail_ W_pos_ by 1.17 J compared to the passive-elastic prosthesis (Figure 3; Table 5). Furthermore, we did not detect a significant difference in AL_lead_ W_neg_ between use of the BiOM at the +20% power setting compared to the passive-elastic prosthesis (*p* = 0.28; Figure 3;, Table 5). However, there was a significant difference in AL_lead_ W_neg_ between use of the BiOM at recommended and +10% power settings compared to the passive-elastic prosthesis, and this difference was greater with faster walking speeds (*p* < 0.03; Figure 3;, Table 5). At 0.75 m/s, use of the BiOM at the recommended and +10% power settings increased the magnitude of AL_lead_ W_neg_ by 0.21 J and 0.63 J, respectively, compared to the passive-elastic prosthesis (Figure 3; Table 5). The effect of the recommended and +10% power settings on AL_lead_ W_neg_ changed with speed so that at 1.75 m/s use of the BiOM at the recommended and +10% power settings increased the magnitude of AL_lead_ W_neg_ by 4.16 J and 4.34 J, respectively, compared to the passive-elastic prosthesis (Figure 3; Table 5).

**TABLE 5:** Linear mixed model parameters for the interaction of the BiOM prosthetic power settings with speed on the positive work done by the unaffected leg (UL_trail_ W_pos_) and the negative work done by the affected leg (AL_lead_ W_neg_) during the step-to-step transition when participants used the recommended stiffness category prosthesis with and without the BiOM. Linear mixed models were simplified using backward elimination where non-significant (*p* > 0.05) interaction effects were removed. Coefficient estimates, 95% confidence intervals for coefficient estimates (CI), coefficient standard errors (SE), t values (t), and *p* values (p) are listed. For the power settings (categorical; Passive, Rec, +10%, +20%), the model coefficients are in reference to the passive-elastic prosthesis. The model coefficients for speed represent the change in the dependent variable for a 1 m/s increase in speed. Bold indicates a significant difference.

UL_trail_ W_pos_ (J)	Estimate (B)	*CI*	*SE*	*t*	*p*
Intercept	1.71	[−2.83, 6.25]	2.31	0.74	0.466
Power Setting [Rec]	−1.91	[−5.79, 1.97]	2.00	−0.95	0.341
Power Setting [+10%]	−2.62	[−6.50, 1.26]	2.00	−1.31	0.191
Power Setting [+20%]	−5.13	[−9.01, −1.25]	2.00	−2.57	**0.011**
Speed [m/s]	13.33	[11.21, 15.44]	1.09	12.21	**< 0.0001**
Power Setting [Rec] * Speed [m/s]	1.44	[−1.58, 4.45]	1.56	0.92	0.356
Power Setting [+10%] * Speed [m/s]	2.13	[−0.88, 5.15]	1.56	1.37	0.172
Power Setting [+20%] * Speed [m/s]	3.60	[0.59, 6.62]	1.56	2.32	**0.022**

AL_lead_ W_neg_ (J)	Estimate (B)	*CI*	*SE*	*t*	*p*
Intercept	10.32	[6.67, 13.96]	1.88	5.48	**< 0.0001**
Power Setting [Rec]	2.75	[−1.50, 7.01]	2.19	1.26	0.211
Power Setting [+10%]	2.15	[−2.10, 6.41]	2.19	0.98	0.328
Power Setting [+20%]	0.12	[−4.14, 4.37]	2.19	0.05	0.957
Speed [m/s]	−16.16	[−18.48, −13.84]	1.20	−13.50	**< 0.0001**
Power Setting [Rec] * Speed [m/s]	−3.95	[−7.26, −0.64]	1.71	−2.31	**0.022**
Power Setting [+10%] * Speed [m/s]	−3.71	[−7.01, −0.40]	1.71	−2.17	**0.031**
Power Setting [+20%] * Speed [m/s]	−1.86	[−5.17, 1.45]	1.71	−1.09	0.278

### 3.3 Interaction between prosthetic stiffness category and power setting on individual leg work

We analyzed trials from the combinations of prosthetic stiffness categories and power settings to determine if their effects on individual leg work depended on each other. During the AL to UL step-to-step transition, the effect of power setting on AL_trail_ W_pos_ was greater when the BiOM was attached to some of the prosthetic stiffness categories compared to the other stiffness categories. The effect of using the BiOM at the +10% power setting increased AL_trail_ W_pos_ by 1.52 J when the BiOM was attached to the recommended compared to the −2 stiffness category prosthesis (*p* = 0.03; Supplementary Table S1, Supplementary Figure S1). Furthermore, the effects of using the BiOM at recommended, +10%, and +20% power settings increased AL_trail_ W_pos_ by 2.03 J, 2.54 J, and 1.74 J, respectively, when the BiOM was attached to the +1 compared to the −2 stiffness category prosthesis (*p* < 0.01; Supplementary Table S1, Supplementary Figure S1). We did not detect a significant interaction between the effects of stiffness categories and power settings on UL_lead_ W_neg_ (*p* > 0.32; Supplementary Table S1, Supplementary Figure S1).

We did not detect a significant interaction between the effects of prosthetic stiffness categories and power settings on UL_trail_ W_pos_ during the UL to AL step-to-step transition (*p* > 0.07; Supplementary Table S2, Supplementary Figure S2. The difference in AL_lead_ W_neg_ between use of the BiOM at the +10% power setting and the passive-elastic prostheses was attenuated by 1.78 J when the BiOM was attached to the −1 compared to the −2 stiffness category prosthesis (*p* = 0.04; Supplementary Table S2, Supplementary Figure S2). We did not detect any other significant interactions between the effects of stiffness category and power setting on individual leg work.

### 3.4 Effective foot length ratio

**FIGURE 4:**
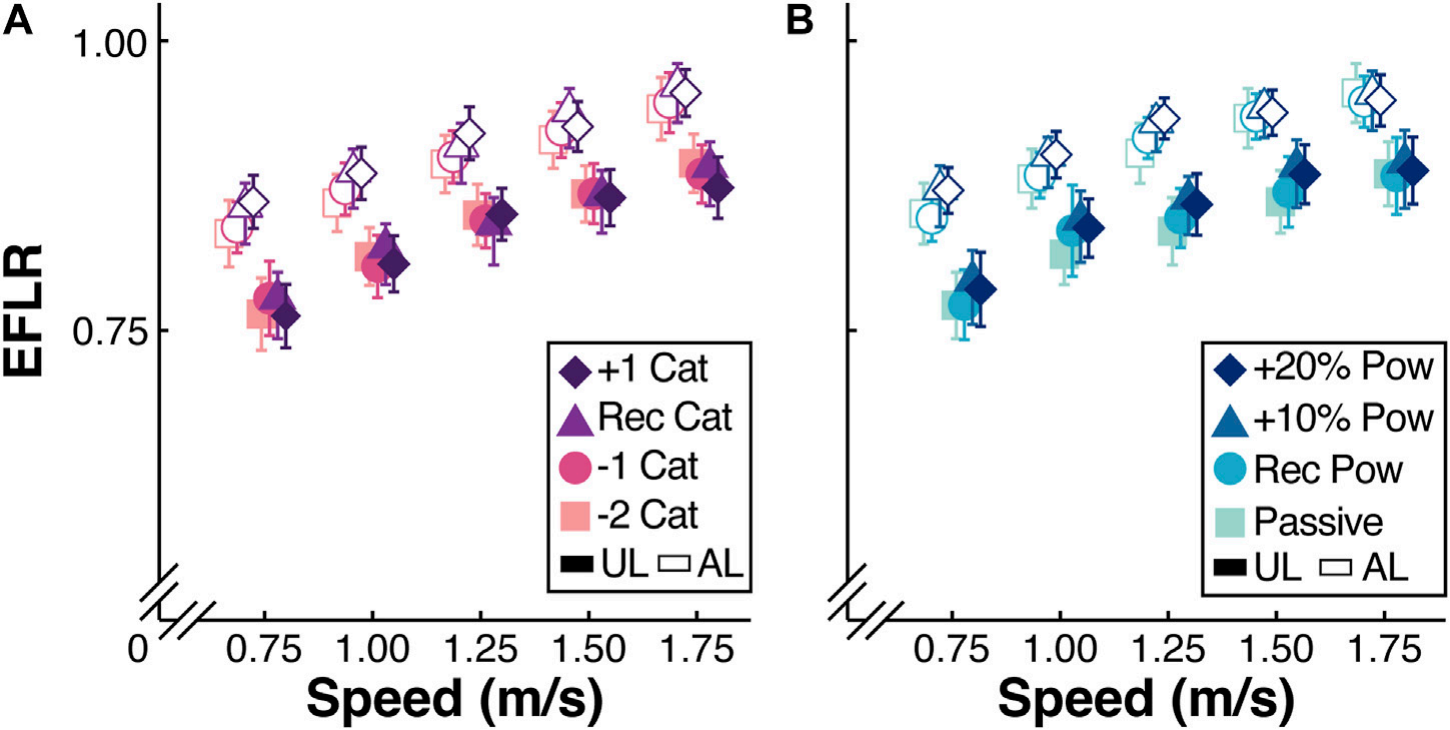
**(A)** Average effective foot length ratio (EFLR) of the unaffected leg (UL) and affected leg (AL) from all 13 participants walking at a range of speeds (m/s) using a passive-elastic prosthesis. Colors and symbols indicate use of a passive-elastic prosthesis with the +1, recommended (Rec), −1, and −2 passive-elastic prosthetic foot stiffness categories (Cat). **(B)** Average EFLR of the AL and UL from all 13 participants walking at a range of speeds (m/s) using the Rec Cat without the BiOM (Passive) and attached to the BiOM at Rec, +10%, and +20% power settings (colors and symbols). Filled symbols indicate the UL and open symbols indicate the AL. Error bars are standard error of the mean (SEM) and may be hidden behind the symbol. Symbols are offset for clarity.

When participants walked using the passive-elastic prostheses, the effective foot length ratio (EFLR) increased by 0.11 for every 1 m/s faster walking speed (*p* < 0.0001; Table 6;, Figure 4) and the prosthetic foot had a 0.05 greater EFLR compared to the biological foot (*p* < 0.0001; Table 6;, Figure 4). We did not detect a difference in the EFLR of the prosthesis between the −1 or recommended stiffness categories and the −2 stiffness category prosthesis (*p* > 0.08; Table 6;, Figure 4). However, we found that use of the +1 stiffness category prosthesis increased the EFLR of the prosthesis by 0.02 compared to the −2 stiffness category (*p* = 0.02; Table 6;, Figure 4) and this effect did not depend on walking speed (*p* > 0.40).

**TABLE 6:** Linear mixed model parameters for the fixed effects of prosthetic stiffness category, leg type, speed, and the interactions of stiffness category with speed, leg type with speed, and stiffness category with leg type on the effective foot length ratio (EFLR) when participants used the passive-elastic prostheses during walking. Linear mixed models were simplified using backward elimination where non-significant (*p* > 0.05) interaction effects were removed. Coefficient estimates, 95% confidence intervals for coefficient estimates (CI), coefficient standard errors (SE), t values (t), and *p* values (p) are listed. For the prosthetic stiffness categories (categorical; −2, −1, Rec, +1), the model coefficients are in reference to the −2 category. The model coefficients for leg (categorical; AL, UL) are in reference to the AL. The model coefficients for speed represent the change in the dependent variable for a 1 m/s increase in speed. Bold indicates a significant difference.

EFLR	Estimate (B)	*CI*	*SE*	*t*	*p*
Intercept	0.75	[0.70, 0.80]	0.02	32.44	**< 0.0001**
Stiffness Category [-1]	0.01	[−0.01, 0.02]	0.01	0.83	0.408
Stiffness Category [Rec]	0.02	[−0.00, 0.03]	0.01	1.78	0.076
Stiffness Category [+1]	0.02	[0.00, 0.04]	0.01	2.34	**0.020**
Leg [UL]	−0.05	[−0.07, −0.03]	0.01	−5.67	**< 0.0001**
Speed [m/s]	0.11	[0.10, 0.12]	0.01	17.46	**< 0.0001**
Stiffness Category [-1]* Leg [UL]	−0.01	[−0.03, 0.02]	0.01	−0.72	0.470
Stiffness Category [Rec]* Leg [UL]	−0.02	[−0.04, 0.00]	0.01	−1.58	0.115
Stiffness Category [+1]* Leg [UL]	−0.03	[−0.05, −0.00]	0.01	−2.13	**0.033**

When participants walked using the recommended stiffness category with and without the BiOM, the EFLR of both the prosthesis and biological foot increased by 0.11 for every 1 m/s faster speed (*p* < 0.0001; Table 7;, Figure 4). When participants walked using the BiOM at +10% and +20% power settings, the EFLR of both the prosthetic and biological foot increased by 0.02 compared to when the participants walked using the passive-elastic prosthesis (*p* < 0.01; Table 7;, Figure 4), and this effect did not depend on walking speed (*p* > 0.54).

**TABLE 7:** Linear mixed model parameters for the fixed effects of prosthetic power setting, leg type, speed, and the interactions of power setting with speed, leg type with speed, and power setting with leg type on the effective foot length ratio (EFLR) when participants used the recommended stiffness category with and without the BiOM during walking. Linear mixed models were simplified using backward elimination where non-significant (*p* > 0.05) interaction effects were removed. Coefficient estimates, 95% confidence intervals for coefficient estimates (CI), coefficient standard errors (SE), t values (t), and *p* values (p) are listed. For the prosthetic power setting (categorical; Passive, Rec, +10%, +20%), the model coefficients are in reference to the passive-elastic prosthesis. The model coefficients for leg type (categorical; AL, UL) are in reference to the AL. The model coefficients for speed represent the change in the dependent variable for a 1 m/s increase in speed. Bold indicates a significant difference.

EFLR	Estimate (B)	*CI*	*SE*	*t*	*p*
Intercept	0.77	[0.73, 0.82]	0.02	32.80	**< 0.0001**
Power Setting [Rec]	0.01	[−0.01, 0.02]	0.01	1.02	0.306
Power Setting [+10%]	0.02	[0.00, 0.03]	0.01	2.56	**0.011**
Power Setting [+20%]	0.02	[0.01, 0.03]	0.01	2.90	**0.004**
Leg [UL]	−0.07	[−0.07, −0.06]	0.00	−15.52	**< 0.0001**
Speed [m/s]	0.10	[0.09, 0.12]	0.01	16.94	**< 0.0001**

### 3.5 Interaction between prosthetic stiffness category and power setting on effective foot length ratio

We analyzed trials from the combinations of prosthetic stiffness categories and power settings to determine if their effects on EFLR depended on each other. We did not detect a significant difference in AL ELFR between use of the BiOM at any of the power settings and use of a passive-elastic prosthesis (*p* > 0.18; Supplementary Table S3, Supplementary Figure S3). However, the AL EFLR of the BiOM at the recommended power setting was 0.02 greater than the AL EFLR of the passive-elastic prosthesis when the BiOM was attached to the +1 stiffness category, but there was no difference in AL EFLR of the BiOM at the recommended power setting and a passive-elastic prosthesis when the BiOM was attached to the −2 stiffness category (*p* = 0.02; Supplementary Table S3, Supplementary Figure S3). We did not detect any other significant interactions between the effects of stiffness category and power setting on EFLR (*p* > 0.05, Supplementary Table S3, Supplementary Figure S3).

## 4 Discussion

In contrast with our first hypothesis, we found that there was no significant effect of prosthetic stiffness category on positive affected trailing leg work during the step-to-step transition at a range of walking speeds. Our results contrast with those from a previous study that found that use of a less stiff experimental prosthetic foot increased affected trailing leg positive work during the step-to-step transition compared to use of a stiffer prosthesis at 0.7–1.5 m/s (Adamczyk et al., 2017). The average difference in forefoot stiffness in the stiffest and least stiff experimental prosthetic foot tested in the previous study was 24.6 kN/m (average forefoot stiffness: least stiff– 35.7 kN/m; stiffest– 60.3 kN/m) (Adamczyk et al., 2017), whereas the difference in forefoot stiffness between the stiffest and least stiff prosthesis that we tested was on average 12.0 kN/m (average forefoot stiffness: least stiff– 31.9 kN/m; stiffest– 43.9 kN/m) (Tacca et al., 2024) for each participant (Supplementary Material). It is possible that over a wider range of prosthetic stiffness categories, there could be an effect of stiffness on positive affected trailing leg work. However, the range of prosthetic stiffness categories that we tested (−2, −1, Rec, +1) represent typical categories that a prosthetist may use when selecting a prosthesis and our results suggest that passive-elastic prosthetic foot stiffness category does not significantly change positive affected trailing leg work.

In contrast to our first hypothesis, participants walked with a lower magnitude of negative unaffected leading leg work using the recommended and +1 compared to the −2 stiffness category passive-elastic prosthesis. This result may be related to the effective foot length ratio of each prosthesis. We found that the AL effective foot length ratio was greater for the +1 compared to the −2 stiffness category passive-elastic prosthesis. A greater effective foot length ratio can reduce the required directional change of the center-of-mass velocity during the step-to-step transition and may allow a reduction in the magnitude of negative individual leading leg work during walking (Adamczyk et al., 2006). Overall, our results suggest that people with unilateral transtibial amputation can benefit from using stiffer rather than less stiff passive-elastic prosthetic feet because stiffer passive-elastic prostheses may reduce the magnitude of negative unaffected leading leg work during the step-to-step transition and thus presumably reduce the risk of knee osteoarthritis in the unaffected leg (Morgenroth et al., 2011).

In support of our first hypothesis, we found that there was a greater effective foot length ratio for the +1 than the −2 stiffness category passive-elastic prosthetic foot. Our results are in line with a previous study that found an increase in effective foot length ratio with increased passive-elastic prosthetic foot stiffness categories (Halsne et al., 2020). A previous study found that commercially available passive and passive-elastic prosthetic feet have effective foot length ratios that range from 0.63 to 0.81 compared to 0.83 for biological feet (Hansen et al., 2004). In agreement with Hansen et al. (Hansen et al., 2004), we found that the average effective foot length ratio for biological feet was 0.83; however, we found that the average effective foot length ratio for the LP Vari-flex passive-elastic prosthetic feet was 0.89 (Figure 4). Our results suggest that LP Vari-flex prostheses may have greater effective foot length ratios than other commercially available prosthetic feet and that increasing the stiffness category can increase the effective foot length ratio, which may reduce the magnitude of negative work done by the unaffected leading leg during the step-to-step transition.

In support of our second hypothesis, we found that increasing the BiOM power setting increased positive affected trailing leg work during the step-to-step transition at a range of walking speeds. Our results suggest that prosthetists should tune the BiOM prosthesis with a power setting greater than typically recommended to increase the positive work done by the affected trailing leg to be at a similar value to that of the unaffected leg. For example, when participants walked using the BiOM at the recommended power setting, the average positive affected trailing leg work ranged from 8.5 J to 16.0 J during the step-to-step transition when walking at 0.75 m/s to 1.75 m/s (Figure 3). When the BiOM was set to the +20% power setting, the positive affected trailing leg work ranged from 10.9 J to 18.8 J at 0.75 m/s to 1.75 m/s (Figure 3). When participants used the BiOM at the +20% power setting compared to the recommended setting, their positive affected trailing leg work better matched the average positive unaffected trailing leg work, which ranged from 10.5 J to 24.7 J at 0.75 m/s to 1.75 m/s (Figure 3). In addition, the effect of power setting on positive affected trailing leg work depended on walking speed, so that the difference between use of the BiOM and the passive-elastic prosthesis was greater at faster compared to slower walking speeds. This suggests that use of the BiOM may improve affected trailing leg work more at faster compared to slower walking speeds *versus* a passive-elastic prosthesis.

In partial support of our second hypothesis, increasing the BiOM power setting decreased the magnitude of negative unaffected leading leg work during the step-to-step transition when walking at 0.75–1.00 m/s but not at 1.25–1.75 m/s. When participants walked at 0.75–1.00 m/s, use of the BiOM at the +20% power setting resulted in the lowest magnitude of negative unaffected leading leg work compared to the other power settings and the passive-elastic prosthesis. This suggests that use of the BiOM at a power setting 20% greater than typically recommended decreases the magnitude of negative unaffected leading leg work during walking at 0.75–1.00 m/s, which could reduce the risk of joint pain and osteoarthritis in the unaffected leg. However, when participants walked using the BiOM at 1.50–1.75 m/s, use of the BiOM at the +20% power setting increased the magnitude of negative unaffected leading leg work by 2.3–4.1 J compared to use of the passive-elastic prosthesis. It is possible that when participants walked at 1.50–1.75 m/s using the BiOM compared to a passive-elastic prosthesis, instability, measured as the rate of change of whole body angular momentum, and step width increased (Neptune and Vistamehr, 2019). Increasing step width beyond the steady-state step width can increase individual leg work during step-to-step transitions (Kuo et al., 2005). Perhaps powered prosthetic designs that are better able to regulate whole body angular momentum at 1.50–1.75 m/s could help people with unilateral transtibial amputation to decrease the magnitude of negative unaffected leading leg work compared to use of passive-elastic prostheses. In addition, during the acclimation session in our study, participants walked on the treadmill at 1.25 m/s and we tuned the BiOM to match biological ankle values within 2 SD of the mean. Perhaps longer acclimation time at speeds greater than 1.25 m/s and/or tuning the BiOM for a range of speeds could help people with unilateral transtibial amputation to decrease the magnitude of negative unaffected leading leg work when using the BiOM to walk at 1.50–1.75 m/s.

In contrast to our second hypothesis, we found that increasing the BiOM power setting increased the effective foot length ratio of the prosthesis at a range of walking speeds. The effect of power setting on the effective foot length ratio of the prosthesis was similar to the effect of stiffness category on the effective foot length ratio of the prosthesis. The average effective foot length ratio was 0.02 greater using the BiOM at the +20% power setting compared to the passive-elastic prosthesis (Figure 4). For a size 26 cm prosthesis (median prosthesis size used by the participants in this study), a 0.02 increase in effective foot length ratio equates to a 0.5 cm increase in effective foot length. Therefore, it is unclear if the effects of stiffness category and power setting on effective foot length ratio that were observed in this study are clinically meaningful. Nevertheless, prosthetists may consider that increasing prosthetic stiffness category and power setting can increase the effective foot length ratio when selecting a prosthesis, which in turn could reduce the magnitude of unaffected leading leg work.

We observed some interactions between the effects of prosthetic foot stiffness category and power setting on step-to-step transition work and effective foot length ratio. Most notably, use of the BiOM attached to the +1 stiffness category prosthetic foot increased affected trailing leg positive work compared to when the BiOM was attached to the −2 stiffness category prosthetic foot. This may suggest that prosthetists and people with a transtibial amputation should attach a stiffer than recommended category prosthetic foot beneath the BiOM to increase affected trailing leg positive work. Herr and Grabowski found that use of a prototype of the BiOM at recommended power settings during walking at 0.75–1.75 m/s increased affected trailing leg positive work compared to use of a passive-elastic prosthesis and resulted in an 8% decrease in the metabolic cost of walking (Herr and Grabowski, 2012). Perhaps a combination of power setting and stiffness category that increases affected leg trailing positive work to greater values than in Herr and Grabowski (Herr and Grabowski, 2012) could result in further metabolic cost reductions. Future studies should examine the effects of different prosthetic stiffness and power configurations on the metabolic cost of walking.

Our study had some potential limitations. For each prosthetic configuration, participants walked for at least 3 min on the treadmill at 1.25 m/s before any data were collected, but the accommodation period may not have been long enough for the participants to adapt to each prosthetic configuration. However, we pseudo-randomized the trial order to mitigate any potential training or adaptation effects. In addition, a prosthetist aligned the recommended category prosthesis to each participant and the same alignment was used for all subsequent stiffness categories. In practice, prosthetists typically re-align the prosthesis when the stiffness category is changed. However, we kept the alignment the same for each prosthetic stiffness category to examine the effect of prosthetic stiffness independently from any potential effects of prosthetic alignment. Furthermore, we set the BiOM power setting up to 20% higher than recommended, which may have been too high for the motor of the BiOM to generate the required power. For example, there was little difference in the average positive affected leg work between use of the BiOM at the +10% and +20% settings when walking at 1.50 and 1.75 m/s (Figure 3), which may be related to device limitations, and may have affected the trailing affected leg positive work. Future studies should determine the effects of different prosthetic stiffness and power configurations on joint mechanics of the prosthetic ankle compared to the biological ankle.

The effects of prosthetic stiffness and power on individual leg work and effective foot length ratio can be used to inform prosthetic selection. For example, our results suggest that for walking at 0.75–1.00 m/s, prosthetists should select the BiOM attached to passive-elastic prosthesis that is one category stiffer than manufacturer-recommended and with a power setting 10% or 20% greater than recommended based on biological ankle values. In addition, our results can be used to inform prosthetic design. For example, we found that setting the BiOM to power settings 10% and 20% greater than recommended based on biological ankle values resulted in affected trailing leg work that was most similar to the biological leg during walking at 0.75–1.00 m/s. This suggests that there may be some mechanical energy losses at joints proximal to the ankle and potential for improvement in prosthetic designs that are able to minimize energy losses at joints proximal to the ankle. Future studies should examine the effects of different prosthetic stiffness and power configurations on ankle, knee, and hip joint work to determine the work done by the prosthesis and how that can affect the mechanical work at more proximal joints.

## 5 Conclusion

In summary, when people with a transtibial amputation used an Össur Vari-flex low profile passive-elastic prosthesis with different stiffness categories to walk at a range of speeds, we found no effect on the positive work done by the affected trailing leg during the step-to-step transition. However, we found that use of a passive-elastic prosthesis that was one category stiffer than recommended reduced the magnitude of negative work done by the unaffected leading leg compared to a passive-elastic prosthesis two categories less stiff than recommended, which may be due in part to the greater effective foot length ratio of the +1 compared to −2 stiffness category prosthesis during walking at 0.75–1.75 m/s. When people with a transtibial amputation used the BiOM prosthesis with a power setting 10% or 20% greater than recommended based on values from non-amputees, they increased positive affected trailing leg work during the step-to-step transition compared a passive-elastic prosthesis, and this effect was greater at 1.50 and 1.75 m/s. At walking speeds of 0.75–1.00 m/s, use of the BiOM with a power setting 10% or 20% greater than recommended resulted in a reduced magnitude of negative unaffected leading leg work during the step-to-step transition compared to a passive-elastic prosthesis. However, when people with a transtibial amputation walked at 1.50 m/s and 1.75 m/s, use of the BiOM resulted in an increased magnitude of negative unaffected leading leg work compared to use of a passive-elastic prosthesis. Ultimately, our results suggest that for walking at 0.75–1.00 m/s, prosthetists should select the BiOM attached to passive-elastic prosthesis that is one category stiffer than manufacturer-recommended and with a power setting 10% or 20% greater than recommended based on biological ankle values. This prosthetic configuration can allow people with unilateral transtibial amputation to maximize positive affected trailing leg work and minimize the magnitude of negative unaffected leading leg work compared to use of the other prosthetic configurations tested. The effects of prosthetic stiffness category and power setting on individual leg work and effective foot length ratio can be used to inform prosthetic design and may allow people with a transtibial amputation to reduce the risk of joint pain and osteoarthritis.

## Data Availability

The original contributions presented in the study are included in the article/Supplementary Material, further inquiries can be directed to the corresponding author.

## Supplementary material

Supplementary Data Sheet 1 Information about Data Sheet 2.

Supplementary Data Sheet 2 Average individual leg work and effective foot length ratio data.

Supplementary Data Sheet 3 Code for statistics and figures.

Supplementary Data Sheet 4 Supplementary figures and tables.
