# Supplementary material for: Greater than recommended stiffness and power setting of a stance-phase powered leg prosthesis can improve step-to-step transition work and effective foot length ratio during walking in people with transtibial amputation
Source: Front Bioeng Biotechnol. 2024 Jul 1;12:1336520. doi: 10.3389/fbioe.2024.1336520 (PMC11246994; doi:10.3389/fbioe.2024.1336520)
Supplement: Supplementary file 1 [file DataSheet4.docx]

**Supplementary Figures and Tables**


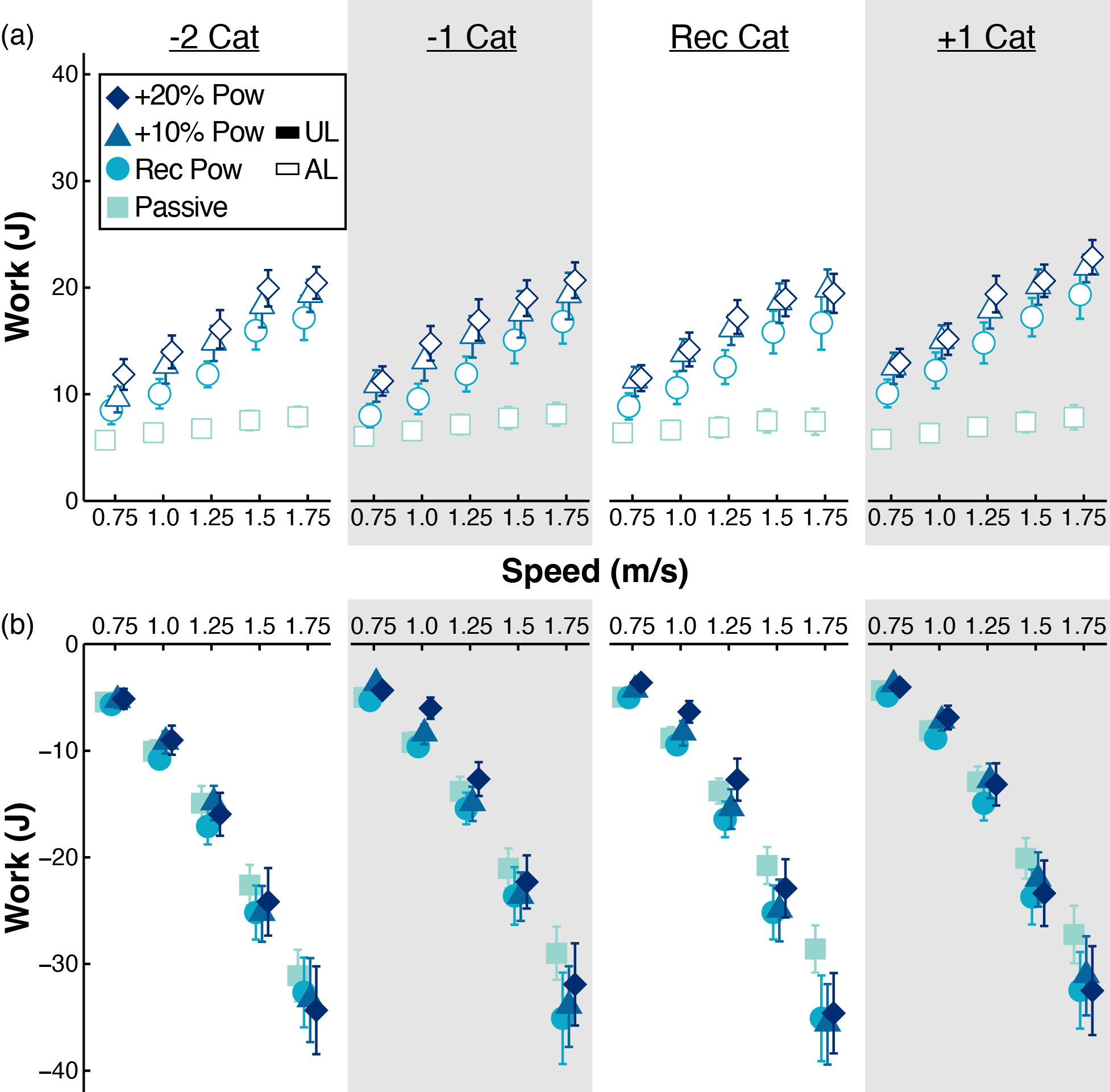


**Supplementary Figure 1.** (a) Average work (J) done by the trailing affected leg (AL) during the AL to unaffected leg (UL) step-to-step transition from all 13 participants walking at a range of speeds (m/s) using every combination of prosthetic foot stiffness category (Cat) compared to recommended (Rec; columns: -2 Cat, -1 Cat, Rec Cat, +1 Cat) and power setting (colors and symbols: Passive, Rec, +10%, +20%). (b) Average work (J) done by the leading UL during the AL to UL transition from all 13 participants walking at a range of speeds (m/s) using every combination of stiffness category (columns: -2 Cat, -1 Cat, Rec Cat, +1 Cat) and power setting (colors and symbols; Passive, Rec, +10%, +20%). Open symbols indicate the AL and filled symbols indicate the UL. Error bars are SEM and may be hidden behind the symbol. Symbols are offset for clarity.


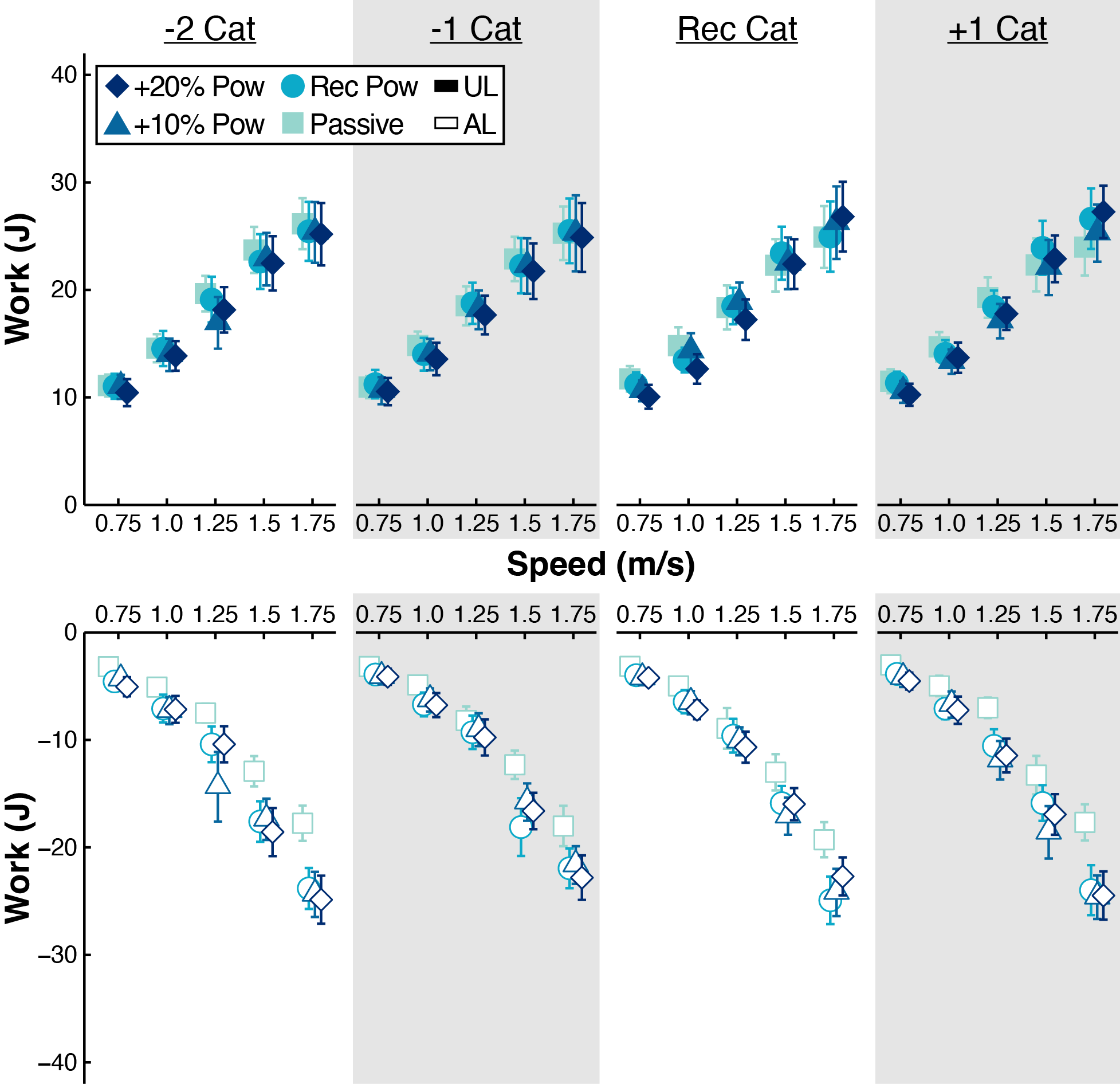


**Supplementary Figure 2.** (a) Average work (J) done by the unaffected leg (UL) during the UL to affected leg (AL) step-to-step transition from all participants walking at a range of speeds (m/s) using every combination of prosthetic foot stiffness category compared to recommended (Rec; columns: -2, -1, Rec, +1) and power setting (colors and symbols; Passive, Rec, +10%, +20%). (b) Average work (J) done by the AL during the UL to AL transition from all participants walking across a range of speeds (m/s) using every combination of stiffness categories (columns; -2, -1, Rec, +1) and power settings (colors and symbols; Passive, Rec, +10%, +20%). Open symbols indicate the AL and filled symbols indicate the UL. Error bars are SEM and may be hidden behind the symbol. Symbols are offset for clarity.


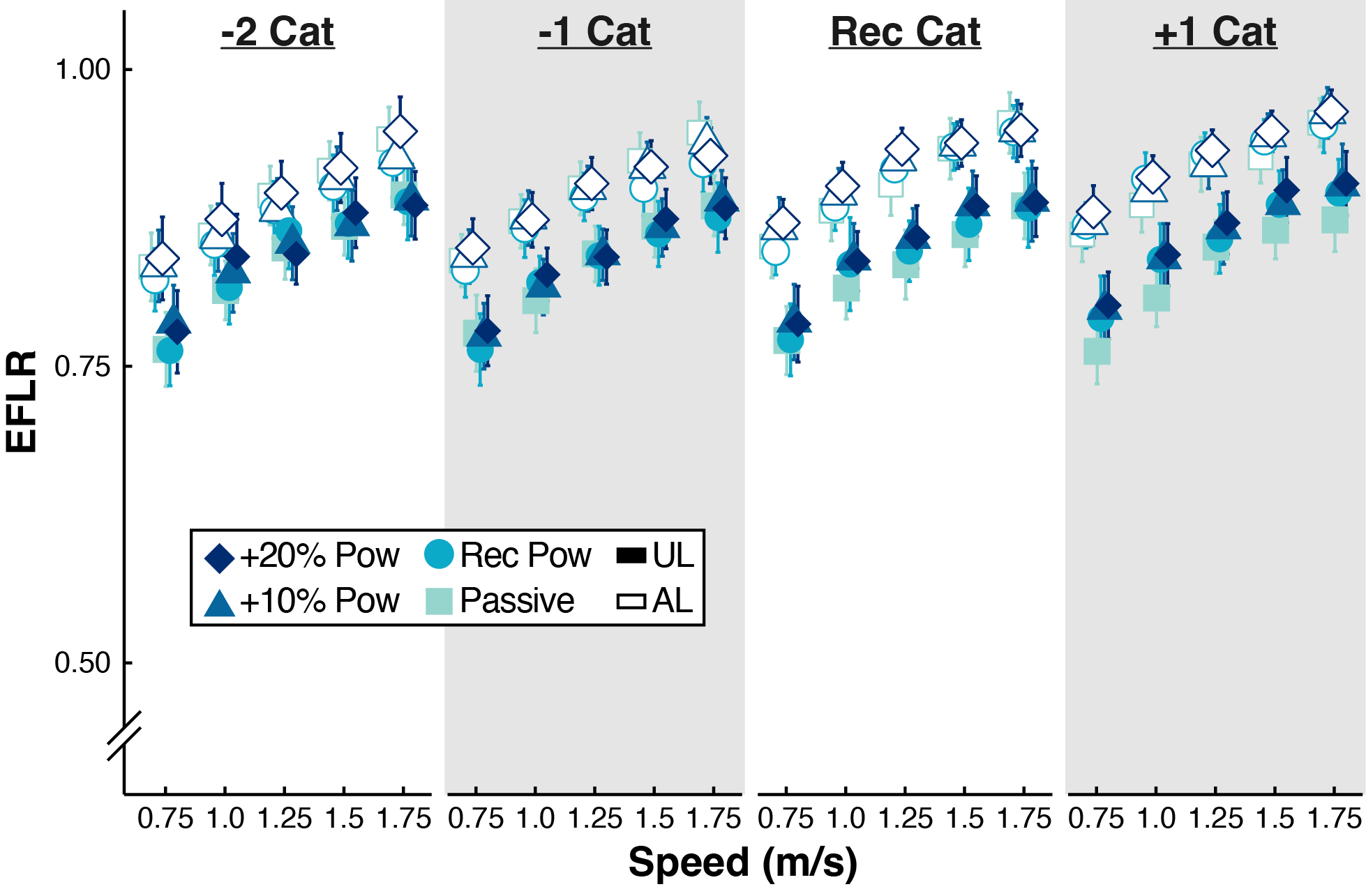


**Supplementary Figure 3.** Effective foot length ratio (EFLR) for all 13 participants walking at a range of speeds (m/s) using every combination of prosthetic foot stiffness category compared to recommended (Rec; columns: -2, -1, Rec, +1) and power setting (colors and symbols; Passive, Rec, +10%, +20%). Open symbols indicate the affected leg (AL) and filled symbols indicate the unaffected leg (UL). Error bars are SEM and may be hidden behind the symbol. Symbols are offset for clarity.

**Supplementary Table 1**. Linear mixed model parameters for the fixed effects of prosthetic foot stiffness category, power setting, speed, and the interaction of stiffness category with power setting on the positive work done by the trailing affected leg (AL_trail_ W_pos_) and the negative work done by the leading unaffected leg (UL_lead_ W_neg_) during the AL to UL step-to-step transition for every combination of stiffness category and power setting. Linear mixed models were simplified using backward elimination where non-significant (p > 0.05) interaction effects were removed. Coefficient estimates, 95% confidence intervals for coefficient estimates (CI), coefficient standard errors (SE), t values (t), and p values (p) are listed. For the stiffness categories compared to recommended (Rec; categorical; -2, -1, Rec, +1), the model coefficients are in reference to the -2 category. For the power settings (categorical; Passive, Rec, +10%, +20%), the model coefficients are in reference to the Rec stiffness category passive-elastic prosthesis. The model coefficients for speed represent the change in dependent variable for a 1 m/s increase in speed. Bold indicates a significant difference.

| **AL_trail_ W_pos_ (J)** | ***Estimate (B)*** | ***CI*** | ***SE*** | ***t*** | ***p*** |
| --- | --- | --- | --- | --- | --- |
| Intercept | -1.95 | [-4.77, 0.88] | 1.41 | -1.38 | 0.187 |
| Stiffness Category [-1] | 0.27 | [-0.66, 1.21] | 0.48 | 0.57 | 0.569 |
| Stiffness Category [Rec] | 0.11 | [-0.82, 1.05] | 0.48 | 0.23 | 0.816 |
| Stiffness Category [+1] | -0.00 | [-0.94, 0.93] | 0.48 | -0.00 | 0.996 |
| Power Setting [Rec] | 5.13 | [4.17, 6.09] | 0.49 | 10.41 | **< 0.0001** |
| Power Setting [+10%] | 7.27 | [6.31, 8.23] | 0.49 | 14.77 | **< 0.0001** |
| Power Setting [+20%] | 8.88 | [7.93, 9.84] | 0.49 | 18.04 | **< 0.0001** |
| Speed [m/s] | 7.03 | [6.55, 7.51] | 0.25 | 28.34 | **< 0.0001** |
| Stiffness Category [-1] * Power Setting [Rec] | -0.04 | [-1.39, 1.29] | 0.69 | -0.07 | 0.946 |
| Stiffness Category [Rec] * Power Setting [Rec] | 0.75 | [-0.60, 2.09] | 0.69 | 1.08 | 0.280 |
| Stiffness Category [+1] * Power Setting [Rec] | 2.03 | [0.68, 3.38] | 0.70 | 2.92 | **0.004** |
| Stiffness Category [-1] * Power Setting [+10%] | 0.72 | [-0.62, 2.06] | 0.69 | 1.05 | 0.296 |
| Stiffness Category [Rec] * Power Setting [+10%] | 1.52 | [0.18, 2.87] | 0.69 | 2.21 | **0.027** |
| Stiffness Category [+1] * Power Setting [+10%] | 2.54 | [1.19, 3.89] | 0.70 | 3.65 | **0.0003** |
| Stiffness Category [-1] * Power Setting [+20%] | 0.47 | [-0.87, 1.81] | 0.69 | 0.68 | 0.497 |
| Stiffness Category [Rec] * Power Setting [+20%] | 0.38 | [-0.96, 1.72] | 0.69 | 0.55 | 0.581 |
| Stiffness Category [+1] * Power Setting [+20%] | 1.74 | [0.38, 3.09] | 0.70 | 2.50 | **0.013** |
|  |  | | | | |
| **UL_lead_ W_neg_** **(J)** | ***Estimate (B)*** | ***CI*** | ***SE*** | ***t*** | ***p*** |
| Intercept | 18.41 | [14.14, 20.30] | 1.66 | 11.12 | **< 0.0001** |
| Stiffness Category [-1] | 1.26 | [0.32, 1.92] | 0.45 | 2.81 | **0.005** |
| Stiffness Category [Rec] | 0.98 | [0.09, 1.70] | 0.45 | 2.16 | **0.031** |
| Stiffness Category [+1] | 1.89 | [0.87, 2.49] | 0.45 | 4.15 | **< 0.0001** |
| Power Setting [Rec] | -2.22 | [-3.01, -1.42] | 0.45 | -4.97 | **< 0.0001** |
| Power Setting [+10%] | -1.35 | [-2.21, -0.62] | 0.45 | -3.02 | **0.003** |
| Power Setting [+20%] | -0.65 | [-1.46, 0.13] | 0.45 | -1.47 | 0.143 |
| Speed [m/s] | -28.09 | [-27.26, -25.64] | 0.45 | -61.80 | **< 0.0001** |

**Supplementary Table 2**. Linear mixed model parameters for the fixed effects of prosthetic foot stiffness category, power setting, speed, and the interaction of stiffness category with power setting on the positive work done by the unaffected leg (UL W_pos_) and the negative work done by the affected leg (AL W_neg_) during the UL to AL step-to-step transition for every combination of stiffness category and power setting. Linear mixed models were simplified using backward elimination where non-significant (p > 0.05) interaction effects were removed. Coefficient estimates, 95% confidence intervals for coefficient estimates (CI), coefficient standard errors (SE), t values (t), and p values (p) are listed. For the stiffness categories compared to recommended (Rec; categorical; -2, -1, Rec, +1), the model coefficients are in references to the -2 category. For the power settings (categorical; Passive, Rec, +10%, +20%), the model coefficients are in reference to the Rec stiffness category passive-elastic prosthesis. The model coefficients for speed represent the change in dependent variable for a 1 m/s increase in speed.

| **UL W_pos_ (J)** | ***Estimate (B)*** | ***CI*** | ***SE*** | ***t*** | ***p*** |
| --- | --- | --- | --- | --- | --- |
| Intercept | -0.06 | [-3.72, 3.60] | 1.81 | -0.03 | 0.973 |
| Stiffness Category [-1] | -0.01 | [-0.54, 0.52] | 0.27 | -0.02 | 0.983 |
| Stiffness Category [Rec] | 0.06 | [-0.47, 0.59] | 0.27 | 0.23 | 0.817 |
| Stiffness Category [+1] | -0.11 | [-0.64, 0.43] | 0.27 | -0.40 | 0.690 |
| Power Setting [Rec] | -0.26 | [-0.78, 0.27] | 0.27 | -0.96 | 0.340 |
| Power Setting [+10%] | -0.72 | [-1.25, -0.20] | 0.27 | -2.69 | **0.007** |
| Power Setting [+20%] | -0.83 | [-1.36, -0.30] | 0.27 | -3.08 | **0.002** |
| Speed [m/s] | 14.88 | [14.35, 15.42] | 0.27 | 54.37 | **< 0.0001** |
|  |  | | | | |
| **AL W_neg_** **(J)** | ***Estimate (B)*** | ***CI*** | ***SE*** | ***t*** | ***p*** |
| Intercept | 13.71 | [11.09, 16.33] | 1.31 | 10.44 | **< 0.0001** |
| Stiffness Category [-1] | -0.02 | [-1.21, 1.16] | 0.61 | -0.04 | 0.968 |
| Stiffness Category [Rec] | -0.57 | [-1.75, 0.62] | 0.61 | -0.93 | 0.350 |
| Stiffness Category [+1] | 0.10 | [-1.09, 1.28] | 0.61 | 0.16 | 0.876 |
| Power Setting [Rec] | -2.96 | [-4.17, -1.75] | 0.62 | -4.75 | **< 0.0001** |
| Power Setting [+10%] | -3.82 | [-5.03, -2.61] | 0.62 | -6.14 | **< 0.0001** |
| Power Setting [+20%] | -3.47 | [-4.68, -2.26] | 0.62 | -5.57 | **< 0.0001** |
| Speed [m/s] | -18.43 | [-19.04, -17.82] | 0.31 | -58.81 | **< 0.0001** |
| Stiffness Category [-1] * Power Setting [Rec] | 0.30 | [-1.39, 1.99] | 0.87 | 0.34 | 0.731 |
| Stiffness Category [Rec] * Power Setting [Rec] | 0.82 | [-0.88, 2.51] | 0.87 | 0.94 | 0.350 |
| Stiffness Category [+1] * Power Setting [Rec] | 0.34 | [-1.37, 2.05] | 0.88 | 0.39 | 0.701 |
| Stiffness Category [-1] * Power Setting [+10%] | 1.78 | [0.09, 3.48] | 0.87 | 2.05 | **0.041** |
| Stiffness Category [Rec] * Power Setting [+10%] | 1.37 | [-0.32, 3.07] | 0.87 | 1.58 | 0.116 |
| Stiffness Category [+1] * Power Setting [+10%] | 0.25 | [-1.45, 1.96] | 0.88 | 0.29 | 0.772 |
| Stiffness Category [-1] * Power Setting [+20%] | 0.80 | [-0.89, 2.49] | 0.87 | 0.92 | 0.359 |
| Stiffness Category [Rec] * Power Setting [+20%] | 1.27 | [-0.43, 2.96] | 0.87 | 1.45 | 0.146 |
| Stiffness Category [+1] * Power Setting [+20%] | 0.19 | [-1.52, 1.90] | 0.88 | 0.21 | 0.831 |

**Supplementary Table 3**. Linear mixed model parameters for the fixed effects of prosthetic foot stiffness category, power setting, leg type, speed, and the interaction of stiffness category with power setting on the effective foot length ratio (EFLR) for every combination of stiffness category and power setting. Linear mixed models were simplified using backward elimination where non-significant (p > 0.05) interaction effects were removed. Coefficient estimates, 95% confidence intervals for coefficient estimates (CI), coefficient standard errors (SE), t values (t), and p values (p) are listed. For the stiffness categories compared to recommended (Rec; categorical; -2, -1, Rec, +1), the model coefficients are in reference to the -2 category. For the power settings (categorical; Passive, Rec, +10%, +20%), the model coefficients are in reference to the Rec stiffness category passive-elastic prosthesis. The model coefficients for leg type (categorical; affected leg [AL], unaffected leg [UL]) are in reference to the AL. The model coefficients for speed represent the change in dependent variable for a 1 m/s increase in speed.

| **EFLR** | ***Estimate (B)*** | ***CI*** | ***SE*** | ***t*** | ***p*** |
| --- | --- | --- | --- | --- | --- |
| Intercept | 0.76 | [0.72, 0.81] | 0.02 | 35.69 | **< 0.0001** |
| Stiffness Category [-1] | 0.00 | [-0.01, 0.02] | 0.01 | 0.44 | 0.657 |
| Stiffness Category [Rec] | 0.01 | [-0.01, 0.02] | 0.01 | 0.93 | 0.355 |
| Stiffness Category [+1] | 0.01 | [-0.01, 0.02] | 0.01 | 1.16 | 0.246 |
| Power Setting [Rec] | -0.00 | [-0.02, 0.01] | 0.01 | -0.54 | 0.588 |
| Power Setting [+10%] | 0.00 | [-0.01, 0.01] | 0.01 | 0.26 | 0.798 |
| Power Setting [+20%] | 0.01 | [-0.00, 0.02] | 0.01 | 1.34 | 0.180 |
| Leg [UL] | -0.06 | [-0.06, -0.05] | 0.00 | -25.13 | **< 0.0001** |
| Speed [m/s] | 0.10 | [0.10, 0.11] | 0.00 | 31.61 | **< 0.0001** |
| Stiffness Category [-1] * Power Setting [Rec] | -0.01 | [-0.02, 0.01] | 0.01 | -0.55 | 0.583 |
| Stiffness Category [Rec] * Power Setting [Rec] | 0.01 | [-0.01, 0.03] | 0.01 | 1.08 | 0.283 |
| Stiffness Category [+1] * Power Setting [Rec] | 0.02 | [0.00, 0.04] | 0.01 | 2.30 | **0.022** |
| Stiffness Category [-1] * Power Setting [+10%] | -0.00 | [-0.02, 0.02] | 0.01 | -0.15 | 0.882 |
| Stiffness Category [Rec] * Power Setting [+10%] | 0.01 | [-0.00, 0.03] | 0.01 | 1.51 | 0.131 |
| Stiffness Category [+1] * Power Setting [+10%] | 0.02 | [-0.00, 0.04] | 0.01 | 1.93 | 0.054 |
| Stiffness Category [-1] * Power Setting [+20%] | -0.01 | [-0.02, 0.01] | 0.01 | -0.75 | 0.453 |
| Stiffness Category [Rec] * Power Setting [+20%] | 0.01 | [-0.01, 0.03] | 0.01 | 0.97 | 0.335 |
| Stiffness Category [+1] * Power Setting [+20%] | 0.02 | [-0.00, 0.04] | 0.01 | 1.88 | 0.061 |
