# Supplementary material for: Greater than recommended stiffness and power setting of a stance-phase powered leg prosthesis can improve step-to-step transition work and effective foot length ratio during walking in people with transtibial amputation
Source: Front Bioeng Biotechnol. 2024 Jul 1;12:1336520. doi: 10.3389/fbioe.2024.1336520 (PMC11246994; doi:10.3389/fbioe.2024.1336520)
Supplement: Supplementary file 2 [file DataSheet3.pdf]

Code for Statistics and Figures

Joshua Tacca

11/10/2023

Load Data Table

```
ps_data <- read.csv("Tacca_data.csv")
```

Prepare Data Frame

```
#reorder the stiffness categories from -2 to +1
ps_data$Stiff_cat <- factor(ps_data$Stiff_cat, ordered = FALSE)
ps_data$Stiff_cat <- relevel(ps_data$Stiff_cat, "down2")

#Make a column with step-to-step transition and stiffness category combination and make sure it is in t
ps_data$Stiff_cat_num <- ifelse(ps_data$Stiff_cat == "down2", "adown2",
                                ifelse(ps_data$Stiff_cat == "down1", "down1",
                                       ifelse(ps_data$Stiff_cat == "rec", "rec", "up1")))

ps_data$Trans.Stiff <- paste(ps_data$Transition, ps_data$Stiff_cat_num)

#Make a column with step-to-step transition and power setting combination
ps_data$Trans.Pow <- paste(ps_data$Transition, ps_data$Power)

#Make a column with leg and stiffness category combination
ps_data$Leg.Stiff <- paste(ps_data$Leg, ps_data$Stiff_cat_num)

#Make a column with leg and power setting combination
ps_data$Leg.Pow <- paste(ps_data$Leg, ps_data$Power)

ps_data_passive <- ps_data[ps_data$Power == "no_power",] #trials without the BiOM
ps_data_krec <- ps_data[ps_data$Stiff_cat == "rec",] #trials with recommended stiffness category with a
```

Statistics

Table 2: LME effect of stiffness category on W during AL to UL transition

```
#AL2UL Transition (Wpos_trail: AL is trail leg; passive)
lme.wpos_al2ul_passive <- lmer(Wpos_trail ~ Stiff_cat*Speed + (1|Sub), data = ps_data_passive[ps_data_pa
summary(lme.wpos_al2ul_passive)
```

```
## Linear mixed model fit by REML. t-tests use Satterthwaite's method [
## lmerModLmerTest]
## Formula: Wpos_trail ~ Stiff_cat * Speed + (1 | Sub)
##    Data: ps_data_passive[ps_data_passive$Transition == "AL2UL", ]
```

```
##
## REML criterion at convergence: 819.4
##
## Scaled residuals:
##     Min      1Q  Median      3Q     Max
## -3.3262 -0.4819 -0.0449  0.4984  3.6640
##
## Random effects:
##  Groups   Name        Variance Std.Dev.
##  Sub      (Intercept) 9.349    3.058
##  Residual             1.126    1.061
## Number of obs: 256, groups:  Sub, 13
##
## Fixed effects:
##                        Estimate Std. Error       df t value Pr(>|t|)
## (Intercept)              4.2038     0.9787  20.9182   4.295 0.000323 ***
## Stiff_catdown1           0.3504     0.6905 236.0018   0.507 0.612314
## Stiff_catrec             1.3925     0.6905 236.0018   2.017 0.044860 *
## Stiff_catup1             0.1893     0.6905 236.0018   0.274 0.784166
## Speed                    2.1065     0.3786 236.0084   5.564 7.15e-08 ***
## Stiff_catdown1:Speed    -0.0617     0.5350 236.0018  -0.115 0.908281
## Stiff_catrec:Speed      -1.0312     0.5350 236.0018  -1.928 0.055100 .
## Stiff_catup1:Speed      -0.1542     0.5350 236.0018  -0.288 0.773434
## ---
## Signif. codes:  0 '***' 0.001 '**' 0.01 '*' 0.05 '.' 0.1 ' ' 1
##
## Correlation of Fixed Effects:
##             (Intr) Stff_ctd1 Stff_c Stff_ctp1 Speed  Stff_ctd1:S Stf_:S
## Stff_ctdwn1 -0.353
## Stiff_catrc -0.353  0.500
## Stiff_catp1 -0.353  0.500     0.500
## Speed       -0.480  0.680     0.680  0.680
## Stff_ctd1:S  0.339 -0.962    -0.481 -0.481    -0.706
## Stff_ctrc:S  0.339 -0.481    -0.962 -0.481    -0.706  0.500
## Stff_ctp1:S  0.339 -0.481    -0.481 -0.962    -0.706  0.500       0.500
```

```r
lme.wpos_al2ul_passive2 <- lmer(Wpos_trail ~ Stiff_cat + Speed + (1|Sub), data = ps_data_passive[ps_data
summary(lme.wpos_al2ul_passive2)
```

```
## Linear mixed model fit by REML. t-tests use Satterthwaite's method [
## lmerModLmerTest]
## Formula: Wpos_trail ~ Stiff_cat + Speed + (1 | Sub)
##    Data: ps_data_passive[ps_data_passive$Transition == "AL2UL", ]
##
## REML criterion at convergence: 825.3
##
## Scaled residuals:
##     Min      1Q  Median      3Q     Max
## -3.6561 -0.4599 -0.0393  0.4914  3.9816
##
## Random effects:
##  Groups   Name        Variance Std.Dev.
##  Sub      (Intercept) 9.349    3.058
##  Residual             1.135    1.065
```

```
## Number of obs: 256, groups:  Sub, 13
##
## Fixed effects:
##                    Estimate Std. Error         df t value Pr(>|t|)
## (Intercept)        4.591085   0.890386  14.400177   5.156 0.000133 ***
## Stiff_catdown1     0.273750   0.188353 239.001858   1.453 0.147428
## Stiff_catrec       0.111562   0.188353 239.001858   0.592 0.554207
## Stiff_catup1      -0.002188   0.188353 239.001858  -0.012 0.990743
## Speed              1.794730   0.190595 239.028398   9.416  < 2e-16 ***
## ---
## Signif. codes:  0 '***' 0.001 '**' 0.01 '*' 0.05 '.' 0.1 ' ' 1
##
## Correlation of Fixed Effects:
##             (Intr) Stff_ctd1 Stff_c Stff_ctp1
## Stff_ctdwn1 -0.106
## Stiff_catrc -0.106  0.500
## Stiff_catp1 -0.106  0.500     0.500
## Speed       -0.266  0.000     0.000  0.000
```

```r
confint(lme.wpos_al2ul_passive2)
```

```
## Computing profile confidence intervals ...
```

```
##                      2.5 %    97.5 %
## .sig01           2.08207810 4.5718512
## .sigma           0.96926855 1.1580682
## (Intercept)      2.79856879 6.3839264
## Stiff_catdown1 -0.09381624 0.6413162
## Stiff_catrec   -0.25600374 0.4791287
## Stiff_catup1   -0.36975374 0.3653787
## Speed           1.42285939 2.1667402
```

```r
#AL2UL Transition (Wneg_lead: UL is lead leg; passive)
lme.wneg_al2ul_passive <- lmer(Wneg_lead ~ Stiff_cat*Speed + (1|Sub), data = ps_data_passive[ps_data_pa
summary(lme.wneg_al2ul_passive)
```

```
## Linear mixed model fit by REML. t-tests use Satterthwaite's method [
## lmerModLmerTest]
## Formula: Wneg_lead ~ Stiff_cat * Speed + (1 | Sub)
##    Data: ps_data_passive[ps_data_passive$Transition == "AL2UL", ]
##
## REML criterion at convergence: 1412.6
##
## Scaled residuals:
##     Min      1Q  Median      3Q     Max
## -3.4217 -0.5091  0.0943  0.6974  2.1644
##
## Random effects:
##  Groups   Name        Variance Std.Dev.
##  Sub      (Intercept) 18.67    4.321
##  Residual             13.41    3.662
## Number of obs: 256, groups:  Sub, 13
```

```
##
## Fixed effects:
##                       Estimate Std. Error       df t value Pr(>|t|)
## (Intercept)            15.1925     2.0682  84.0727   7.346 1.21e-10 ***
## Stiff_catdown1         -0.8286     2.3824 236.0091  -0.348    0.728
## Stiff_catrec           -0.9064     2.3824 236.0091  -0.380    0.704
## Stiff_catup1           -0.8038     2.3824 236.0091  -0.337    0.736
## Speed                 -25.6258     1.3063 236.0465 -19.617  < 2e-16 ***
## Stiff_catdown1:Speed    1.6291     1.8457 236.0091   0.883    0.378
## Stiff_catrec:Speed      1.8720     1.8457 236.0091   1.014    0.312
## Stiff_catup1:Speed      2.4556     1.8457 236.0091   1.330    0.185
## ---
## Signif. codes:  0 '***' 0.001 '**' 0.01 '*' 0.05 '.' 0.1 ' ' 1
##
## Correlation of Fixed Effects:
##             (Intr) Stff_ctd1 Stff_c Stff_ctp1 Speed  Stff_ctd1:S Stf_:S
## Stff_ctdwn1 -0.576
## Stiff_catrc -0.576  0.500
## Stiff_catp1 -0.576  0.500     0.500
## Speed       -0.784  0.680     0.680  0.680
## Stff_ctd1:S  0.554 -0.962    -0.481 -0.481    -0.706
## Stff_ctrc:S  0.554 -0.481    -0.962 -0.481    -0.706  0.500
## Stff_ctp1:S  0.554 -0.481    -0.481 -0.962    -0.706  0.500       0.500
```

```
lme.wneg_al2ul_passive2 <- lmer(Wneg_lead ~ Stiff_cat+Speed + (1|Sub), data = ps_data_passive[ps_data_pa
summary(lme.wneg_al2ul_passive2)
```

```
## Linear mixed model fit by REML. t-tests use Satterthwaite's method [
## lmerModLmerTest]
## Formula: Wneg_lead ~ Stiff_cat + Speed + (1 | Sub)
##    Data: ps_data_passive[ps_data_passive$Transition == "AL2UL", ]
##
## REML criterion at convergence: 1423
##
## Scaled residuals:
##     Min      1Q  Median      3Q     Max
## -3.6364 -0.4932  0.0955  0.6938  2.2821
##
## Random effects:
##  Groups   Name        Variance Std.Dev.
##  Sub      (Intercept) 18.68    4.322
##  Residual             13.35    3.654
## Number of obs: 256, groups:  Sub, 13
##
## Fixed effects:
##                 Estimate Std. Error       df t value Pr(>|t|)
## (Intercept)      13.3427     1.5174  28.2847   8.793 1.39e-09 ***
## Stiff_catdown1    1.1950     0.6459 239.0091   1.850 0.065513 .
## Stiff_catrec      1.4189     0.6459 239.0091   2.197 0.028987 *
## Stiff_catup1      2.2466     0.6459 239.0091   3.478 0.000599 ***
## Speed           -24.1367     0.6535 239.1585 -36.937  < 2e-16 ***
## ---
## Signif. codes:  0 '***' 0.001 '**' 0.01 '*' 0.05 '.' 0.1 ' ' 1
##
```

```
## Correlation of Fixed Effects:
##             (Intr) Stff_ctd1 Stff_c Stff_ctp1
## Stff_ctdwn1 -0.213
## Stiff_catrc -0.213  0.500
## Stiff_catp1 -0.213  0.500     0.500
## Speed       -0.534  0.000     0.000  0.000
```

```
confint(lme.wneg_al2ul_passive2)
```

```
## Computing profile confidence intervals ...
```

```
##                        2.5 %     97.5 %
## .sig01           2.89306873   6.515425
## .sigma           3.32361445   3.970996
## (Intercept)     10.35611696  16.331317
## Stiff_catdown1  -0.06538007   2.455380
## Stiff_catrec     0.15852618   2.679286
## Stiff_catup1     0.98618243   3.506943
## Speed          -25.41126625 -22.860839
```

Table 3: LME effect of stiffness category on W during UL to AL transition

```
#UL2AL Transition (Wpos_trail: UL is trail leg; passive)
lme.wpos_ul2al_passive <- lmer(Wpos_trail ~ Stiff_cat*Speed + (1|Sub), data = ps_data_passive[ps_data_pa
summary(lme.wpos_ul2al_passive) #no interaction
```

```
## Linear mixed model fit by REML. t-tests use Satterthwaite's method [
## lmerModLmerTest]
## Formula: Wpos_trail ~ Stiff_cat * Speed + (1 | Sub)
##    Data: ps_data_passive[ps_data_passive$Transition == "UL2AL", ]
##
## REML criterion at convergence: 1282.1
##
## Scaled residuals:
##      Min       1Q   Median       3Q      Max
## -3.05519 -0.55584  0.08527  0.70037  2.52488
##
## Random effects:
##  Groups   Name        Variance Std.Dev.
##  Sub      (Intercept) 38.85    6.233
##  Residual              7.44    2.728
## Number of obs: 256, groups:  Sub, 13
##
## Fixed effects:
##                       Estimate Std. Error       df t value Pr(>|t|)
## (Intercept)            -0.2877     2.1366  27.1010  -0.135   0.8939
## Stiff_catdown1          0.7409     1.7747 236.0011   0.417   0.6767
## Stiff_catrec            2.0947     1.7747 236.0011   1.180   0.2391
## Stiff_catup1            2.6371     1.7747 236.0011   1.486   0.1386
## Speed                  15.4068     0.9731 236.0114  15.832   <2e-16 ***
## Stiff_catdown1:Speed   -1.0372     1.3749 236.0011  -0.754   0.4514
## Stiff_catrec:Speed     -2.1786     1.3749 236.0011  -1.585   0.1144
```

```
## Stiff_catup1:Speed    -2.6536     1.3749 236.0011  -1.930   0.0548 .
## ---
## Signif. codes:  0 '***' 0.001 '**' 0.01 '*' 0.05 '.' 0.1 ' ' 1
##
## Correlation of Fixed Effects:
##             (Intr) Stff_ctd1 Stff_c Stff_ctp1 Speed  Stff_ctd1:S Stf_:S
## Stff_ctdwn1 -0.415
## Stiff_catrc -0.415  0.500
## Stiff_catp1 -0.415  0.500     0.500
## Speed       -0.566  0.680     0.680  0.680
## Stff_ctd1:S  0.400 -0.962    -0.481 -0.481    -0.706
## Stff_ctrc:S  0.400 -0.481    -0.962 -0.481    -0.706  0.500
## Stff_ctp1:S  0.400 -0.481    -0.481 -0.962    -0.706  0.500       0.500
```

```
lme.wpos_ul2al_passive2 <- lmer(Wpos_trail ~ Stiff_cat + Speed + (1|Sub), data = ps_data_passive[ps_data
summary(lme.wpos_ul2al_passive2)
```

```
## Linear mixed model fit by REML. t-tests use Satterthwaite's method [
## lmerModLmerTest]
## Formula: Wpos_trail ~ Stiff_cat + Speed + (1 | Sub)
##    Data: ps_data_passive[ps_data_passive$Transition == "UL2AL", ]
##
## REML criterion at convergence: 1293.3
##
## Scaled residuals:
##     Min      1Q  Median      3Q     Max
## -3.2660 -0.5211  0.0836  0.6853  2.4503
##
## Random effects:
##  Groups   Name        Variance Std.Dev.
##  Sub      (Intercept) 38.847   6.233
##  Residual              7.487   2.736
## Number of obs: 256, groups:  Sub, 13
##
## Fixed effects:
##                 Estimate Std. Error       df t value Pr(>|t|)
## (Intercept)       1.5350     1.8638  15.8863   0.824    0.422
## Stiff_catdown1   -0.5475     0.4837 239.0011  -1.132    0.259
## Stiff_catrec     -0.6116     0.4837 239.0011  -1.264    0.207
## Stiff_catup1     -0.6592     0.4837 239.0011  -1.363    0.174
## Speed            13.9394     0.4894 239.0430  28.481   <2e-16 ***
## ---
## Signif. codes:  0 '***' 0.001 '**' 0.01 '*' 0.05 '.' 0.1 ' ' 1
##
## Correlation of Fixed Effects:
##             (Intr) Stff_ctd1 Stff_c Stff_ctp1
## Stff_ctdwn1 -0.130
## Stiff_catrc -0.130  0.500
## Stiff_catp1 -0.130  0.500     0.500
## Speed       -0.326  0.000     0.000  0.000
```

```
confint(lme.wpos_ul2al_passive2)
```

```
## Computing profile confidence intervals ...
```

```
##                     2.5 %     97.5 %
## .sig01          4.235513  9.3289935
## .sigma          2.489068  2.9739036
## (Intercept)    -2.201081  5.2726302
## Stiff_catdown1 -1.491405  0.3964050
## Stiff_catrec   -1.555467  0.3323425
## Stiff_catup1   -1.603124  0.2846862
## Speed          12.984616 14.8948669
```

```
#UL2AL Transition (Wneg_lead: AL is lead leg; passive)
lme.wneg_ul2al_passive <- lmer(Wneg_lead ~ Stiff_cat*Speed + (1|Sub), data = ps_data_passive[ps_data_pa
summary(lme.wneg_ul2al_passive) #no interaction
```

```
## Linear mixed model fit by REML. t-tests use Satterthwaite's method [
## lmerModLmerTest]
## Formula: Wneg_lead ~ Stiff_cat * Speed + (1 | Sub)
##    Data: ps_data_passive[ps_data_passive$Transition == "UL2AL", ]
##
## REML criterion at convergence: 1317.2
##
## Scaled residuals:
##     Min      1Q  Median      3Q     Max
## -4.7776 -0.5263  0.0914  0.6371  2.0458
##
## Random effects:
##  Groups   Name        Variance Std.Dev.
##  Sub      (Intercept) 11.686   3.418
##  Residual              9.166   3.028
## Number of obs: 256, groups:  Sub, 13
##
## Fixed effects:
##                       Estimate Std. Error        df t value Pr(>|t|)
## (Intercept)            9.30670    1.68555  91.50606   5.521 3.12e-07 ***
## Stiff_catdown1         0.02052    1.96983 236.00603   0.010    0.992
## Stiff_catrec           1.05268    1.96983 236.00603   0.534    0.594
## Stiff_catup1           0.40532    1.96983 236.00603   0.206    0.837
## Speed                -14.89278    1.08009 236.04662 -13.788  < 2e-16 ***
## Stiff_catdown1:Speed  -0.03652    1.52612 236.00603  -0.024    0.981
## Stiff_catrec:Speed    -1.30568    1.52612 236.00603  -0.856    0.393
## Stiff_catup1:Speed    -0.25032    1.52612 236.00603  -0.164    0.870
## ---
## Signif. codes:  0 '***' 0.001 '**' 0.01 '*' 0.05 '.' 0.1 ' ' 1
##
## Correlation of Fixed Effects:
##             (Intr) Stff_ctd1 Stff_c Stff_ctp1 Speed  Stff_ctd1:S Stf_:S
## Stff_ctdwn1 -0.584
## Stiff_catrc -0.584  0.500
## Stiff_catp1 -0.584  0.500     0.500
## Speed       -0.796  0.680     0.680  0.680
## Stff_ctd1:S  0.562 -0.962    -0.481 -0.481    -0.706
## Stff_ctrc:S  0.562 -0.481    -0.962 -0.481    -0.706  0.500
## Stff_ctp1:S  0.562 -0.481    -0.481 -0.962    -0.706  0.500       0.500
```

```r
lme.wneg_ul2al_passive2 <- lmer(Wneg_lead ~ Stiff_cat + Speed + (1|Sub), data = ps_data_passive[ps_data_
summary(lme.wneg_ul2al_passive2)
```

```
## Linear mixed model fit by REML. t-tests use Satterthwaite's method [
## lmerModLmerTest]
## Formula: Wneg_lead ~ Stiff_cat + Speed + (1 | Sub)
##    Data: ps_data_passive[ps_data_passive$Transition == "UL2AL", ]
##
## REML criterion at convergence: 1325.6
##
## Scaled residuals:
##     Min      1Q  Median      3Q     Max
## -4.7999 -0.5682  0.0907  0.6396  2.1151
##
## Random effects:
##  Groups   Name        Variance Std.Dev.
##  Sub      (Intercept) 11.690   3.419
##  Residual              9.088   3.015
## Number of obs: 256, groups:  Sub, 13
##
## Fixed effects:
##                 Estimate Std. Error        df t value Pr(>|t|)
## (Intercept)      9.80132    1.22011  29.91110   8.033 5.88e-09 ***
## Stiff_catdown1  -0.02484    0.53293 239.00598  -0.047    0.963
## Stiff_catrec    -0.56922    0.53293 239.00598  -1.068    0.287
## Stiff_catup1     0.09437    0.53293 239.00598   0.177    0.860
## Speed          -15.29098    0.53919 239.16779 -28.359  < 2e-16 ***
## ---
## Signif. codes:  0 '***' 0.001 '**' 0.01 '*' 0.05 '.' 0.1 ' ' 1
##
## Correlation of Fixed Effects:
##             (Intr) Stff_ctd1 Stff_c Stff_ctp1
## Stff_ctdwn1 -0.218
## Stiff_catrc -0.218  0.500
## Stiff_catp1 -0.218  0.500     0.500
## Speed       -0.548  0.000     0.000  0.000
```

```r
confint(lme.wneg_ul2al_passive2)
```

```
## Computing profile confidence intervals ...
```

```
##                      2.5 %      97.5 %
## .sig01           2.2843977   5.1593386
## .sigma           2.7424512   3.2766366
## (Intercept)      7.4029754  12.2016734
## Stiff_catdown1  -1.0648359   1.0151484
## Stiff_catrec    -1.6092109   0.4707734
## Stiff_catup1    -0.9456172   1.1343672
## Speed          -16.3425390 -14.2380878
```

Table 4: LME effect of power setting on W during AL to UL transition

```r
#AL2UL Transition (Wpos_trail: AL is trail leg; krec)
lme.wpos_al2ul_krec <- lmer(Wpos_trail ~ Power*Speed + (1|Sub), data = ps_data_krec[ps_data_krec$Transi
summary(lme.wpos_al2ul_krec)
```

```
## Linear mixed model fit by REML. t-tests use Satterthwaite's method [
## lmerModLmerTest]
## Formula: Wpos_trail ~ Power * Speed + (1 | Sub)
##    Data: ps_data_krec[ps_data_krec$Transition == "AL2UL", ]
##
## REML criterion at convergence: 1201.2
##
## Scaled residuals:
##     Min      1Q  Median      3Q     Max
## -3.2506 -0.5480  0.0417  0.5691  2.9737
##
## Random effects:
##  Groups   Name        Variance Std.Dev.
##  Sub      (Intercept) 23.027   4.799
##  Residual              5.756   2.399
## Number of obs: 253, groups:  Sub, 13
##
## Fixed effects:
##                    Estimate Std. Error       df t value Pr(>|t|)
## (Intercept)          5.7489     1.7295  32.6123   3.324   0.0022 **
## Powerrec            -2.9582     1.5695 233.0087  -1.885   0.0607 .
## Powerten            -0.5703     1.5695 233.0087  -0.363   0.7167
## Powertwenty          0.4397     1.5695 233.0087   0.280   0.7796
## Speed                0.9227     0.8559 233.0181   1.078   0.2822
## Powerrec:Speed       7.1082     1.2203 233.0112   5.825 1.89e-08 ***
## Powerten:Speed       7.5420     1.2203 233.0112   6.180 2.83e-09 ***
## Powertwenty:Speed    7.1032     1.2203 233.0112   5.821 1.93e-08 ***
## ---
## Signif. codes:  0 '***' 0.001 '**' 0.01 '*' 0.05 '.' 0.1 ' ' 1
##
## Correlation of Fixed Effects:
##             (Intr) Powrrc Powrtn Pwrtwn Speed  Pwrr:S Pwrtn:S
## Powerrec    -0.449
## Powerten    -0.449  0.495
## Powertwenty -0.449  0.495  0.495
## Speed       -0.615  0.676  0.676  0.676
## Powerrc:Spd  0.430 -0.962 -0.475 -0.475 -0.700
## Powertn:Spd  0.430 -0.475 -0.962 -0.475 -0.700  0.492
## Pwrtwnty:Sp  0.430 -0.475 -0.475 -0.962 -0.700  0.492  0.492
```

```r
confint(lme.wpos_al2ul_krec)
```

```
## Computing profile confidence intervals ...
```

```
##                        2.5 %     97.5 %
## .sig01             3.2562084 7.18859889
## .sigma             2.1672505 2.59227508
## (Intercept)        2.3626272 9.13626784
```

```
## Powerrec          -6.0014550 0.08474625
## Powerten          -3.6134850 2.47271627
## Powertwenty       -2.6035045 3.48269671
## Speed             -0.7366421 2.58258905
## Powerrec:Speed     4.7422631 9.47444581
## Powerten:Speed     5.1760623 9.90824503
## Powertwenty:Speed  4.7372357 9.46941843
```

```
#AL2UL Transition (Wneg_lead: UL is lead leg; krec)
lme.wneg_al2ul_krec <- lmer(Wneg_lead ~ Power*Speed + (1|Sub), data = ps_data_krec[ps_data_krec$Transit
summary(lme.wneg_al2ul_krec)
```

```
## Linear mixed model fit by REML. t-tests use Satterthwaite's method [
## lmerModLmerTest]
## Formula: Wneg_lead ~ Power * Speed + (1 | Sub)
##    Data: ps_data_krec[ps_data_krec$Transition == "AL2UL", ]
##
## REML criterion at convergence: 1539
##
## Scaled residuals:
##     Min      1Q  Median      3Q     Max
## -3.4188 -0.4429  0.1472  0.6607  2.1306
##
## Random effects:
##  Groups   Name        Variance Std.Dev.
##  Sub      (Intercept) 30.28    5.503
##  Residual             24.15    4.915
## Number of obs: 253, groups:  Sub, 13
##
## Fixed effects:
##                   Estimate Std. Error      df t value Pr(>|t|)
## (Intercept)         14.456      2.729  92.700   5.298 7.88e-07 ***
## Powerrec             5.164      3.215 233.018   1.606  0.10960
## Powerten             7.234      3.215 233.018   2.250  0.02537 *
## Powertwenty          8.517      3.215 233.018   2.649  0.00862 **
## Speed              -23.924      1.753 233.047 -13.645  < 2e-16 ***
## Powerrec:Speed      -6.332      2.500 233.026  -2.533  0.01197 *
## Powerten:Speed      -7.612      2.500 233.026  -3.045  0.00259 **
## Powertwenty:Speed   -7.235      2.500 233.026  -2.894  0.00416 **
## ---
## Signif. codes:  0 '***' 0.001 '**' 0.01 '*' 0.05 '.' 0.1 ' ' 1
##
## Correlation of Fixed Effects:
##             (Intr) Powrrc Powrtn Pwrtwn Speed  Pwrr:S Pwrtn:S
## Powerrec    -0.583
## Powerten    -0.583  0.495
## Powertwenty -0.583  0.495  0.495
## Speed       -0.798  0.676  0.676  0.676
## Powerrc:Spd  0.559 -0.962 -0.475 -0.475 -0.700
## Powertn:Spd  0.559 -0.475 -0.962 -0.475 -0.700  0.492
## Pwrtwnty:Sp  0.559 -0.475 -0.475 -0.962 -0.700  0.492  0.492
```

```r
confint(lme.wneg_al2ul_krec)
```

```
## Computing profile confidence intervals ...
```

```
##                        2.5 %     97.5 %
## .sig01              3.675308   8.307909
## .sigma              4.439477   5.310111
## (Intercept)         9.178237  19.735963
## Powerrec           -1.069262  11.397833
## Powerten            1.001403  13.468498
## Powertwenty         2.284055  14.751150
## Speed             -27.322552 -20.523488
## Powerrec:Speed    -11.179238  -1.485795
## Powerten:Speed    -12.459134  -2.765691
## Powertwenty:Speed -12.082247  -2.388804
```

Table 5: LME effect of power setting on W during UL to AL transition

```r
#UL2AL Transition (Wpos_trail: UL is trail leg; krec)
lme.wpos_ul2al_krec <- lmer(Wpos_trail ~ Power*Speed + (1|Sub), data = ps_data_krec[ps_data_krec$Transi
summary(lme.wpos_ul2al_krec)
```

```
## Linear mixed model fit by REML. t-tests use Satterthwaite's method [
## lmerModLmerTest]
## Formula: Wpos_trail ~ Power * Speed + (1 | Sub)
##    Data: ps_data_krec[ps_data_krec$Transition == "UL2AL", ]
##
## REML criterion at convergence: 1322
##
## Scaled residuals:
##     Min      1Q  Median      3Q     Max
## -3.4668 -0.6266  0.0877  0.6084  2.4407
##
## Random effects:
##  Groups   Name        Variance Std.Dev.
##  Sub      (Intercept) 43.763   6.615
##  Residual              9.355   3.059
## Number of obs: 253, groups:  Sub, 13
##
## Fixed effects:
##                   Estimate Std. Error      df t value Pr(>|t|)
## (Intercept)          1.708      2.313  29.158   0.739   0.4660
## Powerrec            -1.910      2.001 233.010  -0.954   0.3408
## Powerten            -2.624      2.001 233.010  -1.311   0.1910
## Powertwenty         -5.135      2.001 233.010  -2.566   0.0109 *
## Speed               13.327      1.091 233.018  12.213   <2e-16 ***
## Powerrec:Speed       1.438      1.556 233.012   0.924   0.3562
## Powerten:Speed       2.133      1.556 233.012   1.371   0.1716
## Powertwenty:Speed    3.602      1.556 233.012   2.316   0.0214 *
## ---
## Signif. codes:  0 '***' 0.001 '**' 0.01 '*' 0.05 '.' 0.1 ' ' 1
##
```

```
## Correlation of Fixed Effects:
##             (Intr) Powrrc Powrtn Pwrtwn Speed  Pwrr:S Pwrtn:S
## Powerrec    -0.428
## Powerten    -0.428  0.495
## Powertwenty -0.428  0.495  0.495
## Speed       -0.586  0.676  0.676  0.676
## Powerrc:Spd  0.410 -0.962 -0.475 -0.475 -0.700
## Powertn:Spd  0.410 -0.475 -0.962 -0.475 -0.700  0.492
## Pwrtwnty:Sp  0.410 -0.475 -0.475 -0.962 -0.700  0.492  0.492
```

```r
confint(lme.wpos_ul2al_krec)
```

```
## Computing profile confidence intervals ...
```

```
##                         2.5 %    97.5 %
## .sig01              4.4938062  9.904761
## .sigma              2.7628744  3.304705
## (Intercept)        -2.8317219  6.249545
## Powerrec           -5.7892765  1.969593
## Powerten           -6.5033938  1.255475
## Powertwenty        -9.0145477 -1.255679
## Speed              11.2111625 15.442617
## Powerrec:Speed     -1.5781376  4.454591
## Powerten:Speed     -0.8830972  5.149631
## Powertwenty:Speed   0.5861336  6.618862
```

```r
#UL2AL Transition (Wneg_lead: AL is lead leg; krec)
lme.wneg_ul2al_krec <- lmer(Wneg_lead ~ Power*Speed + (1|Sub), data = ps_data_krec[ps_data_krec$Transit
summary(lme.wneg_ul2al_krec)
```

```
## Linear mixed model fit by REML. t-tests use Satterthwaite's method [
## lmerModLmerTest]
## Formula: Wneg_lead ~ Power * Speed + (1 | Sub)
##    Data: ps_data_krec[ps_data_krec$Transition == "UL2AL", ]
##
## REML criterion at convergence: 1352.7
##
## Scaled residuals:
##     Min      1Q  Median      3Q     Max
## -4.5043 -0.5211  0.0999  0.6402  1.8804
##
## Random effects:
##  Groups   Name        Variance Std.Dev.
##  Sub      (Intercept) 15.11    3.887
##  Residual             11.25    3.355
## Number of obs: 253, groups:  Sub, 13
##
## Fixed effects:
##                    Estimate Std. Error       df t value Pr(>|t|)
## (Intercept)         10.3183     1.8832  86.8980   5.479 4.11e-07 ***
## Powerrec             2.7549     2.1945 233.0255   1.255   0.2106
## Powerten             2.1487     2.1945 233.0255   0.979   0.3285
```

```
## Powertwenty         0.1167     2.1945 233.0255   0.053   0.9576
## Speed             -16.1574     1.1968 233.0526 -13.500  < 2e-16 ***
## Powerrec:Speed     -3.9479     1.7063 233.0328  -2.314   0.0216 *
## Powerten:Speed     -3.7042     1.7063 233.0328  -2.171   0.0310 *
## Powertwenty:Speed  -1.8551     1.7063 233.0328  -1.087   0.2781
## ---
## Signif. codes:  0 '***' 0.001 '**' 0.01 '*' 0.05 '.' 0.1 ' ' 1
##
## Correlation of Fixed Effects:
##             (Intr) Powrrc Powrtn Pwrtwn Speed  Pwrr:S Pwrtn:S
## Powerrec    -0.576
## Powerten    -0.576  0.495
## Powertwenty -0.576  0.495  0.495
## Speed       -0.789  0.676  0.676  0.676
## Powerrc:Spd  0.553 -0.962 -0.475 -0.475 -0.700
## Powertn:Spd  0.553 -0.475 -0.962 -0.475 -0.700  0.492
## Pwrtwnty:Sp  0.553 -0.475 -0.475 -0.962 -0.700  0.492  0.492
```

```r
confint(lme.wneg_ul2al_krec)
```

```
## Computing profile confidence intervals ...
```

```
##                        2.5 %      97.5 %
## .sig01              2.600524   5.8631726
## .sigma              3.030418   3.6247067
## (Intercept)         6.674431  13.9627738
## Powerrec           -1.499484   7.0106435
## Powerten           -2.105679   6.4044479
## Powertwenty        -4.137697   4.3724297
## Speed             -18.477540 -13.8364747
## Powerrec:Speed     -7.256990  -0.6401699
## Powerten:Speed     -7.013256  -0.3964359
## Powertwenty:Speed  -5.164161   1.4526593
```

Appendix Table 1: Interaction between stiffness category and power setting on W during AL to UL transition

```r
lme.wpos_al2ul_sp <- lmer(Wpos_trail ~ Stiff_cat*Power + Speed + (1|Sub), data = ps_data[ps_data$Transi
summary(lme.wpos_al2ul_sp)
```

```
## Linear mixed model fit by REML. t-tests use Satterthwaite's method [
## lmerModLmerTest]
## Formula: Wpos_trail ~ Stiff_cat * Power + Speed + (1 | Sub)
##    Data: ps_data[ps_data$Transition == "AL2UL", ]
##
## REML criterion at convergence: 4851.8
##
## Scaled residuals:
##     Min      1Q  Median      3Q     Max
## -3.3030 -0.6193 -0.0257  0.6433  3.7042
##
## Random effects:
```

```
##  Groups   Name        Variance Std.Dev.
##  Sub      (Intercept) 22.999   4.796
##  Residual              7.415   2.723
## Number of obs: 991, groups:  Sub, 13
##
## Fixed effects:
##                             Estimate Std. Error         df t value Pr(>|t|)
## (Intercept)                -1.945809   1.407021  14.885207  -1.383 0.187082
## Stiff_catdown1              0.273750   0.481366 961.991848   0.569 0.569697
## Stiff_catrec                0.111563   0.481366 961.991848   0.232 0.816772
## Stiff_catup1               -0.002187   0.481366 961.991848  -0.005 0.996375
## Powerrec                    5.126415   0.492451 962.041739  10.410  < 2e-16
## Powerten                    7.270991   0.492451 962.041739  14.765  < 2e-16
## Powertwenty                 8.883703   0.492451 962.041739  18.040  < 2e-16
## Speed                       7.031664   0.248096 962.065659  28.342  < 2e-16
## Stiff_catdown1:Powerrec    -0.045790   0.688638 962.017374  -0.066 0.946999
## Stiff_catrec:Powerrec       0.746525   0.690014 962.018528   1.082 0.279569
## Stiff_catup1:Powerrec       2.029137   0.695028 961.991848   2.920 0.003588
## Stiff_catdown1:Powerten     0.720572   0.688638 962.017374   1.046 0.295652
## Stiff_catrec:Powerten       1.525282   0.690014 962.018528   2.211 0.027305
## Stiff_catup1:Powerten       2.539815   0.695028 961.991848   3.654 0.000272
## Stiff_catdown1:Powertwenty  0.469579   0.688638 962.017374   0.682 0.495469
## Stiff_catrec:Powertwenty    0.380983   0.690014 962.018528   0.552 0.580982
## Stiff_catup1:Powertwenty    1.735069   0.695028 961.991848   2.496 0.012712
##
## (Intercept)
## Stiff_catdown1
## Stiff_catrec
## Stiff_catup1
## Powerrec                   ***
## Powerten                   ***
## Powertwenty                ***
## Speed                      ***
## Stiff_catdown1:Powerrec
## Stiff_catrec:Powerrec
## Stiff_catup1:Powerrec      **
## Stiff_catdown1:Powerten
## Stiff_catrec:Powerten      *
## Stiff_catup1:Powerten      ***
## Stiff_catdown1:Powertwenty
## Stiff_catrec:Powertwenty
## Stiff_catup1:Powertwenty   *
## ---
## Signif. codes:  0 '***' 0.001 '**' 0.01 '*' 0.05 '.' 0.1 ' ' 1
```

```
##
## Correlation matrix not shown by default, as p = 17 > 12.
## Use print(x, correlation=TRUE)  or
##     vcov(x)        if you need it
```

```
confint(lme.wpos_al2ul_sp)
```

```
## Computing profile confidence intervals ...
```

```
##                                  2.5 %    97.5 %
## .sig01                       3.2682679 7.1676190
## .sigma                       2.5852517 2.8249067
## (Intercept)                 -4.7742257 0.8831113
## Stiff_catdown1              -0.6628817 1.2103817
## Stiff_catrec                -0.8250692 1.0481942
## Stiff_catup1                -0.9388192 0.9344442
## Powerrec                     4.1685065 6.0849172
## Powerten                     6.3130828 8.2294934
## Powertwenty                  7.9257947 9.8422053
## Speed                        6.5490133 7.5144951
## Stiff_catdown1:Powerrec     -1.3860258 1.2938521
## Stiff_catrec:Powerrec       -0.5963872 2.0888473
## Stiff_catup1:Powerrec        0.6767671 3.3815062
## Stiff_catdown1:Powerten     -0.6196646 2.0602133
## Stiff_catrec:Powerten        0.1823698 2.8676044
## Stiff_catup1:Powerten        1.1874451 3.8921842
## Stiff_catdown1:Powertwenty -0.8706577 1.8092202
## Stiff_catrec:Powertwenty   -0.9619293 1.7233052
## Stiff_catup1:Powertwenty    0.3826993 3.0874384
```

```
lme.wneg_al2ul_sp <- lmer(Wneg_lead ~ Stiff_cat*Power + Speed + (1|Sub), data = ps_data[ps_data$Transiti
summary(lme.wneg_al2ul_sp) #no interaction between stiffness and power
```

```
## Linear mixed model fit by REML. t-tests use Satterthwaite's method [
## lmerModLmerTest]
## Formula: Wneg_lead ~ Stiff_cat * Power + Speed + (1 | Sub)
##    Data: ps_data[ps_data$Transition == "AL2UL", ]
##
## REML criterion at convergence: 6022.3
##
## Scaled residuals:
##     Min      1Q  Median      3Q     Max
## -5.6763 -0.5167  0.1411  0.6844  2.4060
##
## Random effects:
##  Groups   Name        Variance Std.Dev.
##  Sub      (Intercept) 29.22    5.405
##  Residual             24.96    4.996
## Number of obs: 991, groups:  Sub, 13
##
## Fixed effects:
##                             Estimate Std. Error        df t value Pr(>|t|)
## (Intercept)                 18.23126    1.71944  20.29431  10.603    1e-09 ***
## Stiff_catdown1               1.19500    0.88314 962.01376   1.353   0.1763
## Stiff_catrec                 1.41891    0.88314 962.01376   1.607   0.1085
## Stiff_catup1                 2.24656    0.88314 962.01376   2.544   0.0111 *
## Powerrec                    -1.57464    0.90346 962.14367  -1.743   0.0817 .
## Powerten                    -0.90769    0.90346 962.14367  -1.005   0.3153
## Powertwenty                 -0.98836    0.90346 962.14367  -1.094   0.2742
## Speed                      -28.08882    0.45516 962.20671 -61.712   <2e-16 ***
## Stiff_catdown1:Powerrec     -0.55271    1.26340 962.08028  -0.437   0.6619
## Stiff_catrec:Powerrec       -1.09108    1.26592 962.08329  -0.862   0.3890
## Stiff_catup1:Powerrec       -0.91334    1.27514 962.01376  -0.716   0.4740
```

```
## Stiff_catdown1:Powerten     -0.39544    1.26340 962.08028  -0.313   0.7544
## Stiff_catrec:Powerten       -1.26692    1.26592 962.08329  -1.001   0.3172
## Stiff_catup1:Powerten       -0.04249    1.27514 962.01376  -0.033   0.9734
## Stiff_catdown1:Powertwenty   1.20180    1.26340 962.08028   0.951   0.3417
## Stiff_catrec:Powertwenty     0.56154    1.26592 962.08329   0.444   0.6574
## Stiff_catup1:Powertwenty    -0.51402    1.27514 962.01376  -0.403   0.6870
## ---
## Signif. codes:  0 '***' 0.001 '**' 0.01 '*' 0.05 '.' 0.1 ' ' 1

##
## Correlation matrix not shown by default, as p = 17 > 12.
## Use print(x, correlation=TRUE)  or
##     vcov(x)        if you need it
```

```
lme.wneg_al2ul_sp2 <- lmer(Wneg_lead ~ Stiff_cat + Power + Speed + (1|Sub), data = ps_data[ps_data$Tran
summary(lme.wneg_al2ul_sp2)
```

```
## Linear mixed model fit by REML. t-tests use Satterthwaite's method [
## lmerModLmerTest]
## Formula: Wneg_lead ~ Stiff_cat + Power + Speed + (1 | Sub)
##    Data: ps_data[ps_data$Transition == "AL2UL", ]
##
## REML criterion at convergence: 6045.3
##
## Scaled residuals:
##     Min      1Q  Median      3Q     Max
## -5.7160 -0.5321  0.1680  0.6705  2.3151
##
## Random effects:
##  Groups   Name        Variance Std.Dev.
##  Sub      (Intercept) 29.22    5.406
##  Residual             24.89    4.989
## Number of obs: 991, groups:  Sub, 13
##
## Fixed effects:
##                Estimate Std. Error       df t value Pr(>|t|)
## (Intercept)     18.4140     1.6556  17.4527  11.122 2.39e-09 ***
## Stiff_catdown1   1.2624     0.4498 971.2987   2.807  0.00511 **
## Stiff_catrec     0.9749     0.4512 971.3099   2.161  0.03096 *
## Stiff_catup1     1.8867     0.4545 971.0135   4.151 3.59e-05 ***
## Powerrec        -2.2205     0.4468 971.1409  -4.970 7.92e-07 ***
## Powerten        -1.3479     0.4468 971.1409  -3.017  0.00262 **
## Powertwenty     -0.6547     0.4468 971.1409  -1.465  0.14313
## Speed          -28.0877     0.4545 971.2076 -61.796  < 2e-16 ***
## ---
## Signif. codes:  0 '***' 0.001 '**' 0.01 '*' 0.05 '.' 0.1 ' ' 1
##
## Correlation of Fixed Effects:
##             (Intr) Stff_ctd1 Stff_c Stff_ctp1 Powrrc Powrtn Pwrtwn
## Stff_ctdwn1 -0.138
## Stiff_catrc -0.140  0.518
## Stiff_catp1 -0.137  0.505     0.504
## Powerrec    -0.130 -0.016    -0.014  0.000
```

```
## Powerten    -0.130 -0.016    -0.014  0.000     0.491
## Powertwenty -0.130 -0.016    -0.014  0.000     0.491  0.491
## Speed       -0.341  0.000     0.006  0.000     0.002  0.002  0.002
```

```
confint(lme.wneg_al2ul_sp2)
```

```
## Computing profile confidence intervals ...
```

```
##                       2.5 %      97.5 %
## .sig01           3.67106528   8.0934784
## .sigma           4.75855885   5.1996750
## (Intercept)     15.10486324  21.7237305
## Stiff_catdown1   0.38303181   2.1415250
## Stiff_catrec     0.09274341   1.8566298
## Stiff_catup1     0.99829323   2.7751508
## Powerrec        -3.09380770  -1.3469733
## Powerten        -2.22127709  -0.4744427
## Powertwenty     -1.52809342   0.2187410
## Speed          -28.97598368 -27.1989413
```

Appendix Table 2: Interaction between stiffness category and power setting on W during UL to AL transition

```
lme.wpos_ul2al_sp <- lmer(Wpos_trail ~ Stiff_cat*Power + Speed + (1|Sub), data = ps_data[ps_data$Transi
summary(lme.wpos_ul2al_sp) #no interaction between stiffness and power
```

```
## Linear mixed model fit by REML. t-tests use Satterthwaite's method [
## lmerModLmerTest]
## Formula: Wpos_trail ~ Stiff_cat * Power + Speed + (1 | Sub)
##    Data: ps_data[ps_data$Transition == "UL2AL", ]
##
## REML criterion at convergence: 5049.6
##
## Scaled residuals:
##     Min      1Q  Median      3Q     Max
## -4.1201 -0.5851  0.0704  0.6319  3.1402
##
## Random effects:
##  Groups   Name        Variance Std.Dev.
##  Sub      (Intercept) 40.299   6.348
##  Residual              9.045   3.007
## Number of obs: 991, groups:  Sub, 13
##
## Fixed effects:
##                              Estimate Std. Error       df t value Pr(>|t|)
## (Intercept)                    0.3793     1.8322  13.9887   0.207   0.8390
## Stiff_catdown1                -0.5475     0.5316 962.0026  -1.030   0.3034
## Stiff_catrec                  -0.6116     0.5316 962.0026  -1.150   0.2503
## Stiff_catup1                  -0.6592     0.5316 962.0026  -1.240   0.2153
## Powerrec                      -0.8456     0.5439 962.0374  -1.555   0.1203
## Powerten                      -1.3932     0.5439 962.0374  -2.562   0.0106 *
## Powertwenty                   -1.3846     0.5439 962.0374  -2.546   0.0111 *
```

```
## Speed                       14.8830     0.2740 962.0541  54.315   <2e-16 ***
## Stiff_catdown1:Powerrec      0.7004     0.7606 962.0204   0.921   0.3573
## Stiff_catrec:Powerrec        0.6912     0.7621 962.0212   0.907   0.3646
## Stiff_catup1:Powerrec        0.9640     0.7676 962.0026   1.256   0.2095
## Stiff_catdown1:Powerten      0.9460     0.7606 962.0204   1.244   0.2139
## Stiff_catrec:Powerten        1.3825     0.7621 962.0212   1.814   0.0700 .
## Stiff_catup1:Powerten        0.2979     0.7676 962.0026   0.388   0.6981
## Stiff_catdown1:Powertwenty   0.5669     0.7606 962.0204   0.745   0.4562
## Stiff_catrec:Powertwenty     0.6759     0.7621 962.0212   0.887   0.3753
## Stiff_catup1:Powertwenty     0.9852     0.7676 962.0026   1.283   0.1997
## ---
## Signif. codes:  0 '***' 0.001 '**' 0.01 '*' 0.05 '.' 0.1 ' ' 1

##
## Correlation matrix not shown by default, as p = 17 > 12.
## Use print(x, correlation=TRUE)  or
##     vcov(x)        if you need it
```

```r
lme.wpos_ul2al_sp2 <- lmer(Wpos_trail ~ Stiff_cat + Power + Speed + (1|Sub), data = ps_data[ps_data$Tran
summary(lme.wpos_ul2al_sp2)
```

```
## Linear mixed model fit by REML. t-tests use Satterthwaite's method [
## lmerModLmerTest]
## Formula: Wpos_trail ~ Stiff_cat + Power + Speed + (1 | Sub)
##    Data: ps_data[ps_data$Transition == "UL2AL", ]
##
## REML criterion at convergence: 5064.4
##
## Scaled residuals:
##     Min      1Q  Median      3Q     Max
## -4.1990 -0.5913  0.0587  0.6454  3.2027
##
## Random effects:
##  Groups   Name        Variance Std.Dev.
##  Sub      (Intercept) 40.276   6.346
##  Residual              9.028   3.005
## Number of obs: 991, groups:  Sub, 13
##
## Fixed effects:
##                 Estimate Std. Error         df t value Pr(>|t|)
## (Intercept)    -0.061171   1.810267  13.347970  -0.034  0.97354
## Stiff_catdown1 -0.005654   0.270905 971.079292  -0.021  0.98335
## Stiff_catrec    0.063363   0.271736 971.082296   0.233  0.81567
## Stiff_catup1   -0.109129   0.273718 971.002609  -0.399  0.69021
## Powerrec       -0.257236   0.269100 971.036789  -0.956  0.33935
## Powerten       -0.723358   0.269100 971.036789  -2.688  0.00731 **
## Powertwenty    -0.829930   0.269100 971.036789  -3.084  0.00210 **
## Speed          14.881934   0.273756 971.054438  54.362  < 2e-16 ***
## ---
## Signif. codes:  0 '***' 0.001 '**' 0.01 '*' 0.05 '.' 0.1 ' ' 1
##
## Correlation of Fixed Effects:
##             (Intr) Stff_ctd1 Stff_c Stff_ctp1 Powrrc Powrtn Pwrtwn
```

```
## Stff_ctdwn1 -0.076
## Stiff_catrc -0.077  0.518
## Stiff_catp1 -0.076  0.505     0.504
## Powerrec    -0.072 -0.016    -0.014  0.000
## Powerten    -0.072 -0.016    -0.014  0.000     0.491
## Powertwenty -0.072 -0.016    -0.014  0.000     0.491  0.491
## Speed       -0.188  0.000     0.006  0.000     0.002  0.002  0.002
```

```r
confint(lme.wpos_ul2al_sp2)
```

```
## Computing profile confidence intervals ...
```

```
##                      2.5 %     97.5 %
## .sig01           4.3292653  9.4807790
## .sigma           2.8659623  3.1316374
## (Intercept)     -3.7192704  3.5971496
## Stiff_catdown1 -0.5353267  0.5238336
## Stiff_catrec   -0.4679256  0.5944841
## Stiff_catup1   -0.6442074  0.4259502
## Powerrec       -0.7832225  0.2688810
## Powerten       -1.2493450 -0.1972414
## Powertwenty    -1.3559164 -0.3038128
## Speed          14.3468656 15.4171748
```

```r
lme.wneg_ul2al_sp <- lmer(Wneg_lead ~ Stiff_cat*Power + Speed + (1|Sub), data = ps_data[ps_data$Transit[illegible]
summary(lme.wneg_ul2al_sp)
```

```
## Linear mixed model fit by REML. t-tests use Satterthwaite's method [
## lmerModLmerTest]
## Formula: Wneg_lead ~ Stiff_cat * Power + Speed + (1 | Sub)
##    Data: ps_data[ps_data$Transition == "UL2AL", ]
##
## REML criterion at convergence: 5299
##
## Scaled residuals:
##     Min      1Q  Median      3Q     Max
## -8.5351 -0.5332  0.1462  0.6343  2.4774
##
## Random effects:
##  Groups   Name        Variance Std.Dev.
##  Sub      (Intercept) 18.05    4.248
##  Residual             11.84    3.441
## Number of obs: 991, groups:  Sub, 13
##
## Fixed effects:
##                              Estimate Std. Error        df t value Pr(>|t|)
## (Intercept)                  13.71298    1.31312  18.18202  10.443 4.12e-09 ***
## Stiff_catdown1               -0.02484    0.60823 961.99924  -0.041   0.9674
## Stiff_catrec                 -0.56922    0.60823 961.99924  -0.936   0.3496
## Stiff_catup1                  0.09438    0.60823 961.99924   0.155   0.8767
## Powerrec                     -2.95780    0.62223 962.09971  -4.754 2.30e-06 ***
## Powerten                     -3.82220    0.62223 962.09971  -6.143 1.19e-09 ***
```

```
## Powertwenty                 -3.46695    0.62223 962.09971  -5.572 3.27e-08 ***
## Speed                      -18.43412    0.31347 962.14825 -58.806  < 2e-16 ***
## Stiff_catdown1:Powerrec      0.29967    0.87012 962.05067   0.344   0.7306
## Stiff_catrec:Powerrec        0.81560    0.87186 962.05299   0.935   0.3498
## Stiff_catup1:Powerrec        0.33766    0.87820 961.99924   0.384   0.7007
## Stiff_catdown1:Powerten      1.78314    0.87012 962.05067   2.049   0.0407 *
## Stiff_catrec:Powerten        1.37461    0.87186 962.05299   1.577   0.1152
## Stiff_catup1:Powerten        0.25562    0.87820 961.99924   0.291   0.7711
## Stiff_catdown1:Powertwenty   0.79851    0.87012 962.05067   0.918   0.3590
## Stiff_catrec:Powertwenty     1.26936    0.87186 962.05299   1.456   0.1457
## Stiff_catup1:Powertwenty     0.18817    0.87820 961.99924   0.214   0.8304
## ---
## Signif. codes:  0 '***' 0.001 '**' 0.01 '*' 0.05 '.' 0.1 ' ' 1

##
## Correlation matrix not shown by default, as p = 17 > 12.
## Use print(x, correlation=TRUE)  or
##     vcov(x)        if you need it
```

```
confint(lme.wneg_ul2al_sp)
```

```
## Computing profile confidence intervals ...
```

```
##                                   2.5 %      97.5 %
## .sig01                       2.88826410   6.3563264
## .sigma                       3.26658160   3.5693952
## (Intercept)                 11.09418142  16.3321574
## Stiff_catdown1              -1.20831973   1.1586322
## Stiff_catrec                -1.75269473   0.6142572
## Stiff_catup1                -1.08910098   1.2778510
## Powerrec                    -4.16888716  -1.7474514
## Powerten                    -5.03329393  -2.6118582
## Powertwenty                 -4.67803970  -2.2566040
## Speed                      -19.04398955 -17.8240873
## Stiff_catdown1:Powerrec     -1.39301595   1.9931046
## Stiff_catrec:Powerrec       -0.88049265   2.5123939
## Stiff_catup1:Powerrec       -1.37112049   2.0464383
## Stiff_catdown1:Powerten      0.09045333   3.4765739
## Stiff_catrec:Powerten       -0.32148269   3.0714038
## Stiff_catup1:Powerten       -1.45315439   1.9644044
## Stiff_catdown1:Powertwenty  -0.89417591   2.4919447
## Stiff_catrec:Powertwenty    -0.42673693   2.9661496
## Stiff_catup1:Powertwenty    -1.52061201   1.8969468
```

Table 6: Effect of stiffness category on EFLR

```
eflr_passive <- lmer(EFLR ~ Stiff_cat*Speed + Leg*Speed + Stiff_cat*Leg + (1|Sub), data = ps_data_passi
summary(eflr_passive)
```

```
## Linear mixed model fit by REML. t-tests use Satterthwaite's method [
## lmerModLmerTest]
## Formula: EFLR ~ Stiff_cat * Speed + Leg * Speed + Stiff_cat * Leg + (1 |
```

```
##     Sub)
##    Data: ps_data_passive
##
## REML criterion at convergence: -1456.9
##
## Scaled residuals:
##     Min      1Q  Median      3Q     Max
## -2.3698 -0.7027  0.0105  0.6286  3.3620
##
## Random effects:
##  Groups   Name        Variance Std.Dev.
##  Sub      (Intercept) 0.005597 0.07481
##  Residual             0.002583 0.05082
## Number of obs: 512, groups:  Sub, 13
##
## Fixed effects:
##                       Estimate Std. Error         df t value Pr(>|t|)
## (Intercept)           0.746712   0.028065  38.621931  26.607  < 2e-16 ***
## Stiff_catdown1        0.017328   0.024229 487.002639   0.715 0.474854
## Stiff_catrec          0.028690   0.024229 487.002639   1.184 0.236949
## Stiff_catup1          0.039838   0.024229 487.002639   1.644 0.100775
## Speed                 0.114312   0.014331 487.023151   7.976 1.09e-14 ***
## LegUL                -0.065716   0.018272 487.002640  -3.596 0.000356 ***
## Stiff_catdown1:Speed -0.008000   0.018115 487.002639  -0.442 0.658971
## Stiff_catrec:Speed   -0.010278   0.018115 487.002639  -0.567 0.570697
## Stiff_catup1:Speed   -0.015215   0.018115 487.002639  -0.840 0.401348
## Speed:LegUL           0.011985   0.012809 487.002640   0.936 0.349911
## Stiff_catdown1:LegUL -0.009141   0.012705 487.002640  -0.719 0.472220
## Stiff_catrec:LegUL   -0.019953   0.012705 487.002640  -1.570 0.116960
## Stiff_catup1:LegUL   -0.026922   0.012705 487.002640  -2.119 0.034601 *
## ---
## Signif. codes:  0 '***' 0.001 '**' 0.01 '*' 0.05 '.' 0.1 ' ' 1
```

```
##
## Correlation matrix not shown by default, as p = 13 > 12.
## Use print(x, correlation=TRUE)  or
##     vcov(x)        if you need it
```

```
eflr_passive2 <- lmer(EFLR ~ Stiff_cat*Leg + Speed + (1|Sub), data = ps_data_passive)
summary(eflr_passive2)
```

```
## Linear mixed model fit by REML. t-tests use Satterthwaite's method [
## lmerModLmerTest]
## Formula: EFLR ~ Stiff_cat * Leg + Speed + (1 | Sub)
##    Data: ps_data_passive
##
## REML criterion at convergence: -1481.4
##
## Scaled residuals:
##     Min      1Q  Median      3Q     Max
## -2.5149 -0.7005  0.0222  0.6273  3.3684
##
## Random effects:
```

```
##  Groups   Name        Variance Std.Dev.
##  Sub      (Intercept) 0.005597 0.07481
##  Residual             0.002570 0.05070
## Number of obs: 512, groups:  Sub, 13
##
## Fixed effects:
##                        Estimate Std. Error         df t value Pr(>|t|)
## (Intercept)            0.749669   0.023108  18.015436  32.442  < 2e-16 ***
## Stiff_catdown1         0.007391   0.008962 491.002626   0.825   0.4100
## Stiff_catrec           0.015922   0.008962 491.002626   1.777   0.0763 .
## Stiff_catup1           0.020937   0.008962 491.002626   2.336   0.0199 *
## LegUL                 -0.050828   0.008962 491.002626  -5.671 2.42e-08 ***
## Speed                  0.111931   0.006412 491.104762  17.455  < 2e-16 ***
## Stiff_catdown1:LegUL  -0.009141   0.012674 491.002626  -0.721   0.4711
## Stiff_catrec:LegUL    -0.019953   0.012674 491.002626  -1.574   0.1161
## Stiff_catup1:LegUL    -0.026922   0.012674 491.002626  -2.124   0.0342 *
## ---
## Signif. codes:  0 '***' 0.001 '**' 0.01 '*' 0.05 '.' 0.1 ' ' 1
##
## Correlation of Fixed Effects:
##                 (Intr) Stff_ctd1 Stff_c Stff_ctp1 LegUL  Speed  Stff_ctd1:LUL
## Stff_ctdwn1     -0.194
## Stiff_catrc     -0.194  0.500
## Stiff_catp1     -0.194  0.500     0.500
## LegUL           -0.194  0.500     0.500  0.500
## Speed           -0.344  0.000     0.000  0.000     0.000
## Stff_ctd1:LUL    0.137 -0.707    -0.354 -0.354    -0.707  0.000
## Stff_ct:LUL      0.137 -0.354    -0.707 -0.354    -0.707  0.000  0.500
## Stff_ctp1:LUL    0.137 -0.354    -0.354 -0.707    -0.707  0.000  0.500
##                 S_:LUL
## Stff_ctdwn1
## Stiff_catrc
## Stiff_catp1
## LegUL
## Speed
## Stff_ctd1:LUL
## Stff_ct:LUL
## Stff_ctp1:LUL  0.500
```

```r
confint(eflr_passive2)
```

```
## Computing profile confidence intervals ...
```

```
##                             2.5 %       97.5 %
## .sig01                0.050789092  0.112034621
## .sigma                0.047323800  0.053578569
## (Intercept)           0.703554145  0.795767269
## Stiff_catdown1       -0.010067090  0.024848340
## Stiff_catrec         -0.001535840  0.033379590
## Stiff_catup1          0.003479785  0.038395215
## LegUL                -0.068285840 -0.033370410
## Speed                 0.099436308  0.124418477
## Stiff_catdown1:LegUL -0.033829562  0.015548312
```

```
## Stiff_catrec:LegUL   -0.044642062  0.004735812
## Stiff_catup1:LegUL   -0.051610812 -0.002232938
```

Table 7: Effect of power setting on EFLR

```r
eflr_krec <- lmer(EFLR ~ Power*Speed + Leg*Speed + Power*Leg + (1|Sub), data = ps_data_krec)
summary(eflr_krec) #no interactions
```

```
## Linear mixed model fit by REML. t-tests use Satterthwaite's method [
## lmerModLmerTest]
## Formula: EFLR ~ Power * Speed + Leg * Speed + Power * Leg + (1 | Sub)
##    Data: ps_data_krec
##
## REML criterion at convergence: -1488
##
## Scaled residuals:
##     Min      1Q  Median      3Q     Max
## -2.9196 -0.6245  0.0104  0.6162  4.7078
##
## Random effects:
##  Groups   Name        Variance Std.Dev.
##  Sub      (Intercept) 0.006159 0.07848
##  Residual             0.002325 0.04822
## Number of obs: 506, groups:  Sub, 13
##
## Fixed effects:
##                      Estimate Std. Error         df t value Pr(>|t|)
## (Intercept)         7.770e-01  2.822e-02  3.292e+01  27.528  < 2e-16 ***
## Powerrec            3.717e-04  2.311e-02  4.810e+02   0.016    0.987
## Powerten            2.142e-02  2.311e-02  4.810e+02   0.927    0.354
## Powertwenty         2.903e-02  2.311e-02  4.810e+02   1.256    0.210
## Speed               1.025e-01  1.363e-02  4.810e+02   7.517 2.76e-13 ***
## LegUL              -8.571e-02  1.750e-02  4.810e+02  -4.897 1.33e-06 ***
## Powerrec:Speed      1.969e-03  1.734e-02  4.810e+02   0.114    0.910
## Powerten:Speed     -8.401e-03  1.734e-02  4.810e+02  -0.484    0.628
## Powertwenty:Speed -1.063e-02  1.734e-02  4.810e+02  -0.613    0.540
## Speed:LegUL         1.202e-02  1.231e-02  4.810e+02   0.976    0.329
## Powerrec:LegUL      6.719e-03  1.210e-02  4.810e+02   0.555    0.579
## Powerten:LegUL      8.656e-03  1.210e-02  4.810e+02   0.715    0.475
## Powertwenty:LegUL   3.275e-03  1.210e-02  4.810e+02   0.271    0.787
## ---
## Signif. codes:  0 '***' 0.001 '**' 0.01 '*' 0.05 '.' 0.1 ' ' 1

##
## Correlation matrix not shown by default, as p = 13 > 12.
## Use print(x, correlation=TRUE)  or
##     vcov(x)        if you need it
```

```r
eflr_krec2 <- lmer(EFLR ~ Power + Leg + Speed + (1|Sub), data = ps_data_krec)
summary(eflr_krec2)
```

```
## Linear mixed model fit by REML. t-tests use Satterthwaite's method [
```

```
## lmerModLmerTest]
## Formula: EFLR ~ Power + Leg + Speed + (1 | Sub)
##    Data: ps_data_krec
##
## REML criterion at convergence: -1533.8
##
## Scaled residuals:
##     Min      1Q  Median      3Q     Max
## -2.8862 -0.5805 -0.0062  0.6295  4.6925
##
## Random effects:
##  Groups   Name        Variance Std.Dev.
##  Sub      (Intercept) 0.006161 0.07849
##  Residual             0.002302 0.04798
## Number of obs: 506, groups:  Sub, 13
##
## Fixed effects:
##              Estimate Std. Error         df t value Pr(>|t|)
## (Intercept)   0.772468   0.023555  16.125705  32.795 3.43e-16 ***
## Powerrec      0.006129   0.006023 488.007553   1.018   0.3094
## Powerten      0.015351   0.006023 488.007553   2.549   0.0111 *
## Powertwenty   0.017518   0.006023 488.007553   2.908   0.0038 **
## LegUL        -0.066209   0.004266 488.002813 -15.520  < 2e-16 ***
## Speed         0.104251   0.006157 488.120944  16.933  < 2e-16 ***
## ---
## Signif. codes:  0 '***' 0.001 '**' 0.01 '*' 0.05 '.' 0.1 ' ' 1
##
## Correlation of Fixed Effects:
##             (Intr) Powrrc Powrtn Pwrtwn LegUL
## Powerrec    -0.130
## Powerten    -0.130  0.496
## Powertwenty -0.130  0.496  0.496
## LegUL       -0.091  0.000  0.000  0.000
## Speed       -0.324  0.009  0.009  0.009  0.000
```

```
confint(eflr_krec2)
```

```
## Computing profile confidence intervals ...
```

```
##                    2.5 %      97.5 %
## .sig01       0.053345195  0.11747755
## .sigma       0.044906549  0.05088016
## (Intercept)  0.725239999  0.81966857
## Powerrec    -0.005640172  0.01789556
## Powerten     0.003582050  0.02711778
## Powertwenty  0.005748717  0.02928444
## LegUL       -0.074544651 -0.05787432
## Speed        0.092216630  0.11627545
```

Appendix Table 3: Interaction of stiffness category and power setting on EFLR

```r
eflr_ps <- lmer(EFLR ~ Stiff_cat*Power + Leg + Speed + (1|Sub), data = ps_data)
summary(eflr_ps)
```

```
## Linear mixed model fit by REML. t-tests use Satterthwaite's method [
## lmerModLmerTest]
## Formula: EFLR ~ Stiff_cat * Power + Leg + Speed + (1 | Sub)
##    Data: ps_data
##
## REML criterion at convergence: -5944.6
##
## Scaled residuals:
##     Min      1Q  Median      3Q     Max
## -3.4554 -0.6552 -0.0121  0.6827  4.7642
##
## Random effects:
##  Groups   Name        Variance Std.Dev.
##  Sub      (Intercept) 0.005435 0.07372
##  Residual             0.002619 0.05118
## Number of obs: 1982, groups:  Sub, 13
##
## Fixed effects:
##                              Estimate Std. Error         df t value Pr(>|t|)
## (Intercept)                 7.625e-01  2.137e-02  1.422e+01  35.687 2.53e-15
## Stiff_catdown1              2.820e-03  6.397e-03  1.952e+03   0.441   0.6593
## Stiff_catrec                5.945e-03  6.397e-03  1.952e+03   0.929   0.3528
## Stiff_catup1                7.477e-03  6.397e-03  1.952e+03   1.169   0.2426
## Powerrec                   -3.539e-03  6.544e-03  1.952e+03  -0.541   0.5887
## Powerten                    1.681e-03  6.544e-03  1.952e+03   0.257   0.7973
## Powertwenty                 8.825e-03  6.544e-03  1.952e+03   1.349   0.1776
## LegUL                      -5.776e-02  2.299e-03  1.952e+03 -25.122  < 2e-16
## Speed                       1.042e-01  3.297e-03  1.952e+03  31.615  < 2e-16
## Stiff_catdown1:Powerrec    -4.984e-03  9.151e-03  1.952e+03  -0.545   0.5861
## Stiff_catrec:Powerrec       9.814e-03  9.170e-03  1.952e+03   1.070   0.2846
## Stiff_catup1:Powerrec       2.120e-02  9.236e-03  1.952e+03   2.295   0.0218
## Stiff_catdown1:Powerten    -1.361e-03  9.151e-03  1.952e+03  -0.149   0.8818
## Stiff_catrec:Powerten       1.382e-02  9.170e-03  1.952e+03   1.507   0.1321
## Stiff_catup1:Powerten       1.778e-02  9.236e-03  1.952e+03   1.925   0.0544
## Stiff_catdown1:Powertwenty -6.817e-03  9.151e-03  1.952e+03  -0.745   0.4564
## Stiff_catrec:Powertwenty    8.838e-03  9.170e-03  1.952e+03   0.964   0.3352
## Stiff_catup1:Powertwenty    1.726e-02  9.236e-03  1.952e+03   1.869   0.0618
##
## (Intercept)                ***
## Stiff_catdown1
## Stiff_catrec
## Stiff_catup1
## Powerrec
## Powerten
## Powertwenty
## LegUL                      ***
## Speed                      ***
## Stiff_catdown1:Powerrec
## Stiff_catrec:Powerrec
## Stiff_catup1:Powerrec      *
```

```
## Stiff_catdown1:Powerten
## Stiff_catrec:Powerten
## Stiff_catup1:Powerten      .
## Stiff_catdown1:Powertwenty
## Stiff_catrec:Powertwenty
## Stiff_catup1:Powertwenty   .
## ---
## Signif. codes:  0 '***' 0.001 '**' 0.01 '*' 0.05 '.' 0.1 ' ' 1
```

```
##
## Correlation matrix not shown by default, as p = 18 > 12.
## Use print(x, correlation=TRUE)  or
##     vcov(x)        if you need it
```

```
confint(eflr_ps)
```

```
## Computing profile confidence intervals ...
```

```
##                                    2.5 %       97.5 %
## .sig01                      0.0502852828  0.110134740
## .sigma                      0.0494028133  0.052587560
## (Intercept)                 0.7194773844  0.805587627
## Stiff_catdown1             -0.0096692177  0.015309843
## Stiff_catrec               -0.0065442177  0.018434843
## Stiff_catup1               -0.0050129677  0.019966093
## Powerrec                   -0.0163170876  0.009237239
## Powerten                   -0.0110967486  0.014457578
## Powertwenty                -0.0039526808  0.021601645
## LegUL                      -0.0622444369 -0.053267168
## Speed                       0.0977950940  0.110669382
## Stiff_catdown1:Powerrec    -0.0228509208  0.012883895
## Stiff_catrec:Powerrec      -0.0080891279  0.027717120
## Stiff_catup1:Powerrec       0.0031682098  0.039234597
## Stiff_catdown1:Powerten    -0.0192275097  0.016507306
## Stiff_catrec:Powerten      -0.0040872447  0.031719003
## Stiff_catup1:Powerten      -0.0002555191  0.035810869
## Stiff_catdown1:Powertwenty -0.0246840775  0.011050738
## Stiff_catrec:Powertwenty   -0.0090646458  0.026741602
## Stiff_catup1:Powertwenty   -0.0007724682  0.035293919
```

Averages

```
#Wpos

#average AL Wpos with krec
avg_wpos_krec <- ps_data_krec[ps_data_krec$Transition == "AL2UL",] %>%
  group_by(Power, Speed) %>%
  summarise(
    w_trail_avg = mean(Wpos_trail)
  )
```

```
## `summarise()` has grouped output by 'Power'. You can override using the
## `.groups` argument.
```

```r
#average UL Wpos with krec
avg_wpos_krec_ul <- ps_data_krec[ps_data_krec$Transition == "UL2AL",] %>%
  group_by(Speed) %>%
  summarise(
    w_trail_avg = mean(Wpos_trail)
  )

#EFLR

#Average EFLR grouped by leg
avg_eflr_l <- ps_data_passive %>%
  group_by(Leg) %>%
  summarise(
    eflr_avg = mean(EFLR)
  )

#Average EFLR grouped by speed and leg
avg_eflr_sl <- ps_data_passive %>%
  group_by(Speed, Leg) %>%
  summarise(
    eflr_avg = mean(EFLR)
  )
```

```
## `summarise()` has grouped output by 'Speed'. You can override using the
## `.groups` argument.
```

```r
#Average EFLR grouped by stiffness category and leg
avg_eflr_kl <- ps_data_passive %>%
  group_by(Stiff_cat, Leg) %>%
  summarise(
    eflr_avg = mean(EFLR)
  )
```

```
## `summarise()` has grouped output by 'Stiff_cat'. You can override using the
## `.groups` argument.
```

```r
#Average EFLR grouped by speed and leg
avg_eflr_sl_pow <- ps_data_krec %>%
  group_by(Speed, Leg) %>%
  summarise(
    eflr_avg = mean(EFLR)
  )
```

```
## `summarise()` has grouped output by 'Speed'. You can override using the
## `.groups` argument.
```

```r
#Average EFLR grouped by power and leg
avg_eflr_pl <- ps_data_krec %>%
  group_by(Power, Leg) %>%
  summarise(
    eflr_avg = mean(EFLR)
  )
```

```
## 'summarise()' has grouped output by 'Power'. You can override using the
## '.groups' argument.
```

Manuscript Figures

Set up color palettes

```
bl <- c("#96d5cd", "#0caac9", "#07669e", "#002c71")
rd <- c("#f9989b", "#de4c86", "#7d3392", "#461e63")
```

Effect of Stiffness Category

Figure 2b

```
#Positive AL work vs. Speed (Grouped by Stiffness Category, without BiOM)

pos_work_stiff <- ggplot(data = ps_data_passive, aes(x = Speed, y = Wpos_trail, col = Trans.Stiff, shape
  stat_summary(geom="errorbar", fun.data = mean_se, width = 0.2, position=position_dodge(width=0.15)) +
  stat_summary(geom="point", fun = mean, size = 4, position = position_dodge(width=0.15), fill = "white
  labs(x = "Speed (m/s)", y = "Work (J)")+
  scale_x_continuous(limits = c(0.625, 1.975), breaks = c(0.75, 1.00, 1.25, 1.50, 1.75))+
  expand_limits(y= c(0,35))+
  scale_shape_manual(values = c(22, 21, 24, 23, 15, 16, 17, 18))+
  scale_color_manual(values = c(rd,rd)) +
  theme_classic()

pos_work_stiff
```

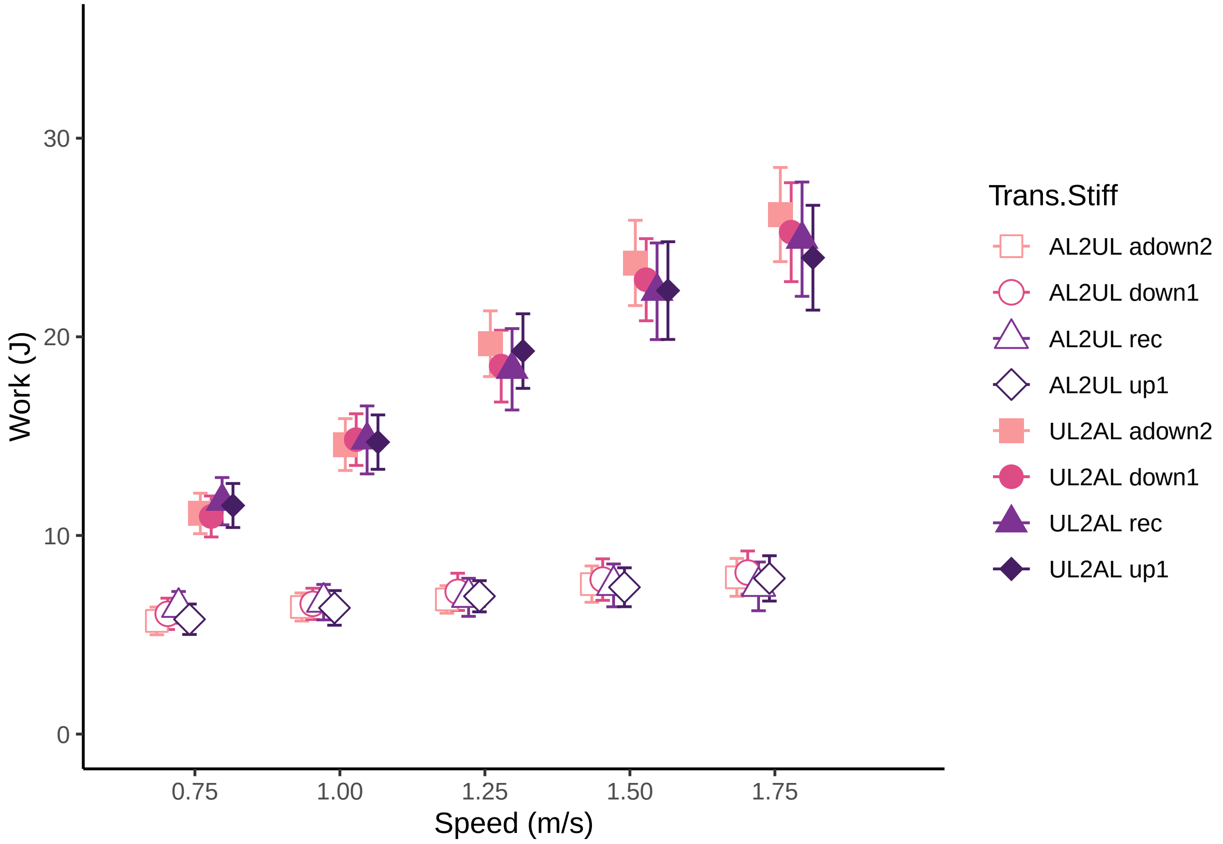

Figure 2c

```
#Negative UL work vs. Speed (Grouped by Stiffness Category, without BiOM)

neg_work_stiff <- ggplot(data = ps_data_passive, aes(x = Speed, y = Wneg_lead, col = Trans.Stiff, shape
  stat_summary(geom="errorbar", fun.data = mean_se, width = 0.2, position=position_dodge(width=0.15)) +
  stat_summary(geom="point", fun = mean, size = 4, position = position_dodge(width=0.15), fill = "white
  labs(x = "Speed (m/s)", y = "Work (J)")+
  scale_x_continuous(limits = c(0.625, 1.975), breaks = c(0.75, 1.00, 1.25, 1.50, 1.75), position = "top
  expand_limits(y= c(-35,0))+
  scale_shape_manual(values = c(15, 16, 17, 18, 22, 21, 24, 23))+
  scale_color_manual(values = c(rd,rd)) +
  theme_classic()

neg_work_stiff
```

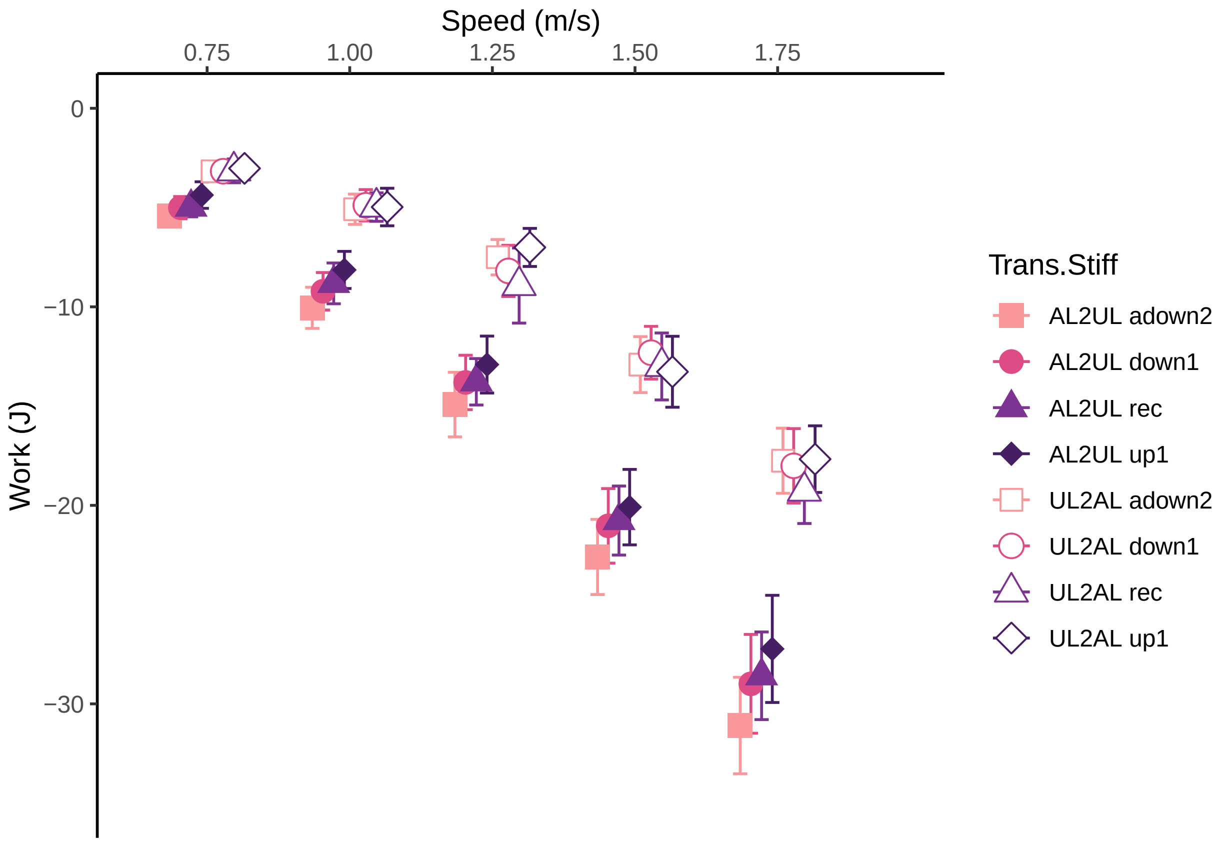

Combined Figure 2

```
fig313 <- grid.arrange(pos_work_stiff + theme(legend.position = "none"), neg_work_stiff + theme(legend.p
```

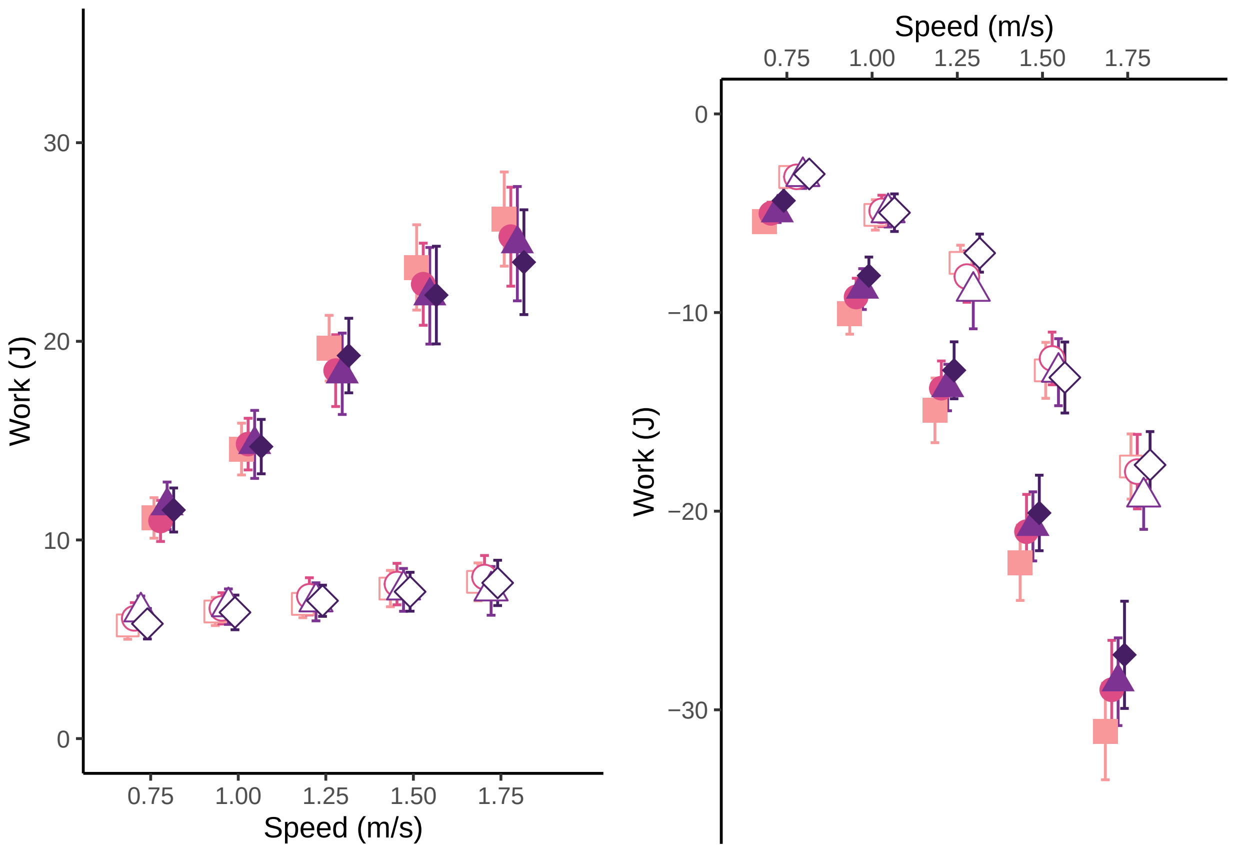


```
fig313
```

```
## TableGrob (1 x 2) "arrange": 2 grobs
##   z     cells    name           grob
## 1 1 (1-1,1-1) arrange gtable[layout]
## 2 2 (1-1,2-2) arrange gtable[layout]
```

```
ggsave("fig313.pdf", plot = fig313, device = "pdf", useDingbats = FALSE, width = 6.7, height = 3.2, uni
```

Effect of Power Setting

Figure 3b

```
#Positive AL work vs. Speed (Grouped by Power setting, recommended stiffness)

pos_work_power <- ggplot(data = ps_data_krec, aes(x = Speed, y = Wpos_trail, col = Trans.Pow, shape = T
  #geom_point(position = position_dodge(width=0.15), alpha = 0.35)+
  stat_summary(geom="errorbar", fun.data = mean_se, width = 0.2, position=position_dodge(width=0.15)) +
  stat_summary(geom="point", fun = mean, size = 4, position = position_dodge(width=0.15), fill = "white
  labs(x = "Speed (m/s)", y = "Work (J)")+
  scale_x_continuous(limits = c(0.625, 1.975), breaks = c(0.75, 1.00, 1.25, 1.50, 1.75))+
  expand_limits(y= c(0,40))+
  scale_shape_manual(values = c(22, 21, 24, 23, 15, 16, 17, 18))+
  scale_color_manual(values = c(bl,bl)) +
  theme_classic()
```

```
pos_work_power
```

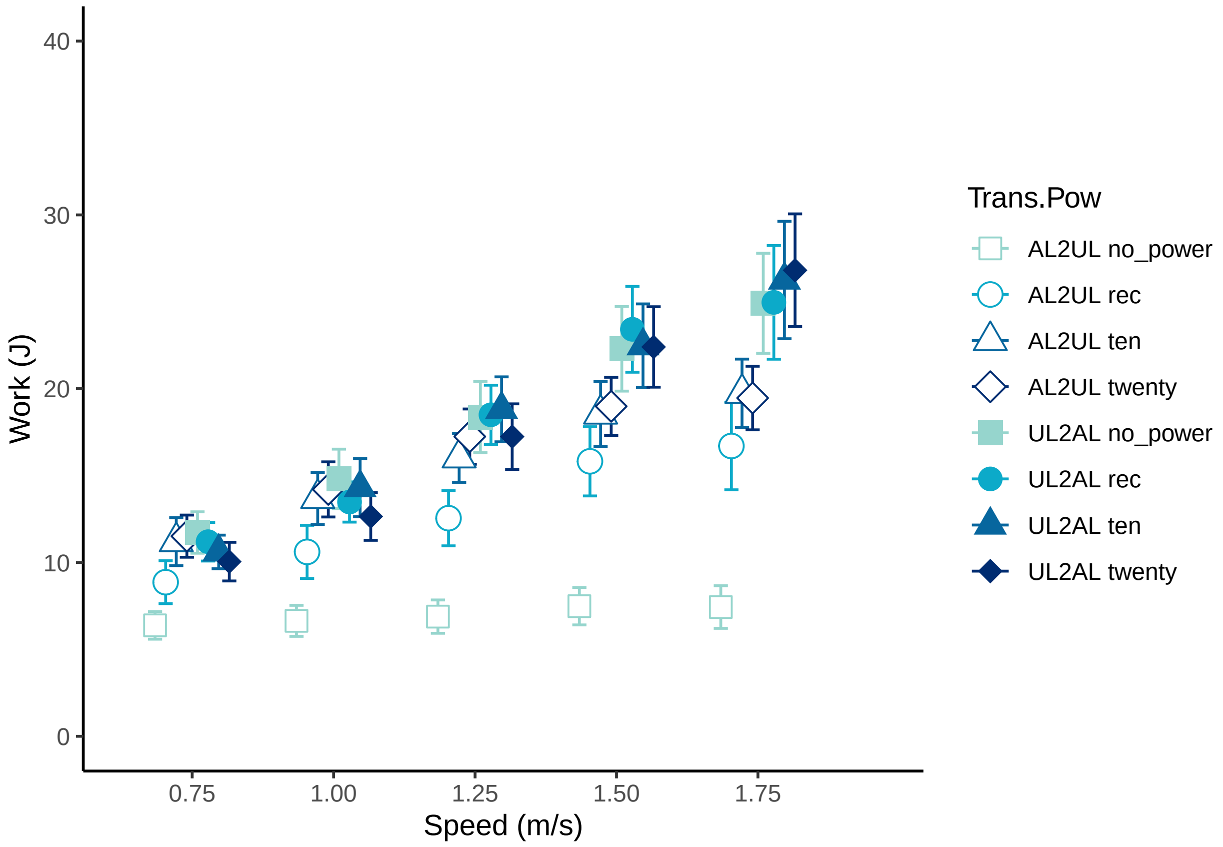

Figure 3c

```
#Negative UL work vs. Speed (Grouped by Power setting, recommended stiffness)

neg_work_power <- ggplot(data = ps_data_krec, aes(x = Speed, y = Wneg_lead, col = Trans.Pow, shape = Tra
  stat_summary(geom="errorbar", fun.data = mean_se, width = 0.2, position=position_dodge(width=0.15)) +
  stat_summary(geom="point", fun = mean, size = 4, position = position_dodge(width=0.15), fill = "white
  labs(x = "Speed (m/s)", y = "Work (J)")+
  scale_x_continuous(limits = c(0.625, 1.975), breaks = c(0.75, 1.00, 1.25, 1.50, 1.75), position = "top
  expand_limits(y= c(-40,0))+
  scale_shape_manual(values = c(15, 16, 17, 18, 22, 21, 24, 23))+
  scale_color_manual(values = c(bl,bl)) +
  theme_classic()

neg_work_power
```

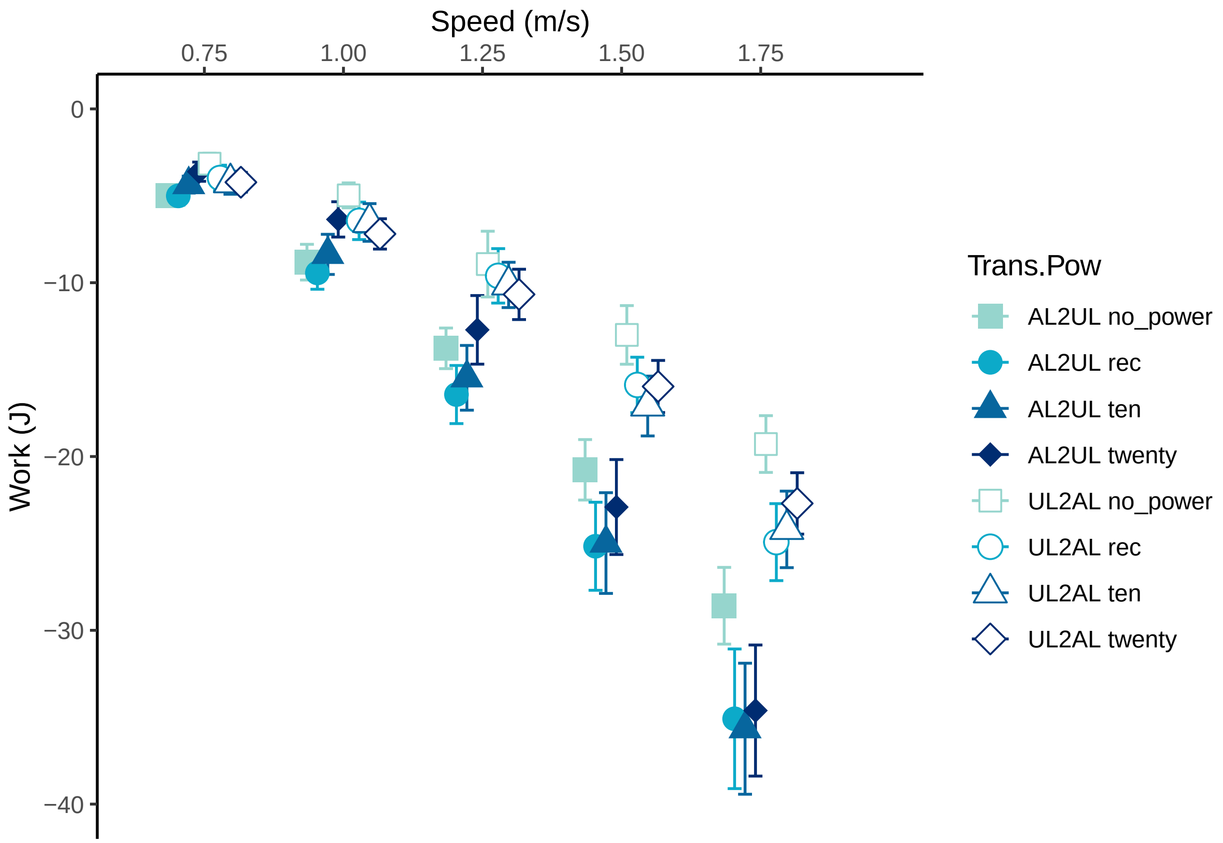

Combined Figure 3

```
fig315 <- grid.arrange(pos_work_power + theme(legend.position = "none"), neg_work_power + theme(legend.p
```

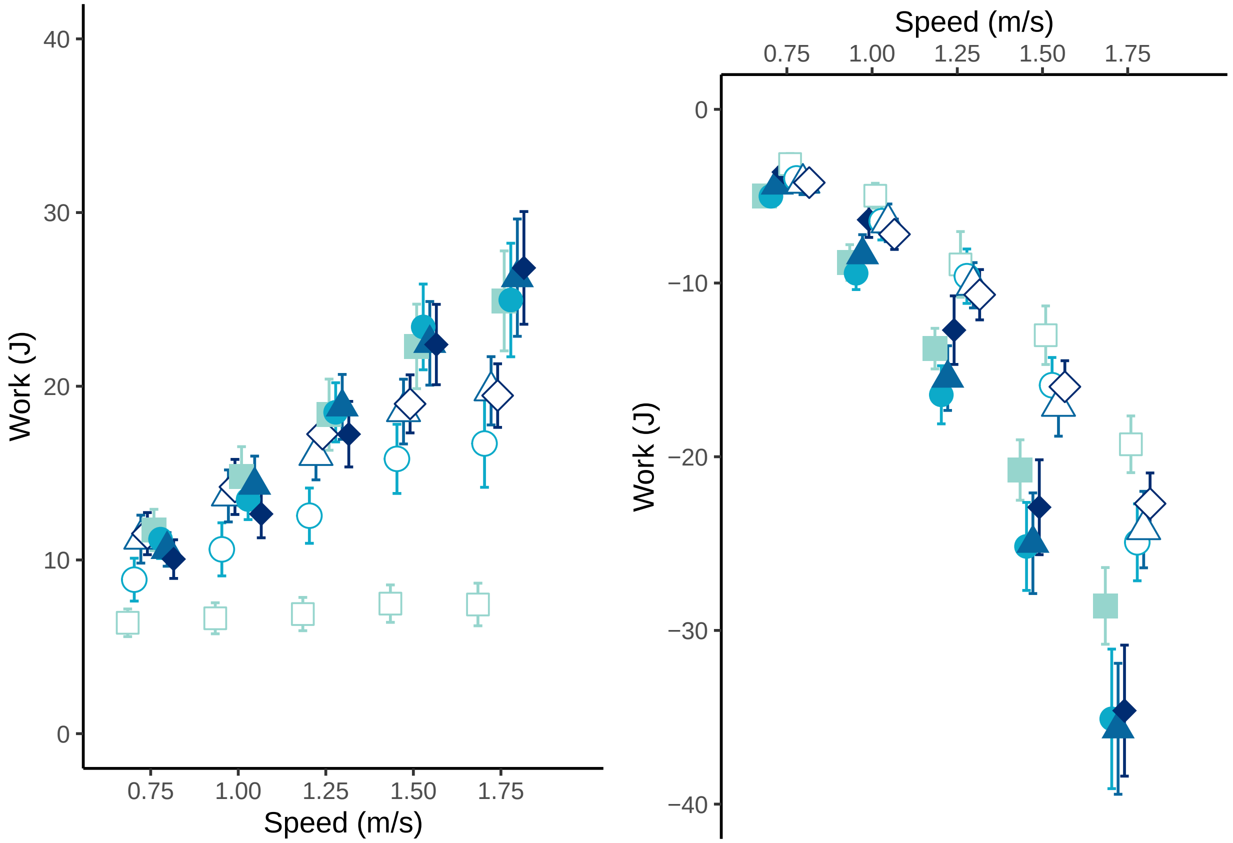


```
fig315
```

```
## TableGrob (1 x 2) "arrange": 2 grobs
##   z     cells    name           grob
## 1 1 (1-1,1-1) arrange gtable[layout]
## 2 2 (1-1,2-2) arrange gtable[layout]
```

```
ggsave("fig315.pdf", plot = fig315, device = "pdf", useDingbats = FALSE, width = 6.7, height = 3.2, uni
```

Roll-over Shape (EFLR) Figure

Figure 4a

```
#EFLR (Grouped by Stiffness Category and Leg, without BiOM)
eflr_stiff <- ggplot(data = ps_data_passive, aes(x = Speed, y = EFLR, col = Leg.Stiff, shape = Leg.Stif
  #geom_jitter(alpha = 0.3)+
  stat_summary(geom="errorbar", fun.data = mean_se, width = 0.2, position=position_dodge(width=0.15)) +
  stat_summary(geom="point", fun = mean, size = 4, position = position_dodge(width=0.15), fill = "white
  labs(x = "Speed (m/s)", y = "EFLR")+
  scale_x_continuous(limits = c(0.625, 1.975), breaks = c(0.75, 1.00, 1.25, 1.50, 1.75))+
  expand_limits(y= c(0,1))+
  scale_shape_manual(values = c(22, 21, 24, 23, 15, 16, 17, 18))+
  scale_color_manual(values = c(rd, rd)) +
  theme_classic()

eflr_stiff
```

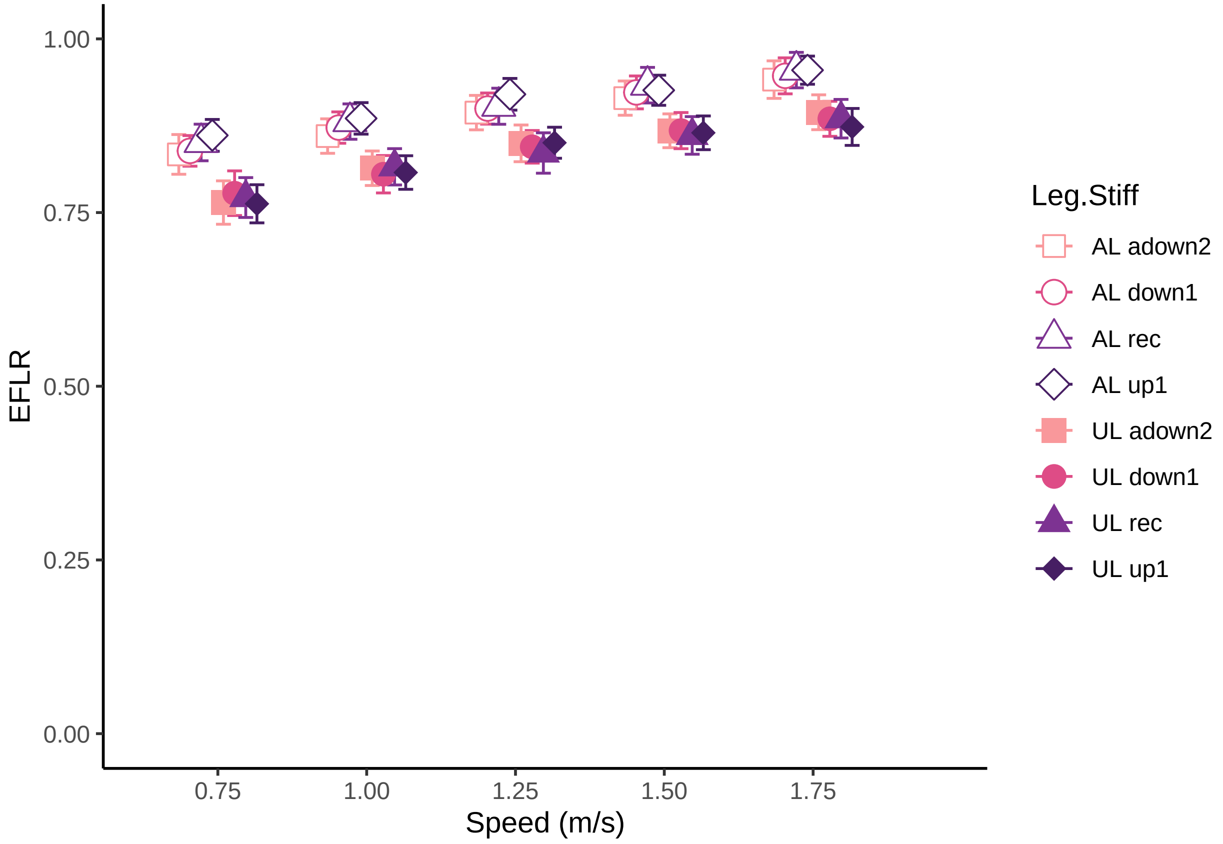

Figure 4b

```
#EFLR (Grouped by Power Setting and Leg, recommended category)
eflr_power <- ggplot(data = ps_data_krec, aes(x = Speed, y = EFLR, col = Leg.Pow, shape = Leg.Pow))+
  #geom_jitter(alpha = 0.3)+
  stat_summary(geom="errorbar", fun.data = mean_se, width = 0.2, position=position_dodge(width=0.15)) +
  stat_summary(geom="point", fun = mean, size = 4, position = position_dodge(width=0.15), fill = "white
  labs(x = "Speed (m/s)", y = "EFLR")+
  scale_x_continuous(limits = c(0.625, 1.975), breaks = c(0.75, 1.00, 1.25, 1.50, 1.75))+
  expand_limits(y= c(0,1))+
  scale_shape_manual(values = c(22, 21, 24, 23, 15, 16, 17, 18))+
  scale_color_manual(values = c(bl, bl)) +
  theme_classic()

eflr_power
```

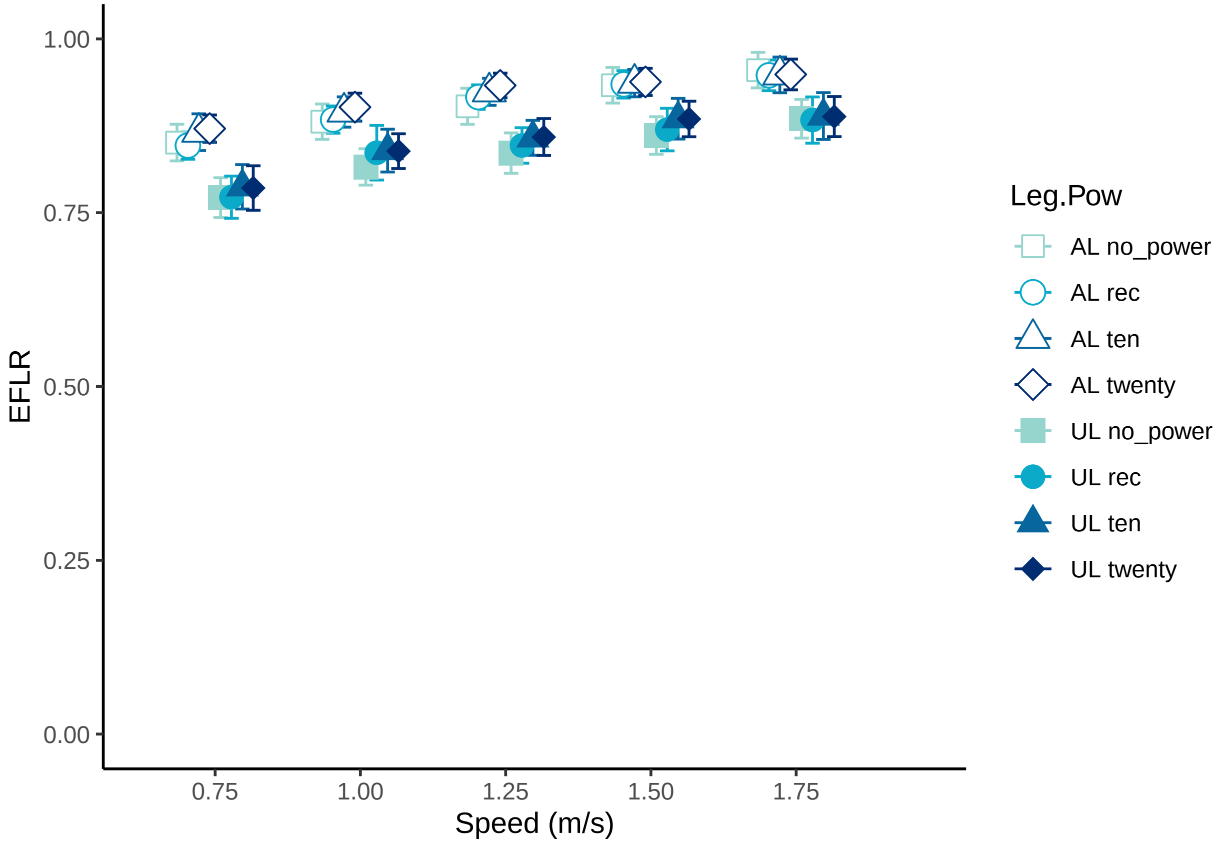

Combined Figure 4

```
fig317 <- grid.arrange(eflr_stiff + theme(legend.position = "none"), eflr_power + theme(legend.position
```

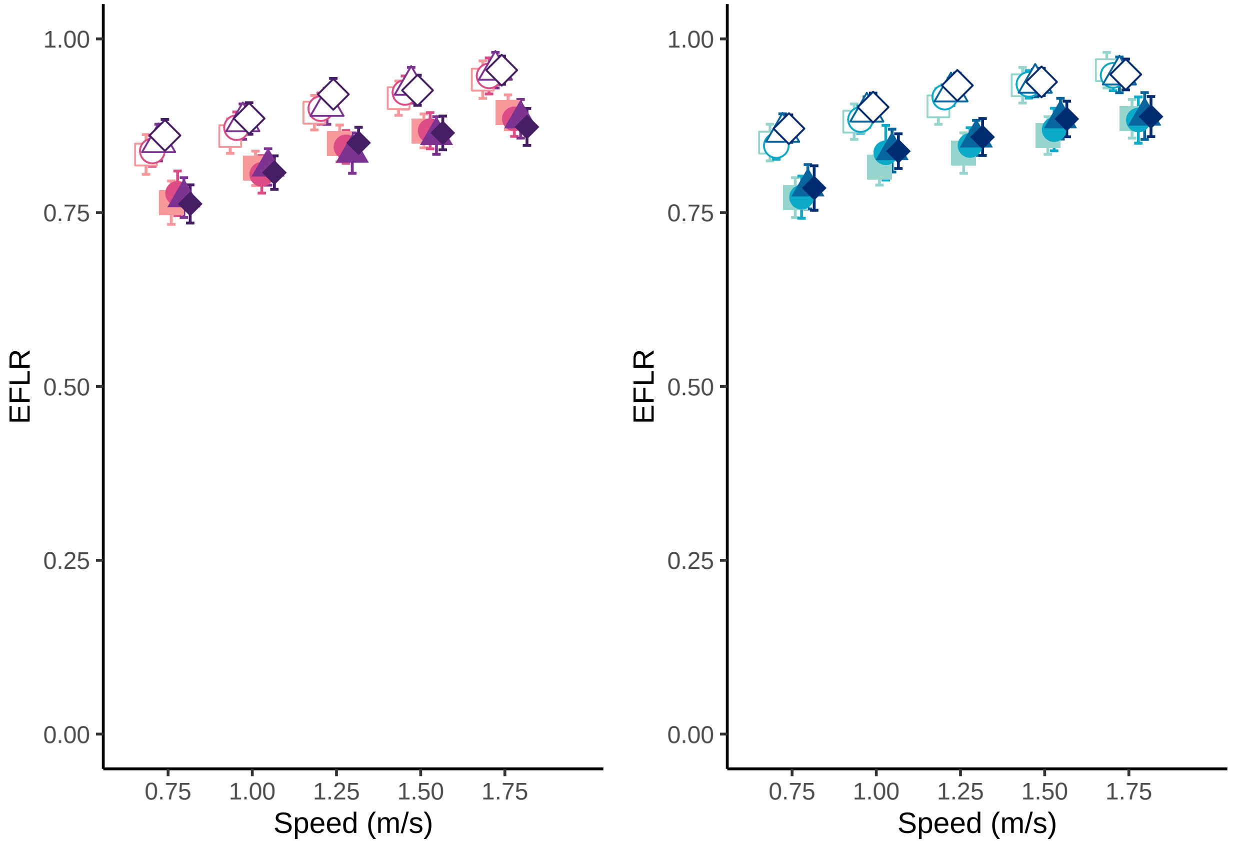


```
fig317
```

```
## TableGrob (1 x 2) "arrange": 2 grobs
##   z     cells    name           grob
## 1 1 (1-1,1-1) arrange gtable[layout]
## 2 2 (1-1,2-2) arrange gtable[layout]
```

```
ggsave("fig317.pdf", plot = fig317, device = "pdf", useDingbats = FALSE, width = 6.7, height = 3.2, uni
```

Appendix Figures that Show Interaction of Stiffness and Power

AL to UL Step-to-Step Transition

Appendix Figure 1a

```
#Positive AL work vs. Speed (Grouped by Power Setting; Each graph is for different stiffness category)

ps_data_al2ul <- ps_data[ps_data$Transition == 'AL2UL',] #AL to UL step-to-step transition

pos_work_power_stiff_al2ul <- ggplot(data = ps_data_al2ul, aes(x = Speed, y = Wpos_trail, col = Power, 
  #geom_jitter(alpha = 0.3)+
  stat_summary(geom="errorbar", fun.data = mean_se, width = 0.2, position=position_dodge(width=0.15)) +
  stat_summary(geom="point", fun = mean, size = 4, position = position_dodge(width=0.15), fill = "white
  labs(x = "Speed (m/s)", y = "Work (J)")+
  scale_x_continuous(limits = c(0.625, 1.975), breaks = c(0.75, 1.00, 1.25, 1.50, 1.75))+
```

```
  expand_limits(y= c(0,40))+
  scale_shape_manual(values = c(22, 21, 24, 23))+
  scale_color_manual(values = bl) +
  facet_wrap(vars(Stiff_cat), ncol = 4)+
  theme_classic()

pos_work_power_stiff_al2ul
```

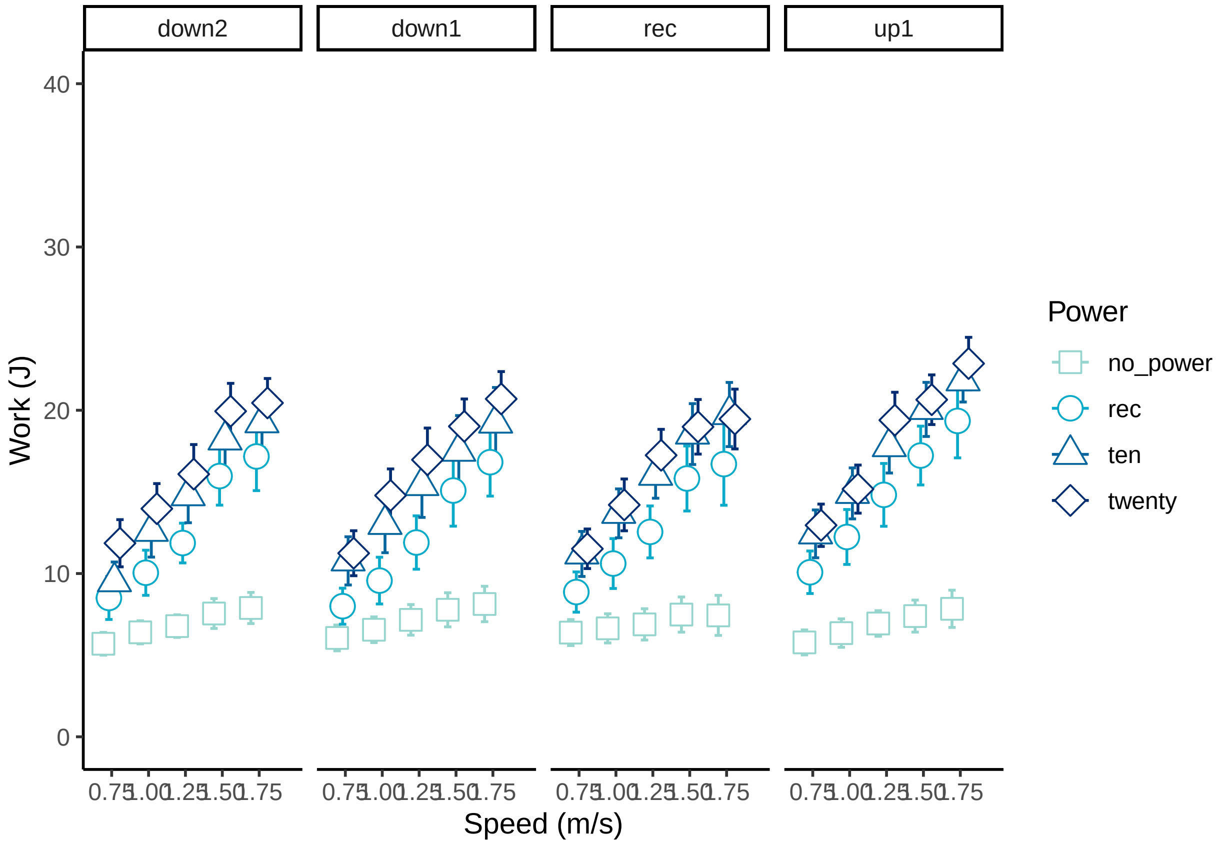

Appendix Figure 1b

```
ps_data_al2ul <- ps_data[ps_data$Transition == 'AL2UL',] #AL to UL step-to-step transition

neg_work_power_stiff_al2ul <- ggplot(data = ps_data_al2ul, aes(x = Speed, y = Wneg_lead, col = Power, sl
  stat_summary(geom="errorbar", fun.data = mean_se, width = 0.2, position=position_dodge(width=0.15)) +
  stat_summary(geom="point", fun = mean, size = 4, position = position_dodge(width=0.15))+
  labs(x = "Speed (m/s)", y = "Work (J)")+
  scale_x_continuous(limits = c(0.625, 1.975), breaks = c(0.75, 1.00, 1.25, 1.50, 1.75), position = "to
  expand_limits(y= c(-40,0))+
  scale_shape_manual(values = c(15, 16, 17, 18))+
  scale_color_manual(values = bl) +
  facet_wrap(vars(Stiff_cat), ncol = 4)+
  theme_classic()

neg_work_power_stiff_al2ul
```

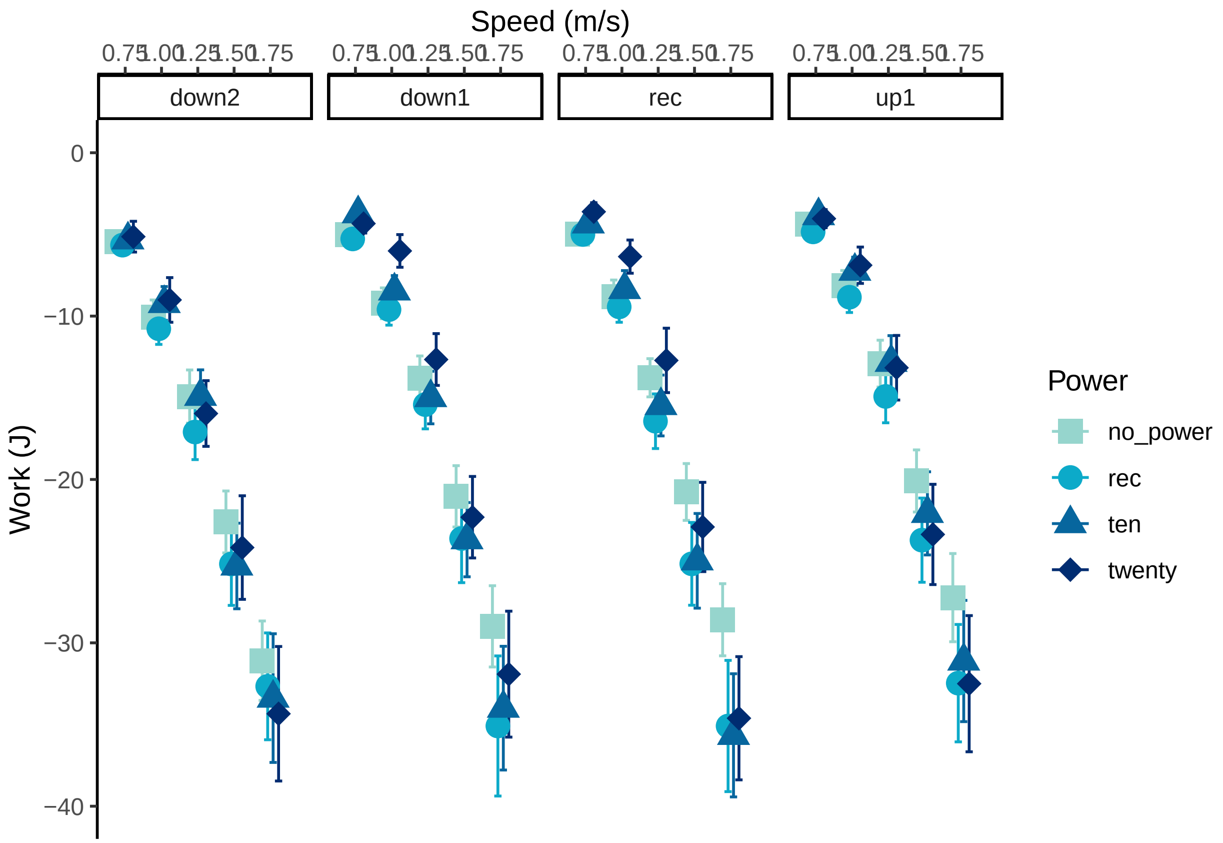

Combined Appendix Figure 1

```
appen311 <- grid.arrange(pos_work_power_stiff_al2ul + theme(legend.position = "none"), neg_work_power_s
```

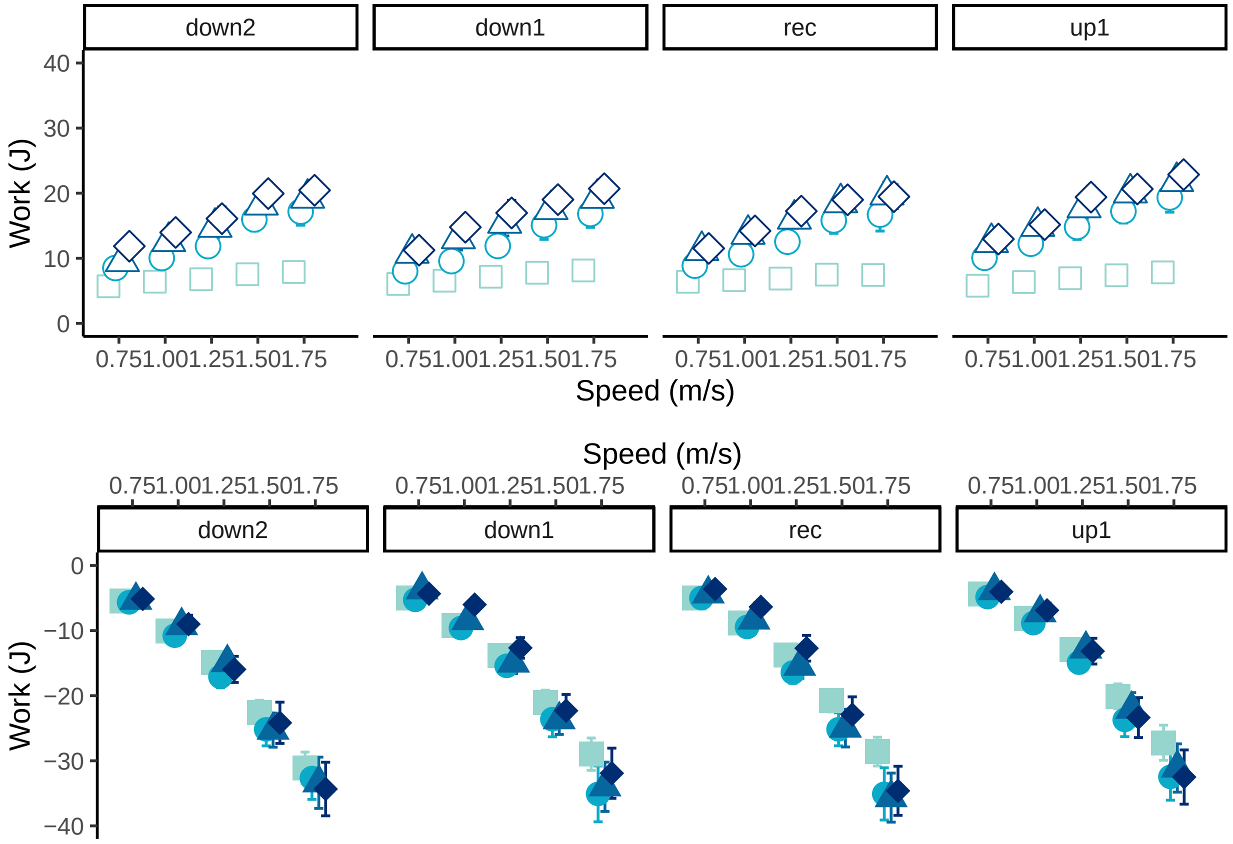


```
appen311
```

```
## TableGrob (2 x 1) "arrange": 2 grobs
##   z     cells    name           grob
## 1 1 (1-1,1-1) arrange gtable[layout]
## 2 2 (2-2,1-1) arrange gtable[layout]
```

```
ggsave("appen311.pdf", plot = appen311, device = "pdf", useDingbats = FALSE, width = 6.5, height = 7, u
```

UL to AL Step-to-Step Transition

Appendix Figure 2a

```
#Positive UL work vs. Speed (Grouped by Power setting)

ps_data_ul2al <- ps_data[ps_data$Transition == 'UL2AL',] #UL to AL step-to-step transition

pos_work_power_stiff_ul2al <- ggplot(data = ps_data_ul2al, aes(x = Speed, y = Wpos_trail, col = Power, 
  #geom_jitter(alpha = 0.3)+
  stat_summary(geom="errorbar", fun.data = mean_se, width = 0.2, position=position_dodge(width=0.15)) +
  stat_summary(geom="point", fun = mean, size = 4, position = position_dodge(width=0.15))+
  labs(x = "Speed (m/s)", y = "Work (J)")+
  scale_x_continuous(limits = c(0.625, 1.975), breaks = c(0.75, 1.00, 1.25, 1.50, 1.75))+
  expand_limits(y= c(0,40))+
  scale_shape_manual(values = c(15, 16, 17, 18))+
```

```
  scale_color_manual(values = bl) +
  facet_wrap(vars(Stiff_cat), ncol = 4)+
  theme_classic()

pos_work_power_stiff_ul2al
```

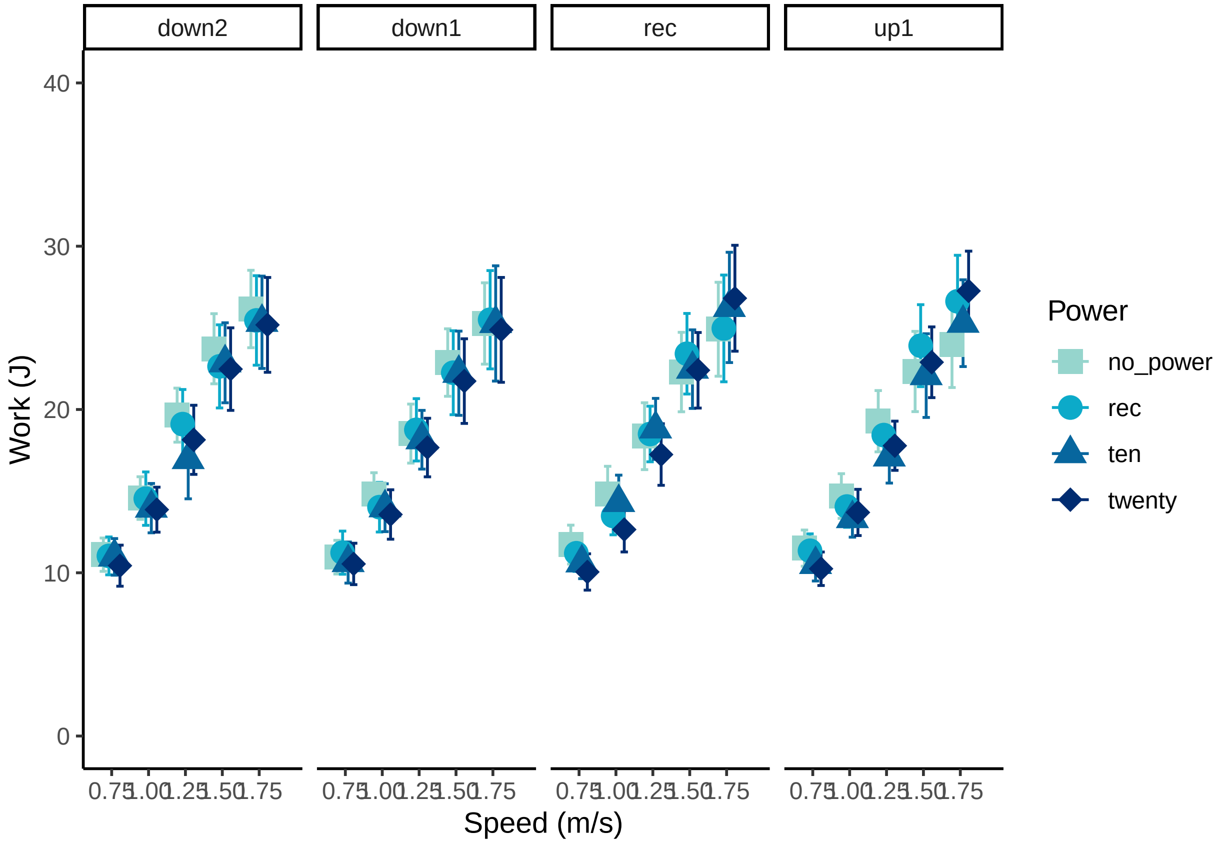

Appendix Figure 2b

```
#Negative AL work vs. Speed (Grouped by Power setting)

ps_data_ul2al <- ps_data[ps_data$Transition == 'UL2AL',] #UL to AL step-to-step transition

neg_work_power_stiff_ul2al <- ggplot(data = ps_data_ul2al, aes(x = Speed, y = Wneg_lead, col = Power, sh
  stat_summary(geom="errorbar", fun.data = mean_se, width = 0.2, position=position_dodge(width=0.15)) +
  stat_summary(geom="point", fun = mean, size = 4, position = position_dodge(width=0.15), fill = "white
  labs(x = "Speed (m/s)", y = "Work (J)")+
  scale_x_continuous(limits = c(0.625, 1.975), breaks = c(0.75, 1.00, 1.25, 1.50, 1.75), position = "top
  expand_limits(y= c(-40,0))+
  scale_shape_manual(values = c(22, 21, 24, 23))+
  scale_color_manual(values = bl) +
  facet_wrap(vars(Stiff_cat), ncol = 4)+
  theme_classic()

neg_work_power_stiff_ul2al
```

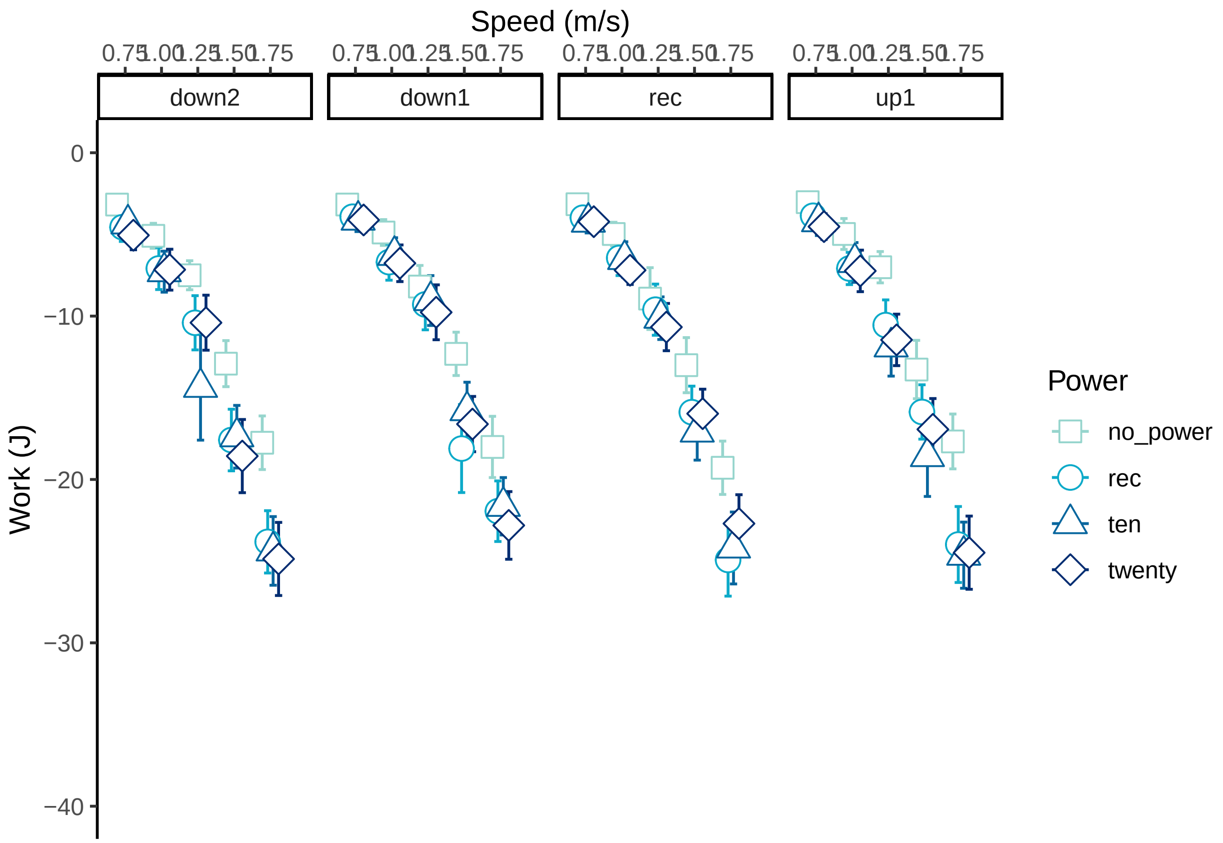

Combined Appendix Figure 2

```
appen312 <- grid.arrange(pos_work_power_stiff_ul2al + theme(legend.position = "none"), neg_work_power_st
```

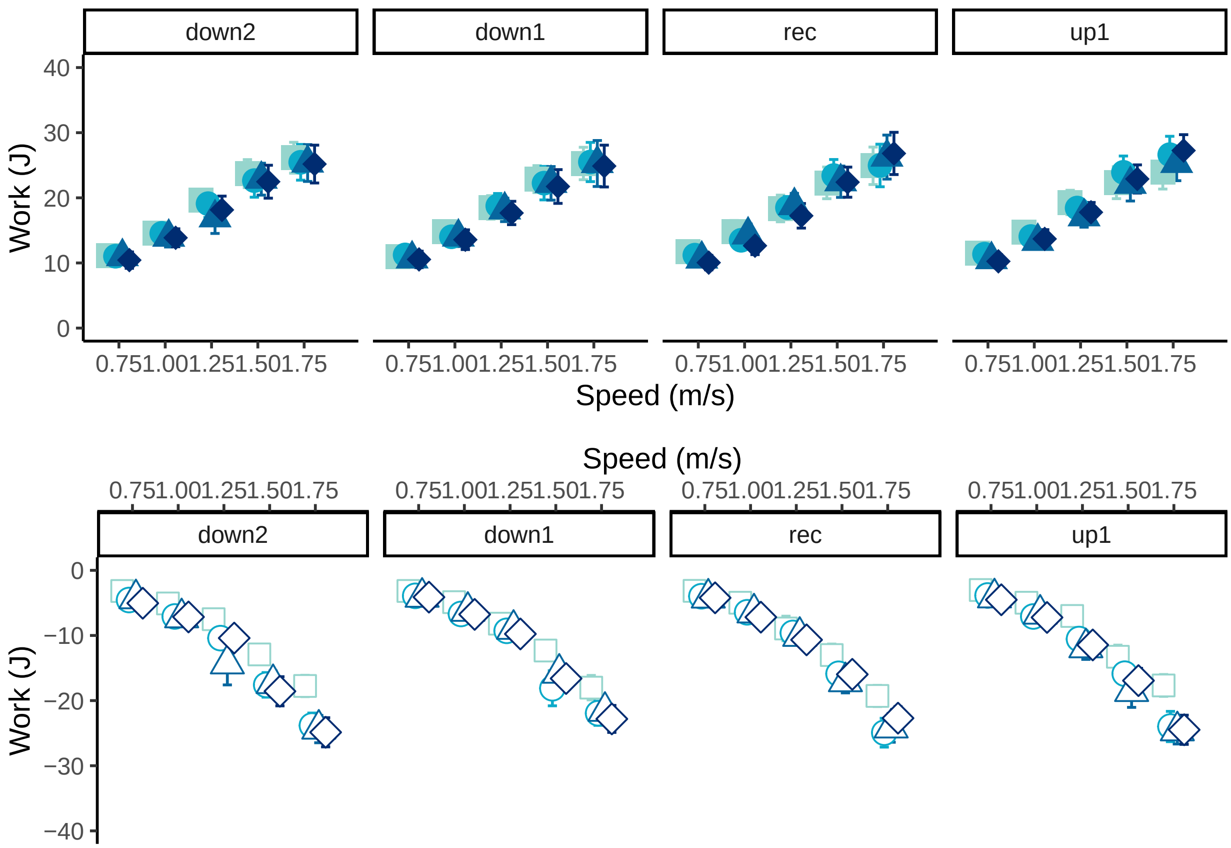


```
appen312
```

```
## TableGrob (2 x 1) "arrange": 2 grobs
##   z     cells    name           grob
## 1 1 (1-1,1-1) arrange gtable[layout]
## 2 2 (2-2,1-1) arrange gtable[layout]
```

```
ggsave("appen312.pdf", plot = appen312, device = "pdf", useDingbats = FALSE, width = 6.5, height = 7, u
```

Roll-over Shape (EFLR)

Appendix Figure 3

```
#EFLR (Grouped by Power Setting and Leg)
eflr_power_stiff <- ggplot(data = ps_data, aes(x = Speed, y = EFLR, col = Leg.Pow, shape = Leg.Pow))+
  #geom_jitter(alpha = 0.3)+
  stat_summary(geom="errorbar", fun.data = mean_se, width = 0.2, position=position_dodge(width=0.15)) +
  stat_summary(geom="point", fun = mean, size = 4, position = position_dodge(width=0.15), fill = "white
  labs(x = "Speed (m/s)", y = "EFLR")+
  scale_x_continuous(limits = c(0.625, 1.975), breaks = c(0.75, 1.00, 1.25, 1.50, 1.75))+
  expand_limits(y= c(0,1))+
  scale_shape_manual(values = c(22, 21, 24, 23, 15, 16, 17, 18))+
  scale_color_manual(values = c(bl, bl)) +
  facet_wrap(vars(Stiff_cat), ncol = 4)+
  theme_classic()
```

```
eflr_power_stiff
```

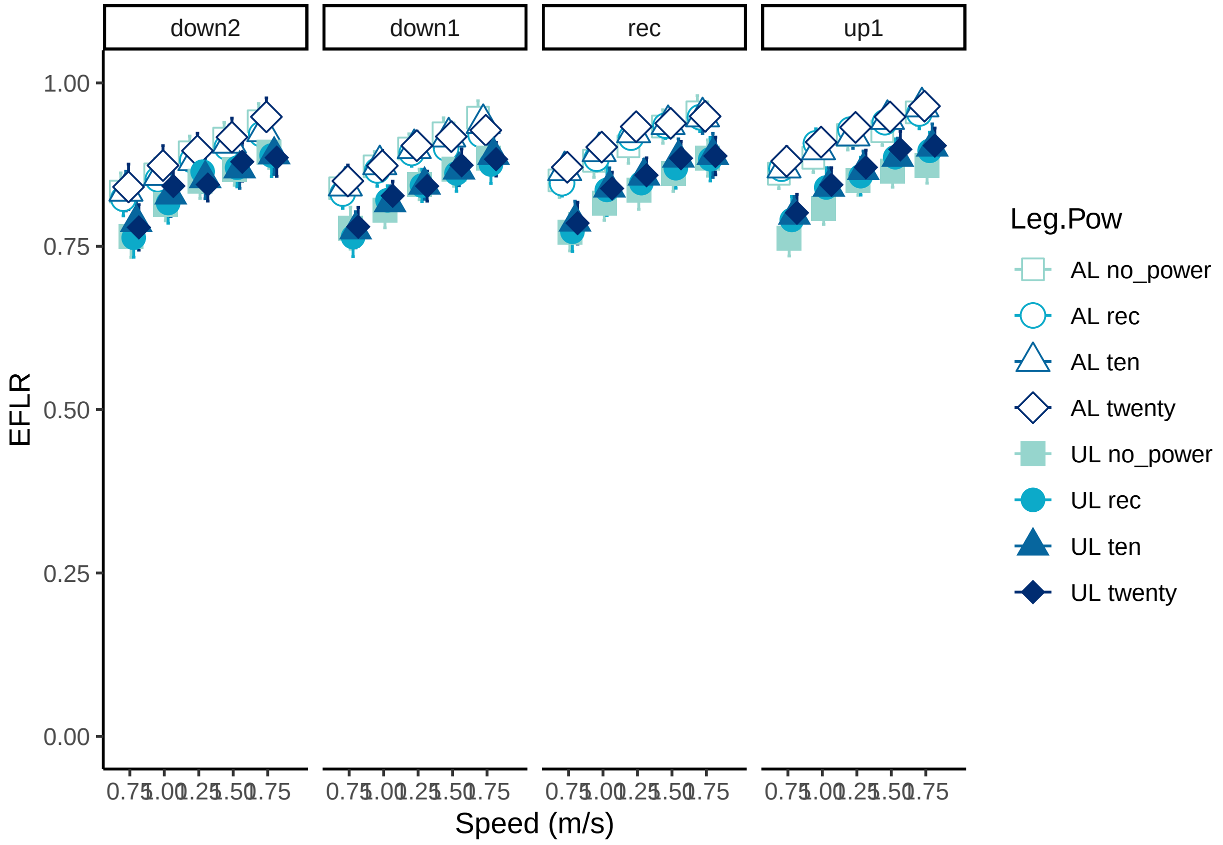


```
ggsave("appen313.pdf", plot = eflr_power_stiff + theme(legend.position = "none"), device = "pdf", useDi
```
