# Supplementary material for: Greater than recommended stiffness and power setting of a stance-phase powered leg prosthesis can improve step-to-step transition work and effective foot length ratio during walking in people with transtibial amputation
Source: Front Bioeng Biotechnol. 2024 Jul 1;12:1336520. doi: 10.3389/fbioe.2024.1336520 (PMC11246994; doi:10.3389/fbioe.2024.1336520)
Supplement: Supplementary file 3 [file DataSheet1.docx]

Notes on Tacca_data.csv

- Column 1: Sub
  - Subject ID; corresponds with participant number in Table 1
- Column 2: Mass
  - Body mass in kg including the prosthesis; for trials with the passive-elastic prosthesis (power column is “no power”), body mass includes the LP Vari-flex prosthesis; for other trials, body mass includes the BiOM
- Column 3: Stiff_cat
  - Stiffness category of the Össur low profile (LP) Vari-flex prosthesis relative to recommended; two categories less stiff than recommended (down2), one category less stiff than recommended (down1), recommended (rec), and one category stiffer than recommended (up1)
- Column 4: Power
  - Power setting; trials without the BiOM (no_power), trials with the BiOM at recommended power setting (rec), trials with the BiOM at +10% power setting (ten), and trials with the BiOM at +20% power setting (twenty)
- Column 5: Speed
  - Speed in m/s
- Column 6: Leg
  - Unaffected leg (UL) or affected leg (AL); for use with effective foot length ratio (EFLR) column
- Column 7: Transition
  - Step-to-step transition; unaffected to affected leg transition (UL2AL) or affected to unaffected leg transition (AL2UL); for use with mechanical work columns
- Column 8: Wpos
  - Total positive mechanical work done during the step-to-step transition in J
- Column 9: Wneg
  - Total negative mechanical work done during the step-to-step transition in J
- Column 10: Wpos_trail
  - Positive mechanical work done by the trailing leg during the step-to-step transition in J
- Column 11: Wpos_lead
  - Positive mechanical work done by the leading leg during the step-to-step transition in J
- Column 12: Wneg_trail
  - Negative mechanical work done by the trailing leg during the step-to-step transition in J
- Column 13: Wneg_lead
  - Negative mechanical work done by the leading leg during the step-to-step transition in J
- Column 14: EFLR
  - Effective foot length ratio
- Column 15: Cat_num
  - Stiffness category of LP Vari-flex prosthesis; category 1-9
- Column 16: Size
  - Size of the LP Vari-flex prosthesis in cm
- Column 17: Heel_stiff
  - Average axial stiffness of the heel of LP Vari-flex prosthesis without a shoe in kN/m; measured from mechanical testing in Tacca et al. *Front Rehabil Sci* 2024
- Column 18: Fore_stiff
  - Average axial stiffness of the forefoot of the LP Vari-flex prosthesis without a shoe in kN/m; measured from mechanical testing in Tacca et al. *Front Rehabil Sci* 2024
